# *Curcuma longa* and *Boswellia serrata* Extracts Modulate Different and Complementary Pathways on Human Chondrocytes *In Vitro*: Deciphering of a Transcriptomic Study

**DOI:** 10.3389/fphar.2022.931914

**Published:** 2022-08-11

**Authors:** Christelle Sanchez, Jérémie Zappia, Cécile Lambert, Jacques Foguenne, Yvan Dierckxsens, Jean-Emile Dubuc, Jean-Pierre Delcour, André Gothot, Yves Henrotin

**Affiliations:** ^1^ MusculoSKeletal Innovative Research Lab, Center for Interdisciplinary Research on Medicines, University of Liège, Liège, Belgium; ^2^ Center for Interdisciplinary Research on Medicines, University of Liège, Liege, Belgium; ^3^ Department of Laboratory Hematology, Liege University Hospital, Liege, Belgium; ^4^ Tilman SA, Baillonville, Belgium; ^5^ Cliniques Universitaires de St Luc, Brussels, Belgium; ^6^ Centre Hospitalier Du Bois de L’Abbaye, Seraing, Belgium; ^7^ Physical Therapy and Rehabilitation Department, Princess Paola Hospital, Marche-en-Famenne, Belgium

**Keywords:** osteoarthritis, transcriptomic (RNA-seq), *Curcuma*, *Boswellia*, chondrocyte

## Abstract

**Objectives:** *Curcuma longa* (CL) and *Boswellia serrata* (BS) extracts are used to relieve osteoarthritis symptoms. The aim of this *in vitro* study was to investigate their mechanisms of action at therapeutic plasmatic concentrations on primary human osteoarthritic (OA) chondrocytes.

**Methods:** BS (10–50 μg/ml) and CL (0.4–2 μg/ml corresponding to 1–5 µM of curcumin) were evaluated separately or in combination on primary chondrocytes isolated from 17 OA patients and cultured in alginate beads. Ten patients were used for RNA-sequencing analysis. Proteomic confirmation was performed either by immunoassays in the culture supernatant or by flow cytometry for cell surface markers after 72 h of treatment.

**Results:** Significant gene expression modifications were already observed after 6 h of treatment at the highest dose of CL (2 μg/ml) while BS was significantly effective only after 24 h of treatment irrespective of the concentration tested. The most over-expressed genes by CL were anti-oxidative, detoxifying, and cytoprotective genes involved in the *Nrf2* pathway. Down-regulated genes were principally pro-inflammatory cytokines and chemokines. Inversely, BS anti-oxidant/detoxifying activities were related to the activation of *Nrf1* and PPARα pathways. BS anti-inflammatory effects were associated with the increase in GDF15, decrease in cholesterol cell intake and fatty acid metabolism-involved genes, and down-regulation of Toll-like receptors (TLRs) activation. Similar to CL, BS down-regulated ADAMTS1, 5, and MMP3, 13 genes expression. The combination of both CL and BS was significantly more effective than CL or BS alone on many genes such as IL-6, CCL2, ADAMTS1, and 5.

**Conclusion:** BS and CL have anti-oxidative, anti-inflammatory, and anti-catabolic activities, suggesting a protective effect of these extracts on cartilage. Even if they share some mechanism of action, the two extracts act mainly on distinct pathways, and with different time courses, justifying their association to treat osteoarthritis.

## Introduction

Osteoarthritis (OA) is the most common form of arthritis, affecting millions of people worldwide. It is a serious disorder primarily affecting weight-bearing joints characterized by cell stress and extracellular matrix degradation initiated by micro- and macro-injury that activates maladaptive repair responses, including pro-inflammatory pathways of innate immunity (Mobasheri et al., 2019). Natural extracts from *Curcuma longa* and *Boswellia serrata* are known for their anti-oxidative and anti-inflammatory properties and are used to relieve clinical symptoms of OA for many years (Bannuru et al., 2018; Henrotin et al., 2019). Curcumin is the principal curcuminoid extracted from the *C. longa* (turmeric) root and has revealed a broad spectrum of bioactivities, including anti-oxidant, anti-inflammatory, anti-tumor, and anti-viral activities. At the molecular level, this multitargeted agent has been shown to act through numerous cell signaling pathways: the PI3K/Akt-1/mTOR, the Ras/Raf/MEK/ERK, the GSK-3β, the p53, and *via* NF-κB, Akt, and Nrf2/ARE pathways (Kunnumakkara et al., 2017; Jiang et al., 2020; Li et al., 2020; Pauletto et al., 2020). Recently, it was shown to increase the nuclear expression levels and promote the biological effects of Nrf2 *via* the interaction with Cys151 in Keap1, which makes it a therapeutic candidate against a broad range of oxidative stress-related diseases (Rahban et al., 2020). Furthermore, curcumin inhibits lipo-oxygenase (LOX) and cyclo-oxygenase (COX), xanthine oxygenase activities, nitric oxide synthesis, ROS generation, and pro-inflammatory cytokines and chemokines release (Mathy-Hartert et al., 2009; Comblain et al., 2015; Comblain et al., 2016; Rahban et al., 2020; Mohan et al., 2021). Boswellic acids, a class of triterpenes, are the bioactive constituents of *B. serrata* extracts. In the resin, more than 12 different boswellic acids have been identified. Their anti-inflammatory actions are caused by different mechanisms of action. They include inhibition of leukotriene, and to a less extent prostaglandin synthesis, inhibition of the complement system at the level of conversion of C3 into C3a and C3b, the decreased production of proinflammatory cytokines including IL-1, IL-2, IL-6, IFN-γ, and TNF-α, inhibition of formation of oxygen radicals and lysosomal enzymes, and the suppression of the proteolytic activity of cathepsin G and elastase (Tausch et al., 2009; Ammon 2016).

Two recent meta-analyses evaluating a large number of dietary supplements ranked *C. longa* and *B. serrata* among the most effective compounds for pain reduction in OA in the short term, although the quality of evidence was low (Bannuru et al., 2018; Liu et al., 2018). Altogether, these elements give a good rationale to combine *C. longa* and *B. serrata* to relieve joint symptoms in OA. To our knowledge, few studies have investigated the combination of *C. longa* and *B. serrata* in the management of OA (Haroyan et al., 2018). This combination is not yet currently used in daily practice to treat OA, but Haroyan’s study has shown that this combination gives better efficacy of OA treatment than *C. longa* alone on WOMAC pain score, presumably due to synergistic effects of curcumin and boswellic acid.

Both curcumin and boswellic acids possess common interesting pharmacological properties such as the inhibition of cyclooxygenase, lipoxygenase, and pro-inflammatory cytokines, but the whole activities of these compounds have not been investigated yet in chondrocytes by transcriptomic using RNA-seq. This study compared the transcriptomic profile of human primary OA chondrocytes cultured in alginate beads and treated with *C. longa* and *B. serrata* extracts separately or in combination. The objective was to investigate if it was interesting or not to combine these extracts in OA treatment.

## Material and Methods

### Ethical Statement

Articular cartilage samples from 17 successive patients with knee OA were obtained at the time of total knee joint replacement (TKR) surgery. All participants have signed the informed patient consent, and the protocol was approved by the ethical committee of the University of Liège (B70720108313). All procedures followed the ethical standards of the responsible committee on human experimentation (institutional and national) and with the Helsinki Declaration of 1975, revised in 2000. Supplementary File S1 gives an overview of the patients’ characteristics and their use in this study.

### Study Medication

*C. longa* extract (CL, containing 89.6% of curcumin and 9.1% of demethoxycurcumin) and *B. serrata* oleoresin (BS, containing 2.83% 3-acetyl-11-keto-β-boswellic acid, 4.35% 11-keto-β-boswellic acid, 10.17% α-boswellic acid, 15.5% β-boswellic acid, 2.24% acetyl α-boswellic acid, and 6.63% acetyl-β boswellic acid) were used. These extracts were provided by Tilman SA (Belgium) and stored at ambient temperature, protected from light. Before use, the extracts were solubilized in tetrahydrofuran at 4 mg/ml (CL) or 100 mg/ml (BS). They were further diluted in tetrahydrofuran to achieve a final 0.1% of solvent in each culture condition. We evaluated two concentrations for each extract, corresponding to the minimal and the maximal concentrations of the active compounds found in the plasma after oral treatment with these extracts (Buchele and Simmet 2003; Henrotin et al., 2013).

### Study Design

Supplementary File S2 gives a detailed overview of the study design. In short, for transcriptome analysis, chondrocytes were untreated (control) or treated with CL at 0.4 or 2 μg/ml, BS at 10 or 50 μg/ml, or a combination of CL and BS (CL/BS) at low or high concentration during 6 and 24 h. For protein analysis, chondrocyte supernatant was used to quantify IL-6, CCL2, NO_2_, and GDF15 using specific immunoassays or Griess reaction and standardized according to each culture well DNA content on each culture condition performed in triplicate. Flow cytometry was performed on five other chondrocyte cultures to evaluate the presence of TLR1-2-4-6 on the cell surface.

### Chondrocyte Alginate Beads Culture

Full-depth articular cartilage was excised, chondrocytes were enzymatically isolated and suspended in alginate beads at 4 million cells per ml as previously described (Sanchez et al., 2021), and cultured for 3 days in Dulbecco’s modified Eagle’s medium supplemented with 10% FBS, 10 mM HEPES, penicillin (100 U/ml) and streptomycin (100 μg/ml), 200 μg/ml glutamine, 50 μg/ml ascorbic acid, and 2 mM proline (all were obtained from Biowest, France, except for ascorbic acid and proline, which were from Sigma-Aldrich, Germany). Alginate beads containing chondrocytes were placed in 24-well plates, nine beads per well. Three wells were used per time point and treatment. After the first 3 days, the culture media were replaced by a fresh one with the same composition but containing CL, BS, CL/BS, or tetrahydrofuran 0.1% alone for untreated control as described in the study design.

For transcriptomic analysis, after 6 and 24 h of treatment, alginate beads were transferred to cold 0.1 M citrate for dissolution and, after centrifugation, cells were homogenized in lysis buffer for ribonucleic acid extraction, and then stored frozen at −80°C until RNA extraction (Qiagen, Netherlands).

After 72 h, cell cultures were stopped, and supernatants were collected and stored at −20°C for further analysis. For protein content measurement by immunoassays, cells were homogenized in 1 ml of Tris-HCl buffer by ultrasonic dissociation for 20 s at 4°C to measure total DNA content.

### Flow Cytometry

All reagents used for the flow cytometry were purchased from BD Biosciences (France) unless indicated otherwise. Alginate beads from two wells were pooled and dissolved in 2 ml 0.1 M citrate containing 1 µl LIVE/DEAD green stain kit (Invitrogen, United States) at 4°C and protected from light. After 25 min incubation, chondrocytes were centrifuged 5 min at 1200 rpm, rinsed in 1 ml NaCl 0.9 g/L at 4°C, centrifuged for 5 min at 1200 rpm, and suspended in 300 µl NaCl 0.9 g/L. One hundred microliters of cell suspension was transferred to polypropylene tubes and incubated with 5 µl of FcBlock for 10 min at room temperature to block any unspecific binding. Then, 50 µl of Brilliant stain buffer and 5 µl of each specific antibody were added. Antibodies used were mouse IgG anti-human TLR1 (BV421-conjugated, 566430), TLR2 (Alexa fluor 647-conjugated, 558319), TLR4 (BB700-conjugated, 745946), and TLR6 (PE-conjugated, 566339). For each sample and each treatment, one tube with all control isotypes and one other tube with no antibody were also performed in parallel to measure the non-specific binding and autofluorescence of the cells. After 20 min of incubation at room temperature and protection from light, cells were washed and suspended in 400 µl (in BD lyse wash assistant) before being read for fluorescence in flow cytometry (BD FACS Canto II). A total of 3000 to 5000 living cells (following LIVE/DEAD staining in 488 nm) were analyzed per sample. The difference between the median fluorescence and the median autofluorescence in each channel was calculated for each sample to obtain the median fluorescence intensity. A cut-off placed at the end of the autofluorescence peak was also used to estimate the percentage of highly positive cells in each sample.

### DNA and NO_2_ Quantification

DNA content of cell culture was measured according to a fluorometric method (Labarca and Paigen 1980). Nitric oxide (NO) production was determined by quantifying its derived product, nitrite, in the culture supernatant using a spectrophotometric method based on the Griess reaction (Green et al., 1982).

### RNA Extraction

Total RNA was extracted using an RNeasy mini kit (Qiagen, Netherlands) according to the instructions of the manufacturer. The yield of the extracted RNA was determined spectrophotometrically by measuring the optical density at 260 nm. The purity and quality of extracted RNA were further evaluated using an RNA Nano 6000 Bioanalyzer Agilent (Santa Clara, United States) according to the manufacturer’s instructions. High-quality RNAs with RNA quality indicator scores (RIN) of >8 were used.

### RNA-Seq and Differential Gene Expression Analysis

One hundred nanograms of RNA from each culture condition was used for this analysis. Libraries were prepared with the Illumina Truseq stranded mRNA sample prep kit according to the manufacturer’s instructions. Poly(A) plus RNA was enriched using oligo(dT) beads followed by fragmentation and reverse transcription. Afterward, the 5′ and 3′ ends of cDNA fragments were prepared to ensure efficient ligation of “Y” adapters containing unique barcode and primer binding sites. Finally, ligated cDNAs were PCR amplified to be ready for cluster generation and sequencing.

Sequencing was performed on Novaseq (Illumina), paired-end reads (150-10-10-150), and NovaSeq S4 V1.5 300 cycles XP workflow, generating around 20 M reads per sample. Denaturated NGS library fragments were flowed across a flow cell and hybridized on a lawn of complementary Illumina adapter oligos. Complementary fragments were extended, amplified *via* bridge amplification PCR, and denaturated, resulting in clusters of identical single-stranded library fragments. Fragments were primed and sequenced utilizing reversible terminator nucleotides. Base pairs were identified after laser excitation and fluorescence detection.

Raw data were demultiplexed into individual libraries. After filtering out reads mapping to rRNA, tRNA, mitochondrial RNA, and other contaminants (e.g., adapters, *etc*.) using bowtie2, reads were aligned onto the human reference genome (GRCh38 and Ensembl 105 annotation) and quantified with Star to give the Counts file. Quality control of sequencing reads was assessed with FASTQC and quality control after mapping with Picard tools. Compilation of tool metrics was performed with MultiQC.

Differential expression analysis was made in R (version 4.1.2, https://www.R-project.org/) using the DESeq2 package (1.34.0) (Love et al., 2014), biomaRt package (version 2.50.1) (Durinck et al., 2009), and R code design = ∼ Patient + Treatment. Analysis was performed with treatment as a contrast: each treated vs. control and the combination vs. each extract separately. Volcano plots and Venn diagrams were generated using ggplot2 (version 3.3.5) and nVennR (version 0.2.3) (Perez-Silva et al., 2018). Gene set enrichment analysis (GSEA) was performed using clusterProfiler package (4.2.0) (Wu et al., 2021), rWikipathways (1.14.0) (Slenter et al., 2018), and visualized on KEGG diagrams with pathview (1.34.0, considering only DEGs with padj <0.01) (Luo and Brouwer 2013).

A false discovery rate (FDR) of 0.01 was used to assess the statistical significance.

### ELISA for IL-6, CCL2, and GDF15

Protein amount of IL-6, CCL2, and GDF15 was measured from the supernatant, by specific enzyme-amplified sensitivity immunoassays (IL-6: Invitrogen cytoset CHC1263; CCL2: R&Dsystems UK DuoSet DY279; GDF15: R&Dsystems UK DuoSet DY957). Protein content was normalized to the DNA content.

### Statistical Analysis

For the transcriptome analysis, the DESeq2 Bioconductor package was used for normalization, principal component analysis (PCA), and differential gene expression. DESeq2 differential gene analysis was based on the hypothesis that most genes were not differentially expressed (Anders and Huber 2010; Love et al., 2014). The method was based on the negative binomial distribution model. Within the DESeq2 package, and with the *estimate SizeFactorsForMatrix* function, scaling factors were calculated for each run. After dividing gene counts by each scaling factor, DESeq2 values were calculated as the total of rescaled gene counts of all runs.

The amplitude of changes is represented either in the log2foldchange format (classical representation from DESeq2, where “0” means “no change,” “1” means “2-fold induction,” and “−1” means “2-fold decrease”) or in fold change (where “1” means “no change,” “2” means “2-fold induction,” and “0.5” means “2-fold decrease”).

Along with the standard *p*-value, an adjusted *p*-value (padj) was calculated. The adjustment methods included the Bonferroni correction (“Bonferroni”) in which the *p*-values were multiplied by the number of comparisons. Less conservative corrections were also included by Holm (“holm”) (Holm 1979), Hochberg (“hochberg”) (Hochberg 1988), Hommel (“hommel”) (Hommel 1988), Benjamini & Hochberg (“BH” or its alias “fdr”) (Benjamini and Hochberg 1995), and Benjamini & Yekutieli (“BY”) (Benjamini and Yekutieli 2001), respectively. The “BH” (aka “FDR”) and “BY” methods of Benjamini, Hochberg, and Yekutieli control the false discovery rate, i.e., the expected proportion of false discoveries among the rejected hypotheses.

To avoid any confusion coming from a threshold effect on padj and log2FC, we compared gene by gene the log2FC at different times and treatments for each patient using ANOVA and Bonferroni post-test to detect significant accentuations between the two conditions (Supplementary Files S4, S9).

For the protein analyses, results were statistically analyzed using GraphPad Prism 6.0. The mean is calculated for the triplicate of each cell culture (*n* = 12) or delta of median fluorescence and median autofluorescence for flow cytometry (*n* = 5). These values will be first analyzed in GraphPad Prism 6.0 to check their normality (D’Agostino & Pearson omnibus test). Then, paired one-way ANOVA was used to compare each treatment to the control, with multiple-comparison correction (Holm–Sidak’s) or Friedman test for repeated measures with Dunn’s post-test, to calculate the statistical significance.

## Results

### Global RNA-Seq Analysis of Differentially Expressed Genes (DEGs)

**FIGURE 1:**
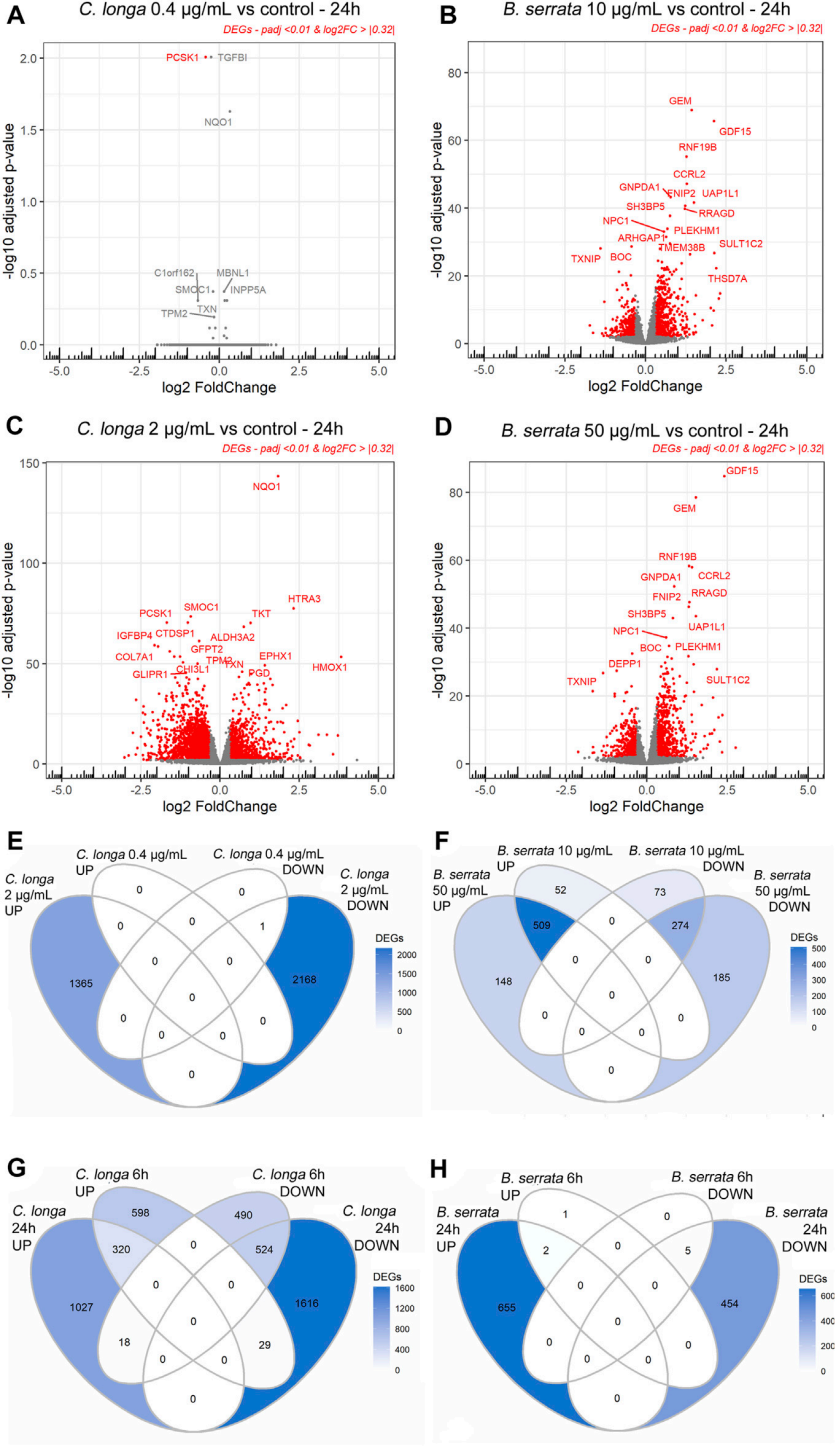
Volcano plots **(A**–**D)** and Venn diagrams **(E**–**H)** of *C. longa* 0.4 and 2 μg/ml and *B. serrata* 10 and 50 μg/ml after 24 h treatment on OA chondrocytes **(A**–**F)** or *C. longa* 2 μg/ml and *B. serrata* 50 μg/ml at 6 vs. 24 h **(G,H)**. Threshold *p*adj <0.01 and Log2FoldChange >|0.32|, *n* = 10.

After 6 h of treatment, 3 DEGs were observed with CL 0.4 μg/ml, 1932 with CL 2 μg/ml, 13 with BS 10 μg/ml, and 8 with BS 50 μg/ml in OA chondrocytes compared to the untreated control, respectively, with a padj <0.01 and a Log2FoldChange |>0.32| corresponding to a fold change of at least or >33% more or >25% less (Figure 1 and Supplementary File S3).

After 24 h of treatment, CL at 2 μg/ml and BS at 50 μg/ml significantly modified the expression of 3534 and 1116 genes as shown on volcano plots in Figure 1. Only one gene was significantly modulated by CL at 0.4 μg/ml (Supplementary File S4). Using DESeq2, no significant DEG was found between both BS concentrations. Padj of the DEGs w lower at 50 compared to 10 μg/ml but log2FC was not modified (Figures 1B,D). Complete DESeq2 analysis between treatments after 24 h is listed in Supplementary File S4. An ANOVA analysis showed that after 24 h of treatment, 161 DEGs were significantly more up- or down-regulated than after 6 h and that 47 genes switched from up- to down-regulation or inversely (Supplementary File S5).

Based on these preliminary data, only 24 h treatment with CL 2 μg/ml and BS 50 μg/ml or the combination of CL 2 μg/ml and BS 50 μg/ml have been further analyzed.

GSEA analysis on GO terms or wikiPathways for CL or BS is shown in Supplementary File S6, and the KEGG diagram in Supplementary File S7.

### *C. longa* Effect on OA Chondrocyte Transcriptome

The most up-regulated genes with CL (HMOX1, NQO1, SQSTM1, FTH1, GCLM, TNC, TXNRD1, EPHX1, etc.) were anti-oxidant and cytoprotectives genes belonging to the *Nrf2* pathways (Supplementary File S8).

CL down-regulated 110 pro-inflammatory genes in GO ontology « inflammatory response GO:0006954 » (Supplementary File S8), including NFKB1, NFKB2, chemokines (CXCL6, CXCL1, CXCL8/IL-8, CCL2, etc.), cytokines (including TNFα, IL6, IL16, IL17R, and IL34), NOS2, and other pro-inflammatory genes (including FSTL1, POSTN, IGFBP4, and CHI3L1). CL also modulated the expression of several genes of the arachidonic acid metabolism. It decreased the two most expressed phospholipase A2 genes by human chondrocytes (PLA2G2A and PLA2G4A), but also PTGS1 and PTGS2 (also known as COX-1 and COX-2), as well as PTGES and PTGES2. In contrast, CL increased PTGS3. CL decreased several prostanoid receptors including PGE_2_ (PTGER4 and to a lesser extent PTGER2), PGF_2a_ (PTGFR), and PGI_2_ (PTGIR) receptors (Supplementary File S4 and KEGG diagram in Supplementary File S7). CL had also important inhibitory effects on genes involved in cartilage catabolism like MMP13, MMP1, ADAMTS5, MMP3, and HTRA1. It highly up-regulated HTRA3 (5-fold increased) and down-regulated 126 genes involved in cartilage development and endochondral ossification (ACAN, COL2A1, COMP, TGM2, F13A1, IBSP, CILP, SPARC, CCN2, GREM1, WNT5A, FGF1, VEGFA, VEGFB, VEGFC, etc.) (Supplementary File S8).

### *B. serrata* Effect on OA Chondrocyte Transcriptome

**FIGURE 2:**
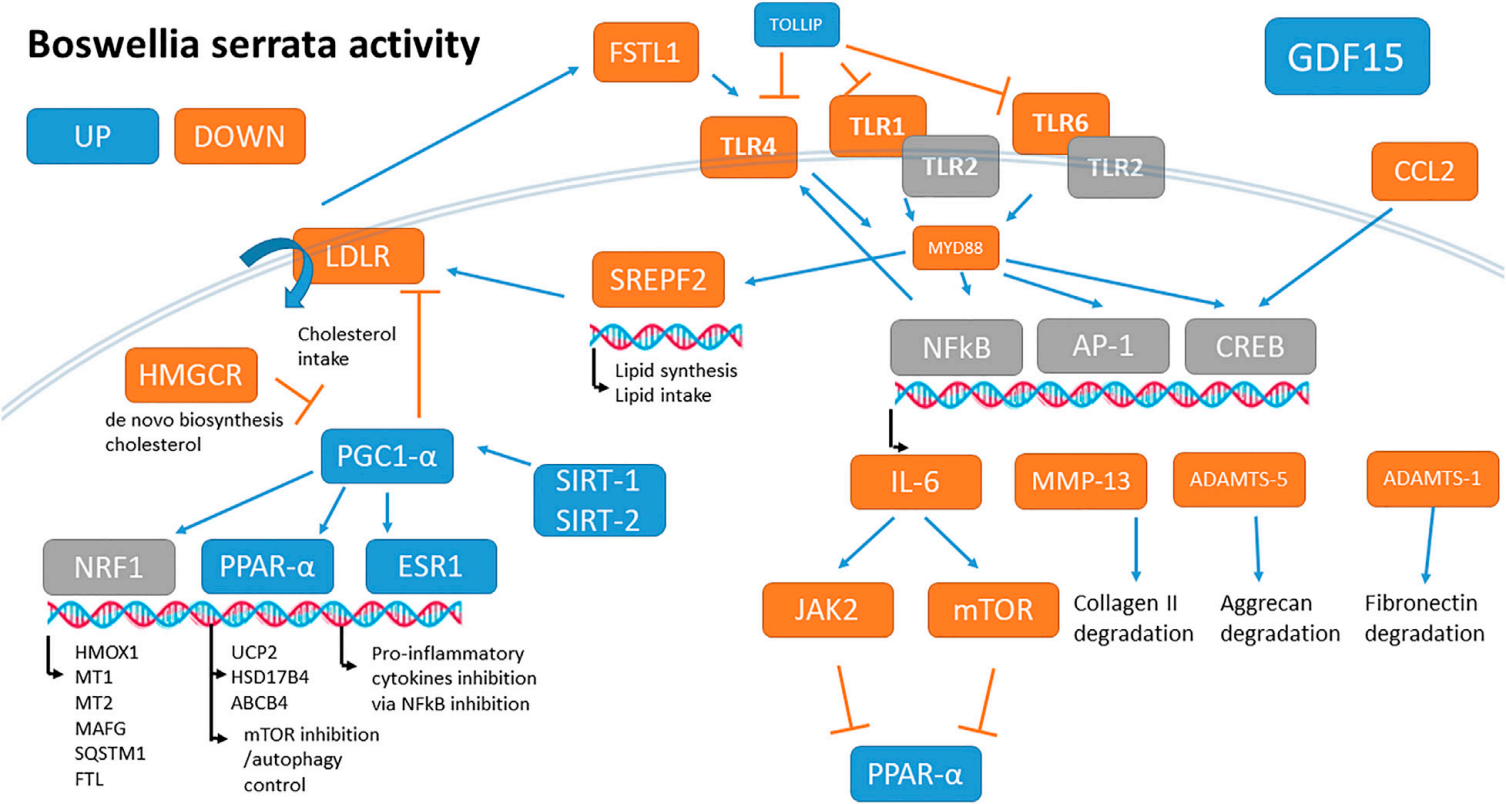
Summary of main *B. serrata* activities on OA chondrocytes after 24 h of treatment. Genes in blue are up-regulated, in orange are down-regulated, and in grey are unchanged.

The most BS up-regulated gene was GDF15 (2.4 log2fold change corresponding to a 5.3-fold increase). Many genes carrying one *anti-oxidant response element* (ARE) were also up-regulated by BS, such as HMOX1 and FTL, and metallothioneins which are a signature of *Nrf1* activation (Supplementary File S8 and Figure 2). PPAR-α (PPARA, +12%) and PPAR-γ coactivator-1 alpha (PPARGC1A+46%, also known as PGC-1α), SIRT1, and SIRT2 were also up-regulated.

Many genes are involved in lipid cell intake and cholesterol biosynthesis, including the LDL receptor (LDLR), HMG-CoA reductase (HMGCR), HSD17B7, FDFT1, SQLE, LSS, CYP51A1, DHCR7, DHCR24, and SREBF2 were significantly down-regulated by BS.

BS regulated a lot of genes involved in inflammation. IL6, CCL2, FSTL1, IL16, IL17 receptors, MyD88, TLR1, TLR4, and TLR6 were down-regulated while HMGB2, NRROS, CYP26B1, and TOLLIP were up-regulated (*See* Supplementary File S9 for details). Note that both IL36 and its antagonist IL36RN were up-regulated.

BS significantly up-regulated the autophagy pathways (GO BP 0006914 and related). Autophagy inducer genes like FOXO1, LAMP2, RUBCNL, SQSTM1, and PLEKHM1 were up-regulated (Supplementary File S9 and Table 5).

Finally, BS down-regulated some important catabolic enzymes like ADAMTS1 and 5, MMP3, and MMP13 but also a panel of 57 genes involved in cartilage development and endochondral ossification (including 16 of the 34 collagen genes expressed, and most genes involved in chondrocyte hypertrophy like FGF1, POSTN, COL10A1, ASPN, IBSP, and BMP5) (Supplementary File S9). The global *B. serrata’s* main activities are schematized in Figure 2.

### Distinct and Common Pathways by *C. longa* and *B. serrata* Extract

**FIGURE 3:**
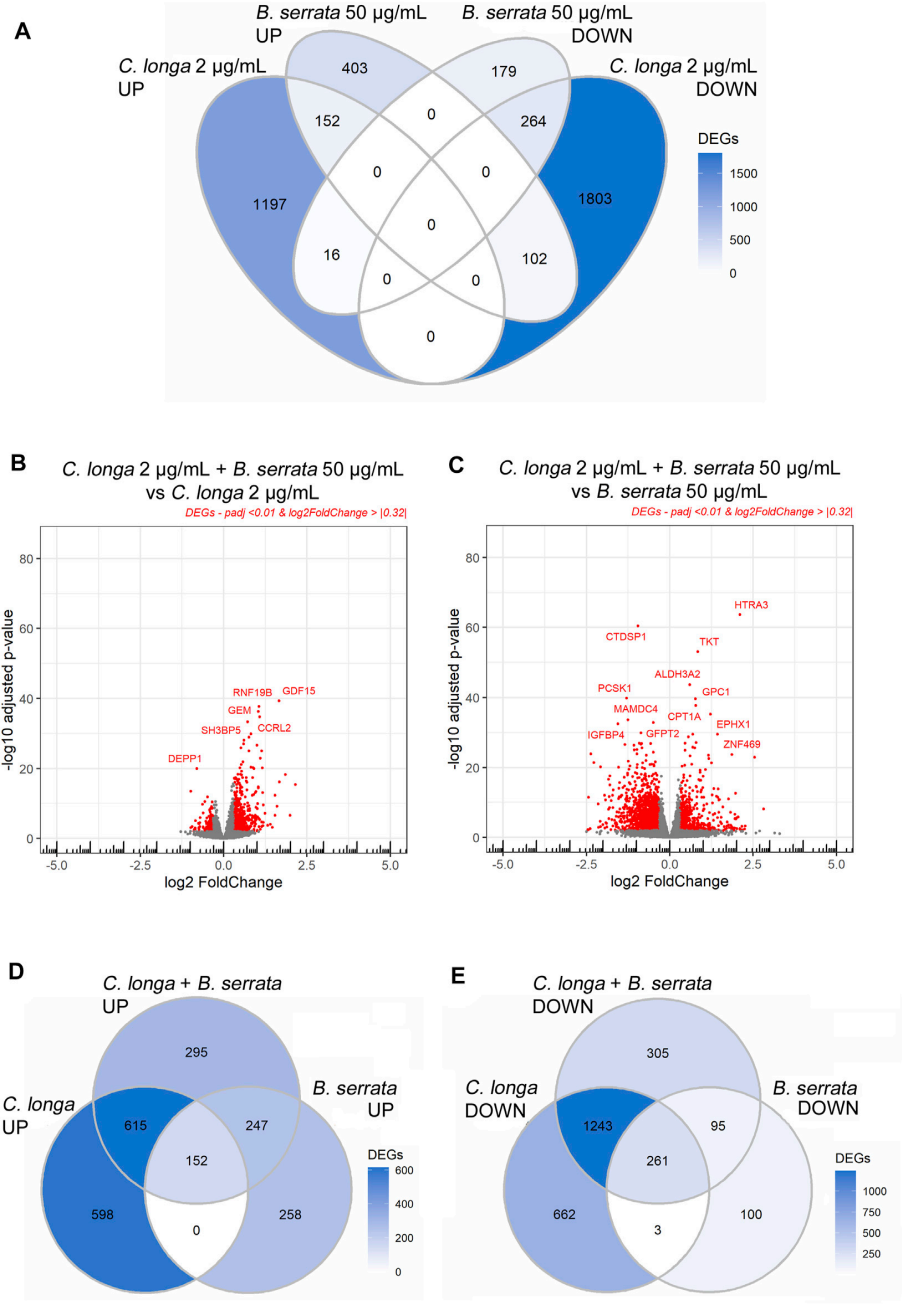
Volcano plots and Venn diagrams of *C. longa* 2 μg/ml, *B. serrata* 50 μg/ml, and the combination after 24 h treatment on OA chondrocytes. Threshold padj <0.01 and Log2FoldChange >|0.32|, *n* = 10.

From the 3534 DEGs by CL 2 μg/ml, 534 were also modified by 50 μg/ml BS. From these, 152 up-regulated and 264 down-regulated were common, and 118 were regulated in an opposite way (Figure 3).

The CL and BS DEGs were individually analyzed to identify those which were preferentially regulated by CL 2 μg/ml or BS 50 μg/ml. Using ANOVA and Bonferroni post-test, we compared gene by gene, and for each patient, the log2FC. We found that 591 genes were similarly modified by the two extracts (“similar”), 104 genes were significantly more affected by BS than CL (“BS50 driven”), and 377 genes were more regulated by CL than BS treatment (“CL2 driven,” Supplementary File S9). When comparing the DESeq2 between the combination CL2/BS50 with those of CL2 or BS50, we showed that the combination was statistically more efficient (*p* < 0.05) on 258 genes than CL 2 μg/ml alone, 629 genes than BS 50 μg/ml alone, and 125 genes than both CL 2 μg/ml and BS 50 μg/ml (Supplementary File S10).

**FIGURE 4:**
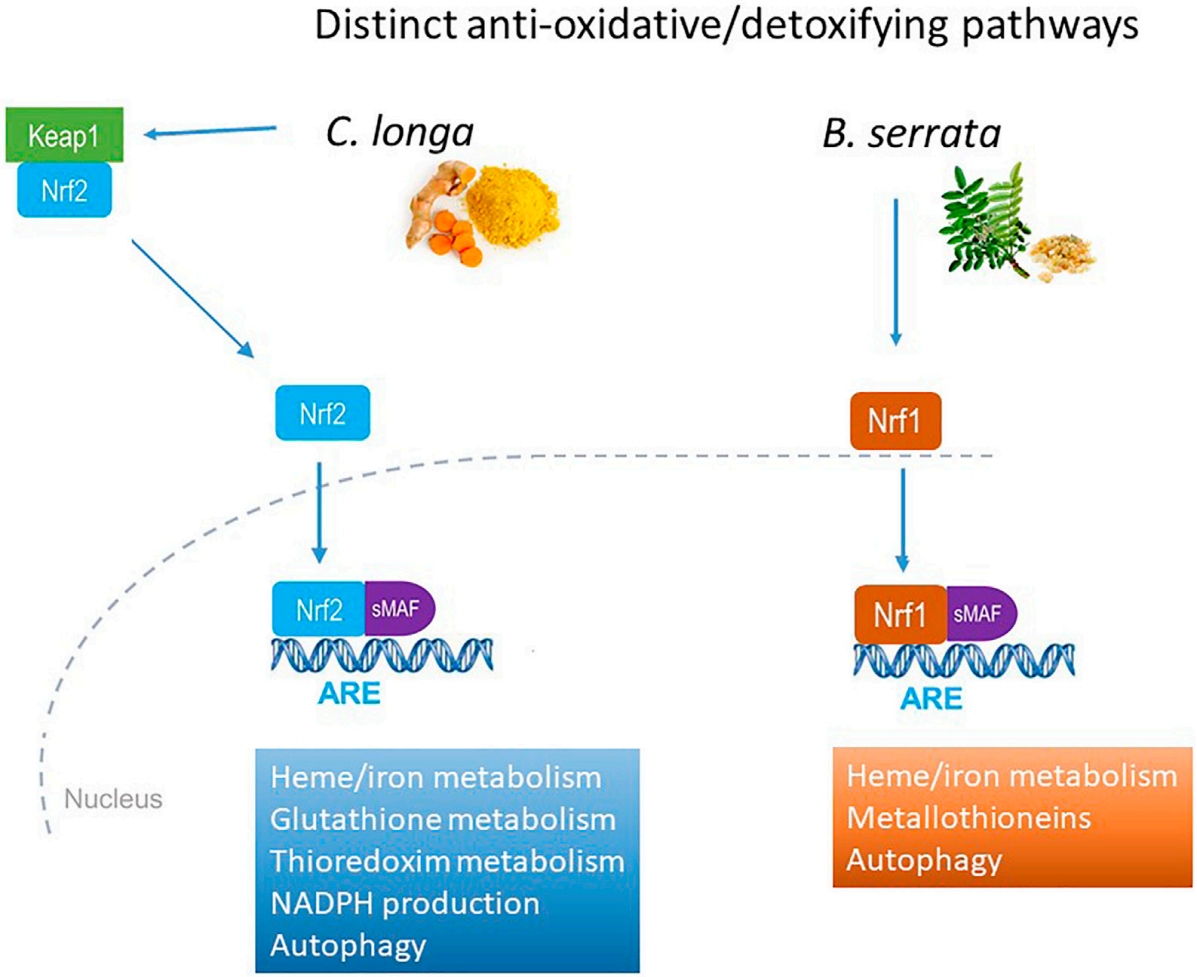
Distinct anti-oxidative and detoxifying pathways activation by *C. longa* and *B. serrata* on OA chondrocytes.

Inversely, the two extracts were antagonists of some transcription factors involved in Nrf pathways. *C. longa* activated *Nrf2* and *B. serrata Nrf1* pathway, both involving common small MAFs (Figure 4).

The combination of CL with BS did not modify the effect of CL on genes related to *Nrf2*. Metallothioneins which are *Nrf1*-specific genes were strongly down-regulated by CL (−0.35 to −1.46 log2FC) but up-regulated in the presence of BS. MT1X was the only metallothionein gene up-regulated by BS in the presence of CL but was less differentially expressed by chondrocytes in the presence of CL (0.69 vs. 1.21 log2FC with BS alone). The up-regulation of BS on PPARGC1A fully disappeared in the presence of CL.

Tables 1, 2 summarize the comparative anti-inflammatory and anti-catabolic activities of CL and BS, highlighting some common and specific targets for the two extracts.

**TABLE 1:** Summary of the anti-inflammatory main mechanisms of action of *C. longa* (2 μg/ml) and *B. serrata* (50 μg/ml) extract at 24 h.

Pathways	*C. longa* only	*B. serrata* only	Common
Cytokines	CSF1, IL32, IL34, TNF		**IL6**, IL16, TNFSF10
Chemokines	CCL3, CCL4, CCL20, CXCL1, CXCL2, CXCL5, CXCL6, CXCL8, CXCL10, CX3CL1		**CCL2**, CCL7, CXCL3
Cytokine/chemokine receptors/transducers	TNFRSF1B, CSF1R	IL10RA	IL17RB, IL17RC, IL17RE JAK2
TLRs	**TLR2**	**TLR1, TLR4, TLR6**, MyD88, TOLLIP	
Arachidonic acid metabolism	PLA2G4A, PTGS1, PTGS2, PTGES, PTGES2, PTGIR, PTGER2, PTGFR		PLA2G2A, PTGER4
Others	S100A9		CHI3L1, CHI3L2, **GDF15**, FSTL1, IGFBP4, POSTN, **NO**_**2**_

Genes in bold are those confirmed using proteomic methods in this study. *C. longa* decreased both IL1R agonists and antagonists, and both *C. longa* and *B. serrata* act on agonists and antagonists of IL36R probably resulting in no effect, thus these cytokines are not represented in this table.

**TABLE 2:** Summary of the anti-catabolism main mechanisms of action of *C. longa* (2 μg/ml) and *B. serrata* (50 μg/ml) extract at 24 h.

Pathways	*C. longa* only	*B. serrata* only	Common
ADAMTSs	ADAMTS4, ADAMTS9		ADAMTS1, ADAMTS5
MMPs	MMP1, MMP2, MMP10, MMP14		MMP3, MMP13
Others	HTRA1		

### Proteomic Analysis

**FIGURE 5:**
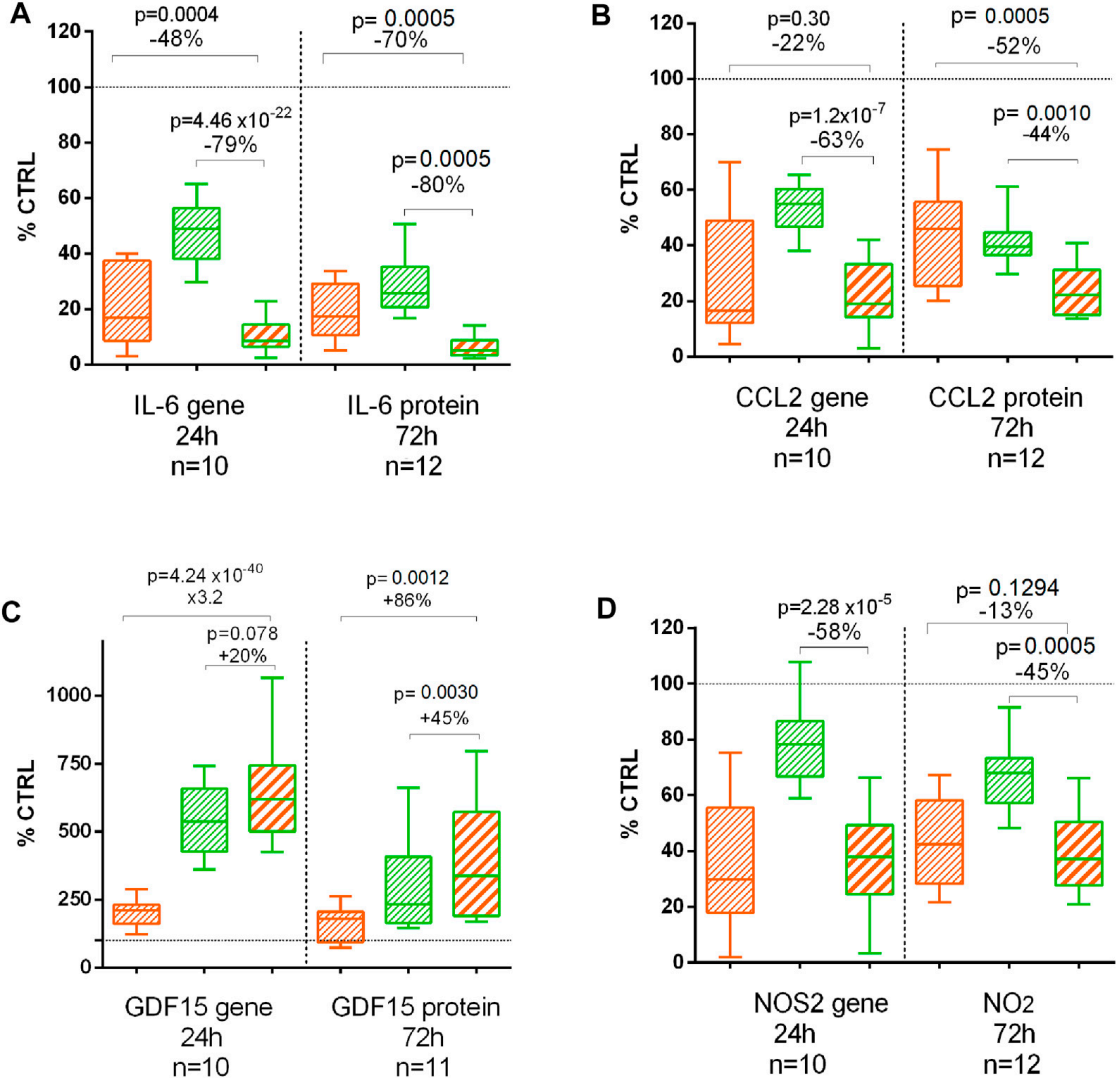
Effect of *C. longa* 2 μg/ml (orange), *B. serrata* 50 μg/ml (green), and combination of *C. longa* and *B. serrata* on IL6 **(A)**, CCL2 **(B)**, GDF15 **(C)**, and NOS2/NO_2_ **(D)** gene expression (RNA-seq at 24 h) and protein production (72 h).

IL-6, CCL2, GDF15, and NO_2_ were quantified on 72 h treated chondrocyte supernatants (Figure 5). As for the gene expression at 24 h, CL and BS significantly decreased IL-6, CCL2, and NO_2_ but increased GDF15 protein release after 72 h. The combination was significantly more effective than CL or BS alone on all these proteins after 72 h, except no difference between CL and the combination for NO_2_ production (Figure 5).

**FIGURE 6:**
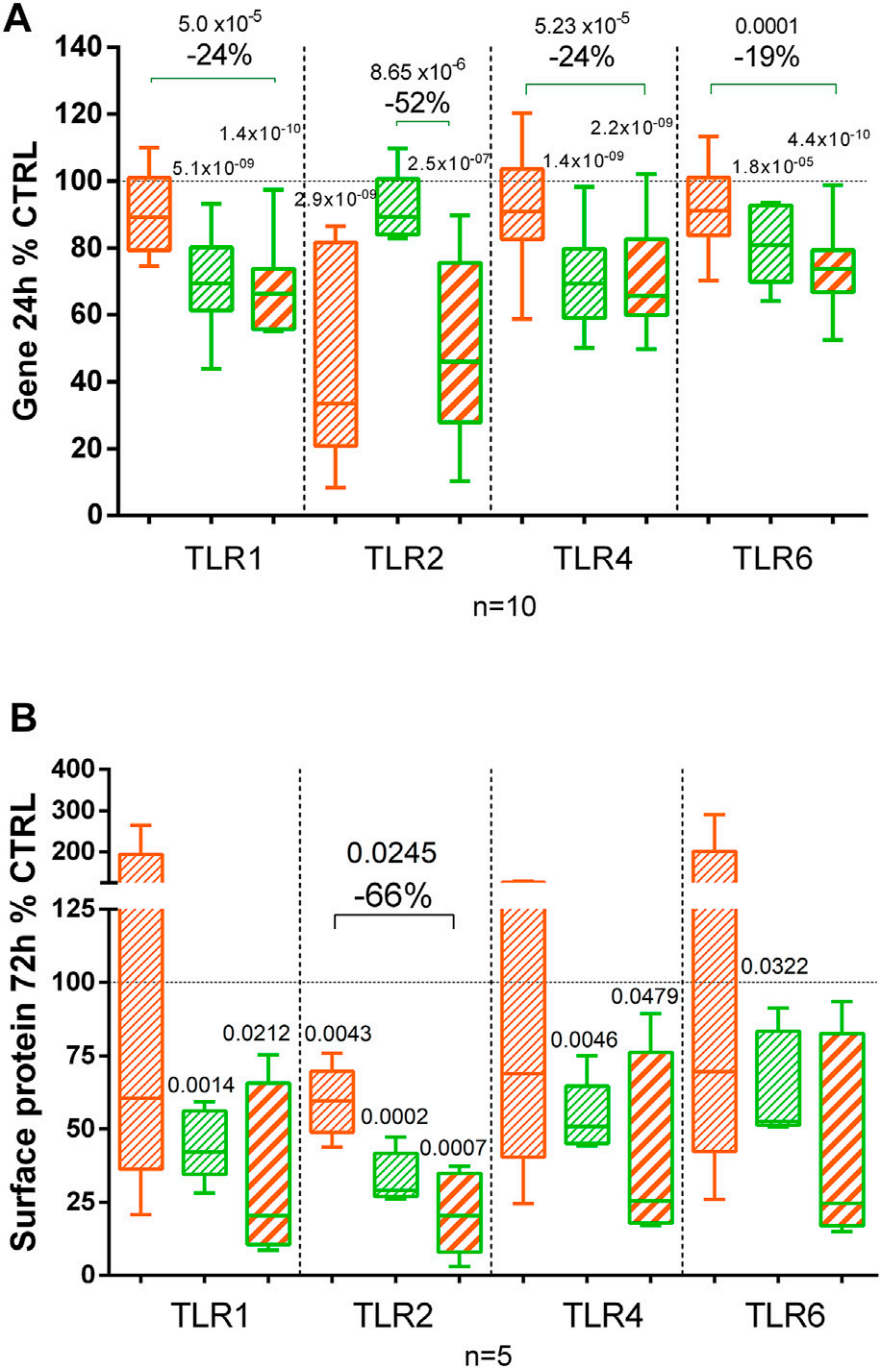
Effect of *C. longa* 2 μg/ml, *B. serrata* 50 μg/ml, and combination of *C. longa* and *B. serrata* on TLRs gene expression (**A**, RNA-seq at 24 h, *n* = 10) and surface protein (**B**, 72 h, *n* = 5). Significant statistical differences are shown compared to the control or between treatments (shown with a line).

Using flow cytometry, we compared the presence of the TLR1, TLR2, TLR4, and TLR6 at the chondrocyte surface after 72 h of treatment (Figure 6). CL decreased significantly TLR2 protein but did not affect TLR1, TLR4, and TLR6. BS significantly decreased all tested TLR receptors at the chondrocyte surface (Figure 6). No statistical difference was observed among BS, CL, and the combination of both extracts.

## Discussion

In this study, we aimed to identify molecular pathways modulated by *C. longa* and/or *B. serrata* extracts in human OA chondrocytes. We used NGS technology to generate the transcriptome, capturing dynamic changes in chondrocytes induced by the treatment, compared to control or each extract independently. For the most regulated genes, we extended our transcript analysis to the protein level by directly measuring proteins in culture supernatants with immunoassays or flow cytometry. We demonstrated that *C. longa* and *B. serrata* had different time-course effects on OA chondrocyte genes and acted on different but complementary pathways to induce anti-oxidative, detoxifying, anti-inflammatory, and anti-catabolism activities. These findings may explain some effects observed in the clinical studies with OA patients and justify the combined use of CL and BS (Sengupta et al., 2008; Haroyan et al., 2018; Henrotin et al., 2019; Wang et al., 2020) (Bannuru et al., 2018; Zeng et al., 2021).

It is well known that curcumin activates the *Nrf2* pathways, and particularly induces HMOX1 gene expression (Balogun et al., 2003). More recently, it was demonstrated that curcumin interacts directly with Keap1 to activate *Nrf2* pathways and induce anti-oxidant and cytoprotective genes expression (Rahban et al., 2020). In this study, we showed that in chondrocytes, *C. longa* induced transcription of genes involved in iron/heme homeostasis, detoxification of ROS (glutathione and thioredoxin metabolism), autophagy, and enhanced the redox potential *via* NADPH. *B. serrata* induced the same iron/heme homeostasis and autophagy genes but in addition induced metallothioneins, a family of cysteine-rich metal-binding proteins that are important for zinc and copper homeostasis, protection against oxidative stress, and buffering against toxic heavy metals.

*C. longa*-specific effects concern TNF inhibition, arachidonic acid metabolism to decreased prostanoids production and their receptors, and decrease in VEGFs and HTRA1 genes. In parallel to the decrease in TNF alpha gene expression, *C. longa* also decreased the expression of several TNF-induced protein genes (TNFAIP2, 3, 6, and 8, Supplementary File S4). These TNFAIPs have a positive role in the resolution of inflammation (Chen et al., 2020). The role of HTRA1, a serine protease, in joints has been recently reviewed (Tossetta et al., 2022). HTRA1 is involved in aggrecan, type II, VI collagens, and other matrix protein degradation as fibronectin (Chen et al., 2019). Its expression level is correlated with MMP13 and is increased in OA patients (Li et al., 2018)**.** This is the first time that the inhibition of HTRA1 synthesis by CL is demonstrated. This new mechanism of action of CL supports clinical data demonstrating that CL reduced cartilage catabolism in OA (Henrotin et al., 2019).

**FIGURE 7:**
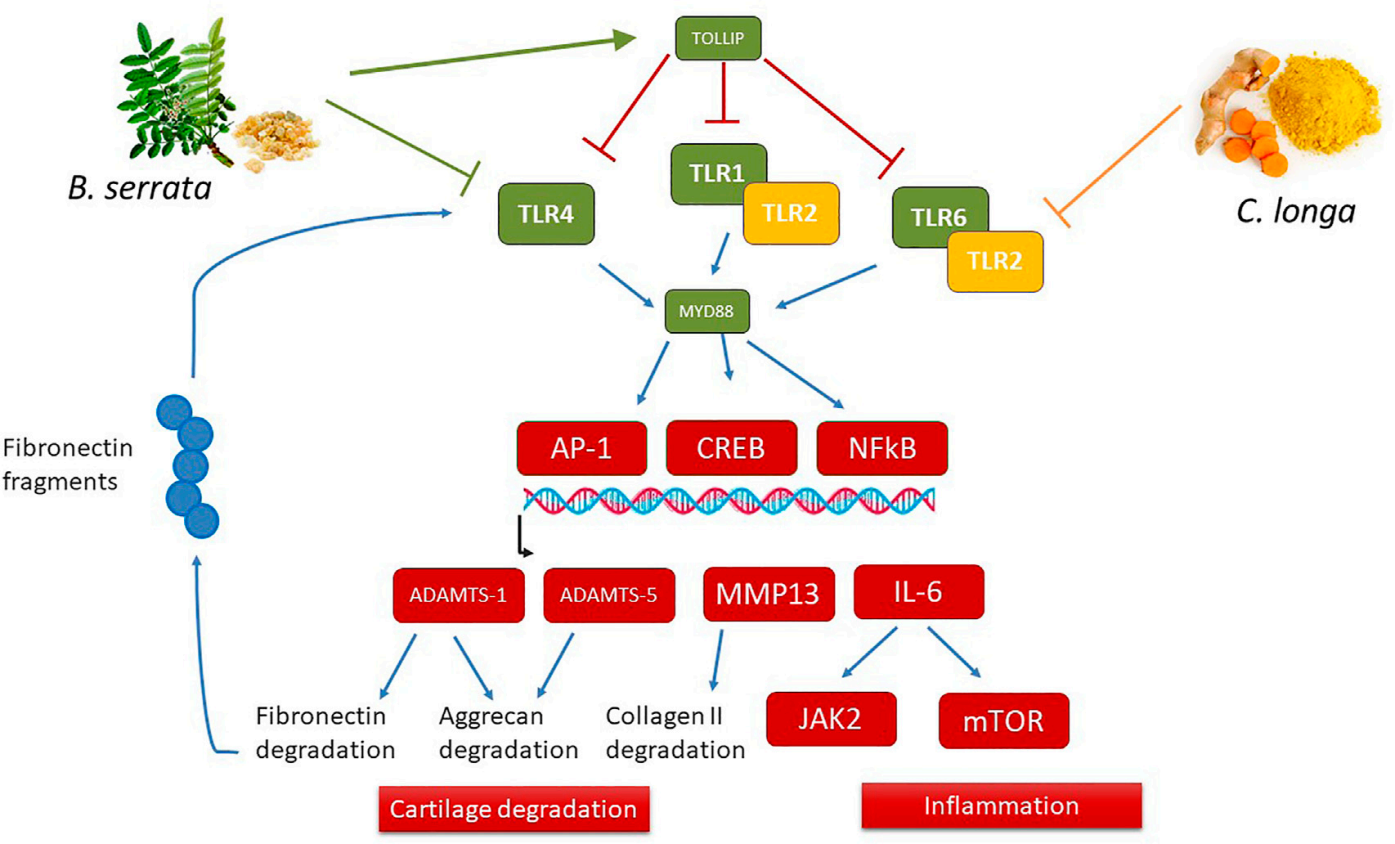
Schematic overview of distinct action of *C. longa* and *B. serrata* on TLRs pathways. *B. serrata* decrease TLR1-4-6 and MyD88 gene expression after 24 h and TLR2 protein after 72 h, and also up-regulated TOLLIP gene expression, an inhibitor of TLRs, while *C. longa* only decreases TLR2 expression and protein. Both *C. longa* and *B. serrata* decreased downstream genes of the TLRs pathways like ADAMTS1, ADAMTS5, MMP-13, IL-6, and JAK2. As ADAMTS1 can degrade fibronectin, and fibronectin fragments could induce TLR4 activation, *C. longa* and *B. serrata* could attenuate this inflammation amplification loop.

The most particular effect of *B. serrata* was the inhibition of the inducible TLRs pathways, the fatty acid intake, and the intracellular cholesterol metabolism (HMG-CoA reductase-HMGCR and other enzymes involved in cholesterol synthesis). The action of BS on TLR is important since it was demonstrated that DAMPS released during cartilage extracellular matrix degradation may stimulate chondrocytes to secrete more catabolic and inflammatory mediators by binding to TLR receptors (Lambert et al., 2020). BS reduced SREBF2 expression, a transcription factor induced by TLR4 and which induces LDLR and HMGCR expression (Dong et al., 2021). *B. serrata* increased PPARGC1A expression which is decreased in chondrocytes during OA and seems to be a key factor needed for adequate mitochondrial function and pro-inflammatory response to IL-1β (Wang et al., 2015). Therefore, reducing the TLR receptors is now presented as a therapeutic strategy to prevent cartilage degradation. All these pathways interact to control chondrolysis as illustrated in Figure 7. PPARGC1A expression is controlled by several signaling pathways including SIRT1, which is also up-regulated by *B. serrata* **.** Furthermore, PPARGC1A/NRF1 induces telomers transcription, providing anti-senescence activity (Diman et al., 2016). *B. serrata* also induced HMGB2, a gene specifically inhibited in senescent cells (Guerrero and Gil 2016; Kim et al., 2018; Lawrence et al., 2018). This anti-senescence could contribute to prevent cartilage degradation in OA. Indeed, the chondrocyte senescence process was demonstrated to be an important feature in OA, related to the autophagy, oxidative, and inflammatory state of the cells (Rim et al., 2020).

With aging, the basic autophagic activity of cells decreases, following a down-regulated clearance efficiency. Subsequently, the aggregation of various macromolecular proteins increases, leading to eventual cell degeneration and functional defect, or even apoptosis (Duan et al., 2020). Recent studies have shown that the level of autophagy in OA cartilage is reduced (Feng et al., 2020), and that autophagy can protect chondrocytes from degradation (Carames et al., 2012). BS induces several genes promoting autophagy (FOXO1, RUBCNL, PLEKHM1, and SQSTM1), autophagy markers like ATG2A, and genes improving lysosomal functions like LAMP2, all of which would be beneficial in OA context (Kurakazu et al., 2019; Zhao et al., 2019; Ansari et al., 2021; Feng et al., 2022).

In this work, we attempted to use concentrations close to the plasmatic concentration achieved during oral treatment with *C. longa* and *B. serrata* extracts. But, due to the low bioavailability of curcumin, the efficacity could be greatly affected. This is for which *C. longa* extract needs to be optimized to increase curcumin bioavailability (Henrotin et al., 2013). However, most *in vitro* studies on curcumin effects use higher curcumin concentrations, usually ranging from 10 to 20 µM, even 50 µM. We observed that in addition to being unreachable in the clinic, curcumin concentrations of 10 µM and above are decreasing chondrocyte cell viability (personal communication). Concerning *B. serrata* extract, the extrapolation is further complicated by the different bioavailability of the six main boswellic acids supposed to be active. The more studied boswellic acid, acetyl 11-keto-β-boswellic acid, is less bioavailable (0.1 µM compared to 2.6 µM in the 50 μg/ml extract used) but others, also known to be very active like β-boswellic acid, are under-represented in our study (up to 30 µM in the plasma compared to 17 µM in the 50 μg/ml extract) (Buchele and Simmet 2003; Husch et al., 2013). Globally, we can estimate that our 10 to 50 μg/ml extract represents well the archived range of plasmatic concentrations during the oral treatment.

The main limitation of this study is the cartilage source. The cartilage came from patients with severe knee OA requiring joint replacement. In these patients, the cartilage was very damaged which did not represent all the osteoarthritis chondrocyte endotypes. Furthermore, a low number of OA cartilage samples were included in the flow cytometry experiment (*n* = 5). A larger number would maybe provide better statistical differences between the treatments.

**FIGURE 8:**
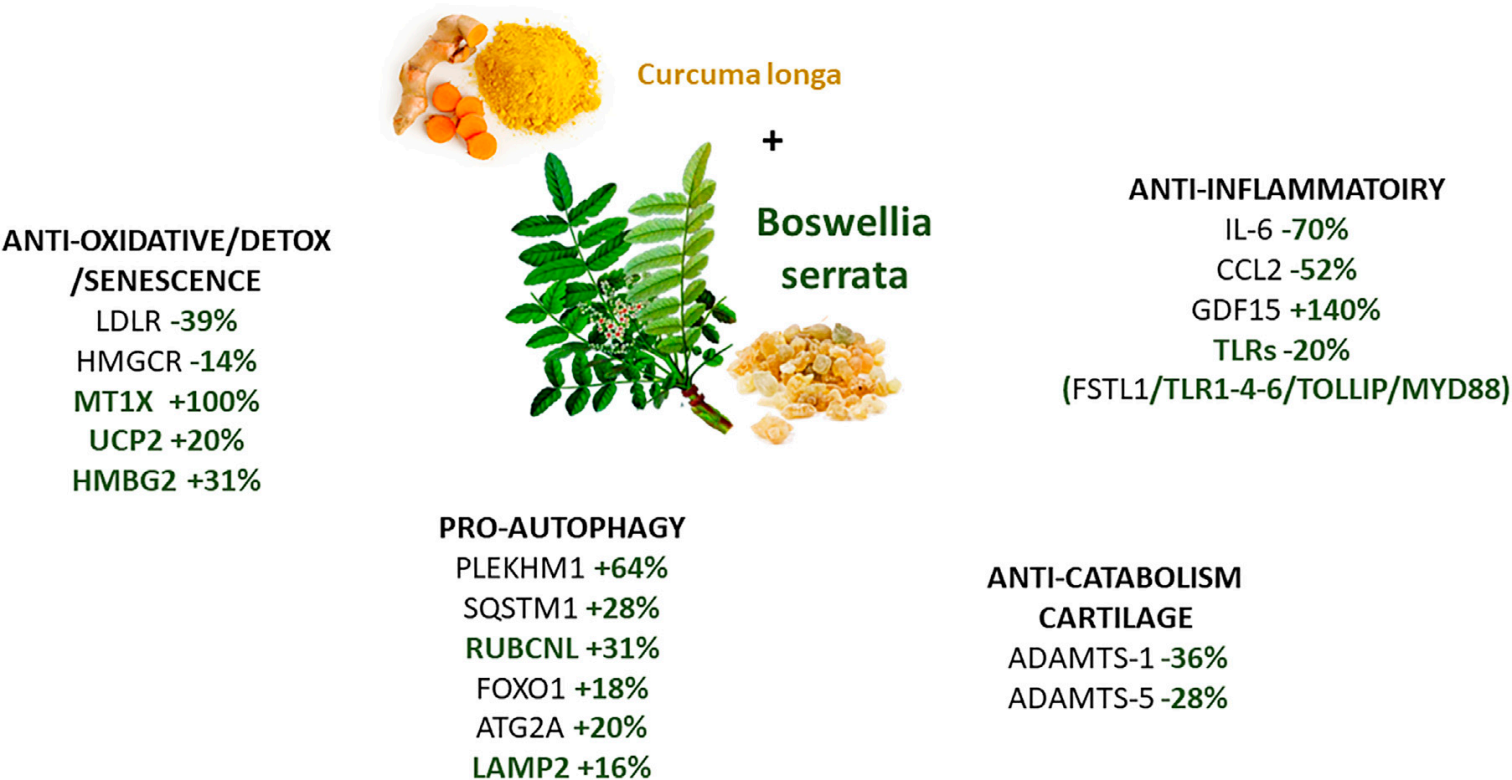
Benefits of using *B. serrata* in combination with *C. longa* for treating OA. The percentage is shown in comparison to *C. longa* alone. Genes in green and bold are DEGs specific to *B. serrata* treatment and unmodified with *C. longa* treatment.

In conclusion, in primary OA chondrocyte cultures, *C. longa* and *B. serrata* exert distinct and complementary anti-oxidative, anti-inflammatory, and anti-catabolic activities on OA articular chondrocytes (Figure 8). This *in vitro* study gives a rationale for their combined use in the therapeutic of OA.

## Data Availability

The transcriptomic data analyzed for this study are deposited in Gene Expression Omnibus (GEO) repository, under accession number GSE201779 that are publicly accessible at https://www.ncbi.nlm.nih.gov/geo/query/acc.cgi?&acc=GSE201779. Other datasets presented in this study are included in the Supplementary Material.

## Abbreviations

ADAMTS, a disintegrin and metalloproteinase with thrombospondin motifs; DMEM, Dulbecco’s modified Eagle’s medium; FBS, fetal bovine serum; FDR, false discovery rate; HEPES, 4-(2-hydroxyethyl)-1-piperazineethanesulfonic acid; IL, interleukin; MMP, matrix metalloproteinase; NGS, next-generation sequencing; OA, osteoarthritis; padj, p-value adjusted; RIN, RNA integrity number; RNA, ribonucleic acid; ROS, reactive oxygen species; SD, standard deviation; TKR, total knee replacement; TLR, Toll-like receptor; TNF, tumor necrosis factor.
