# Supplementary material for: Curcuma longa and Boswellia serrata Extracts Modulate Different and Complementary Pathways on Human Chondrocytes In Vitro: Deciphering of a Transcriptomic Study
Source: Front Pharmacol. 2022 Aug 11;13:931914. doi: 10.3389/fphar.2022.931914 (PMC9403192; doi:10.3389/fphar.2022.931914)
Supplement: Supplementary file 1 [file DataSheet7.PDF]

KEGG diagrams

C. longa 2 µg/mL or *B. serrata* 50 µg/mL

24h on chondrocytes

DEGs padj <0.01

2. hsa00190 oxidative phosphorylation
3. hsa00590 arachidonic metabolism
4. hsa03010 ribosome
5. hsa03013 nucleocytoplasmic transport
6. hsa03050 proteasome
7. hsa04060 cytokine-cytokine receptor interactions
8. hsa04064 NfkB signaling pathway
9. hsa04120 Ubiquitin-mediated proteolysis
10. hsa04141 protein processing in endoplasmic reticulum
11. hsa04144 endocytosis
12. hsa04150 mTOR signaling pathway
13. hsa04216 ferroptosis
14. hsa04310 wnt signaling pathway
15. hsa04657 IL17 signaling pathway

hsa00190

CL | BS

C.Longa 2 μg/mL

B.serrata 50 μg/mL

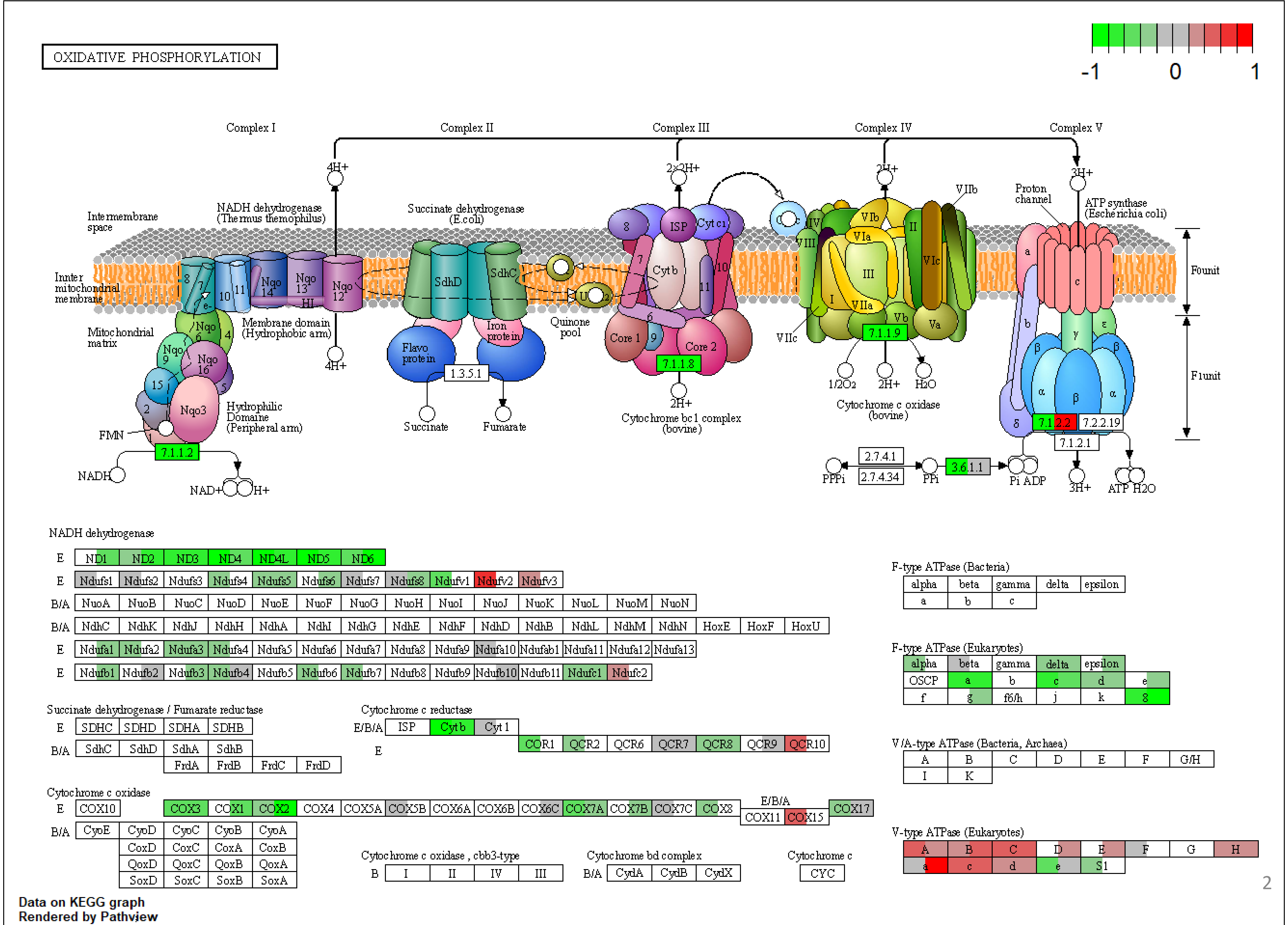

hsa00590

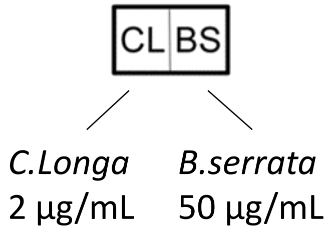

CL BS
C.Longa
2 µg/mL
B.serrata
50 µg/mL

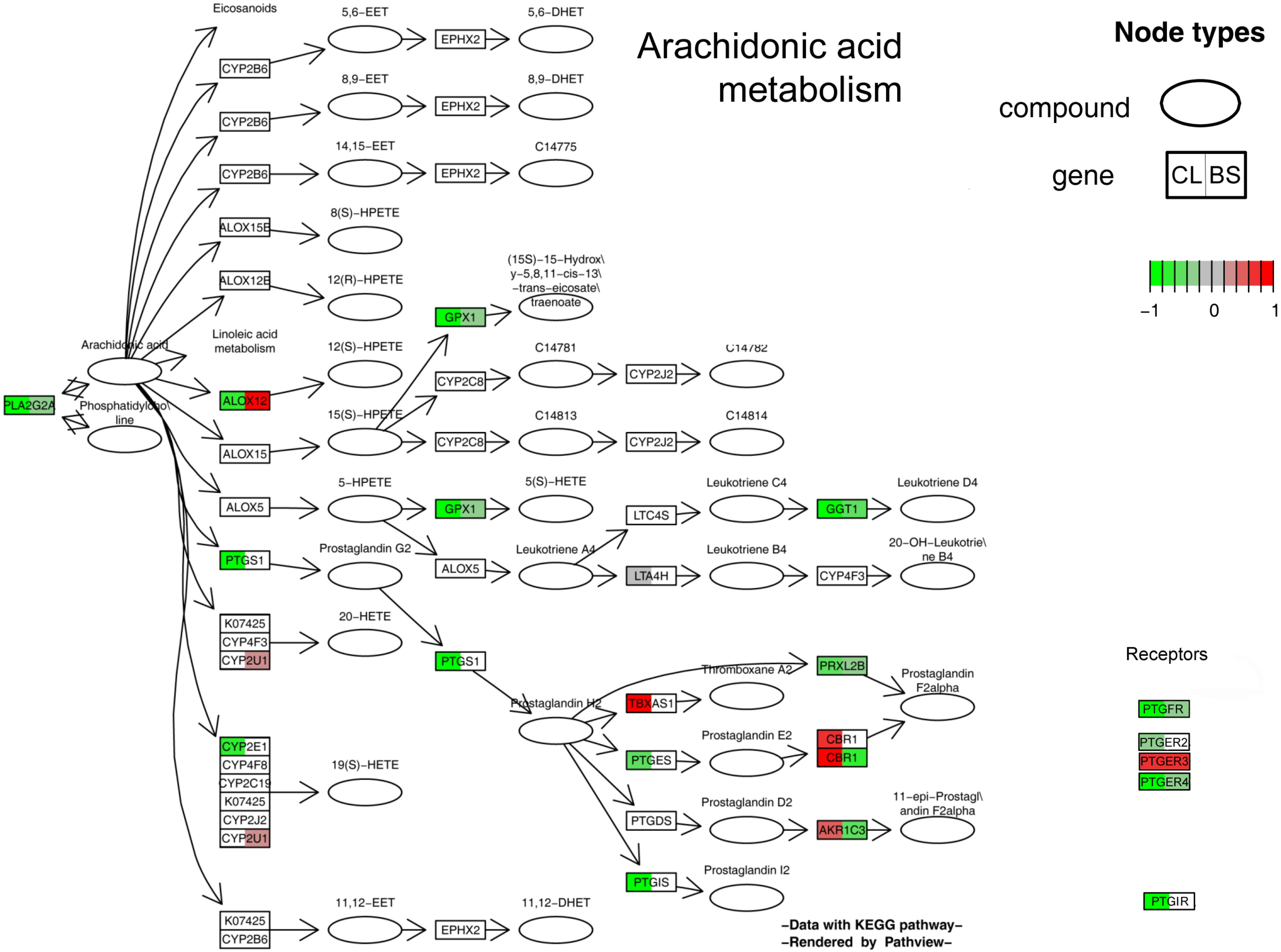

Arachidonic acid metabolism
Node types
compound
gene
CL BS
−1
0
1
Eicosanoids
5,6−EET
5,6−DHET
8,9−EET
8,9−DHET
14,15−EET
C14775
8(S)−HPETE
12(R)−HPETE
(15S)−15−Hydroxy−5,8,11−cis−13−trans−eicosatetraenoate
Linoleic acid metabolism
12(S)−HPETE
C14781
C14782
15(S)−HPETE
C14813
C14814
Arachidonic acid
Phosphatidylcholine
5−HPETE
5(S)−HETE
Leukotriene C4
Leukotriene D4
Prostaglandin G2
Leukotriene A4
Leukotriene B4
20−OH−Leukotriene B4
20−HETE
Thromboxane A2
Prostaglandin F2alpha
Prostaglandin H2
Prostaglandin E2
Prostaglandin D2
11−epi−Prostaglandin F2alpha
Prostaglandin I2
19(S)−HETE
11,12−EET
11,12−DHET
PLA2G2A
CYP2B6
EPHX2
ALOX15B
ALOX12B
GPX1
ALOX12
CYP2C8
CYP2J2
ALOX15
ALOX5
LTC4S
GGT1
PTGS1
LTA4H
CYP4F3
K07425
CYP2U1
PRXL2B
TBXAS1
PTGES
CBR1
PTGDS
AKR1C3
PTGIS
CYP2E1
CYP4F8
CYP2C19
Receptors
PTGFR
PTGER2
PTGER3
PTGER4
PTGIR
−Data with KEGG pathway−
−Rendered by Pathview−

hsa03010

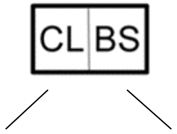

CL
BS
C.Longa
2 µg/mL
B.serrata
50 µg/mL

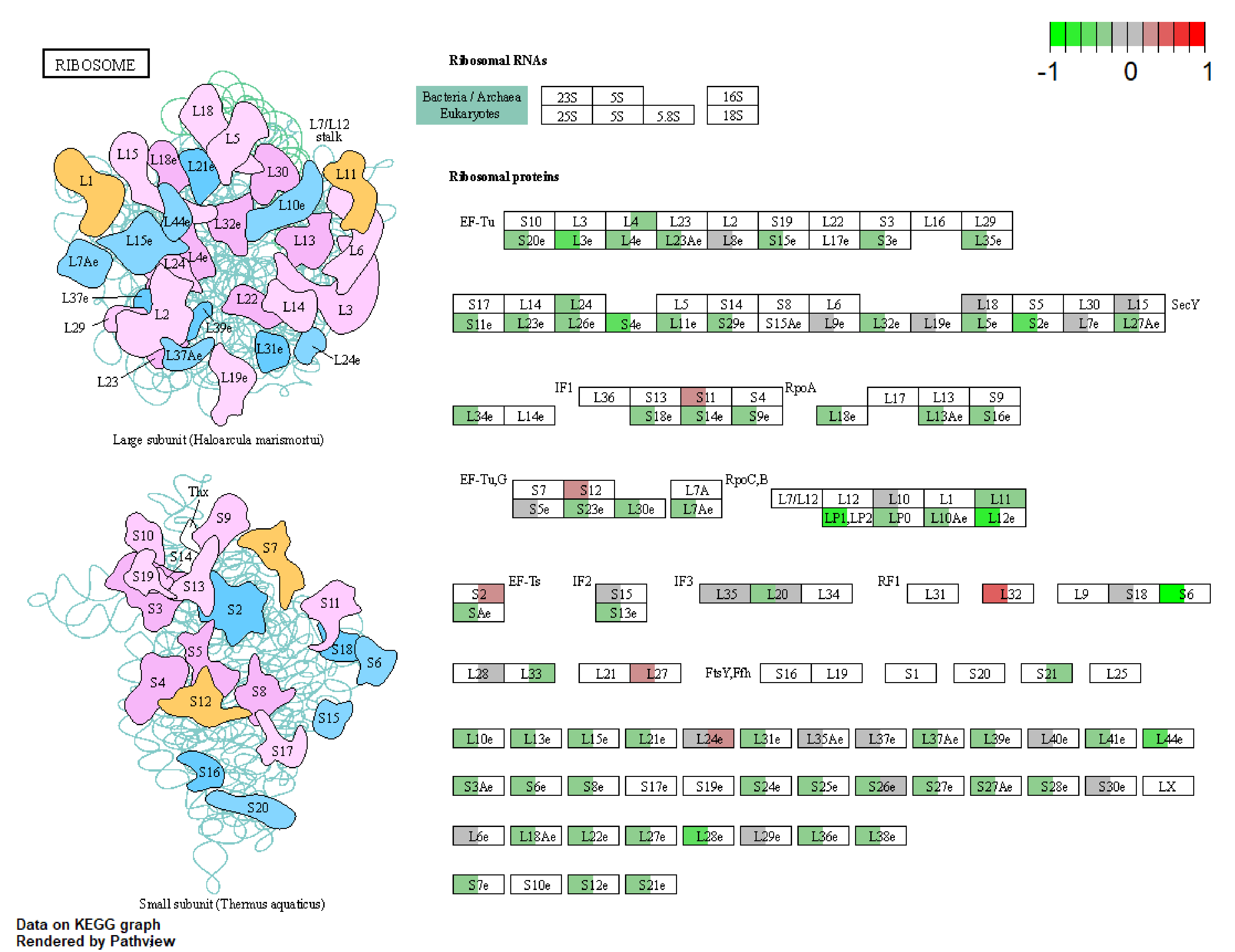

-1
0
1
RIBOSOME
Ribosomal RNAs
Bacteria / Archaea
Eukaryotes
23S
5S
16S
25S
5S
5.8S
18S
Ribosomal proteins
EF-Tu
SecY
IF1
RpoA
EF-Tu,G
RpoC,B
EF-Ts
IF2
IF3
RF1
FtsY,Ffh
Large subunit (Haloarcula marismortui)
Small subunit (Thermus aquaticus)
Data on KEGG graph
Rendered by Pathview

hsa03013

CL | BS

C.Longa 2 µg/mL

B.serrata 50 µg/mL

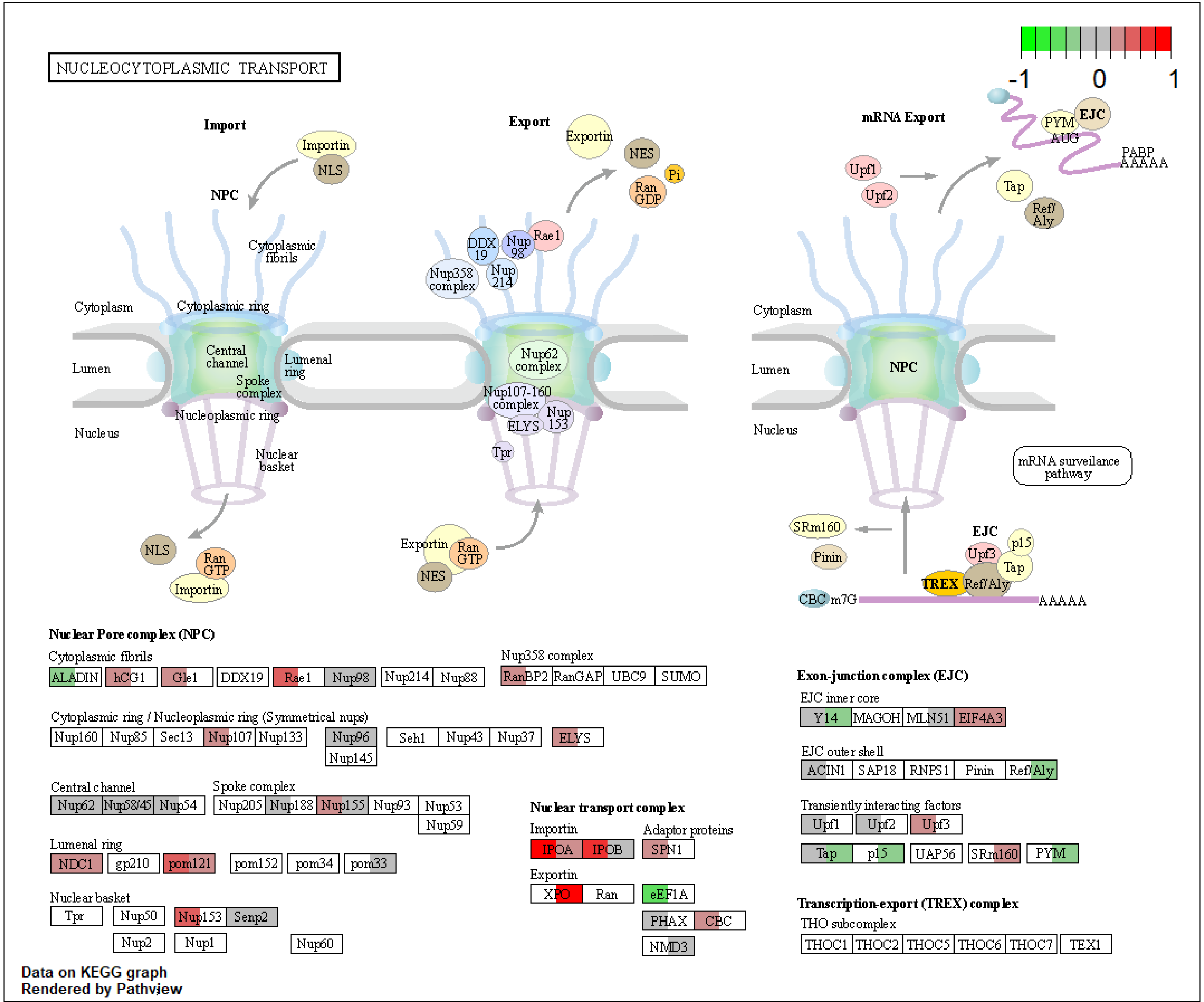

hsa03050

CL	BS

C.Longa 2 µg/mL

B.serrata 50 µg/mL

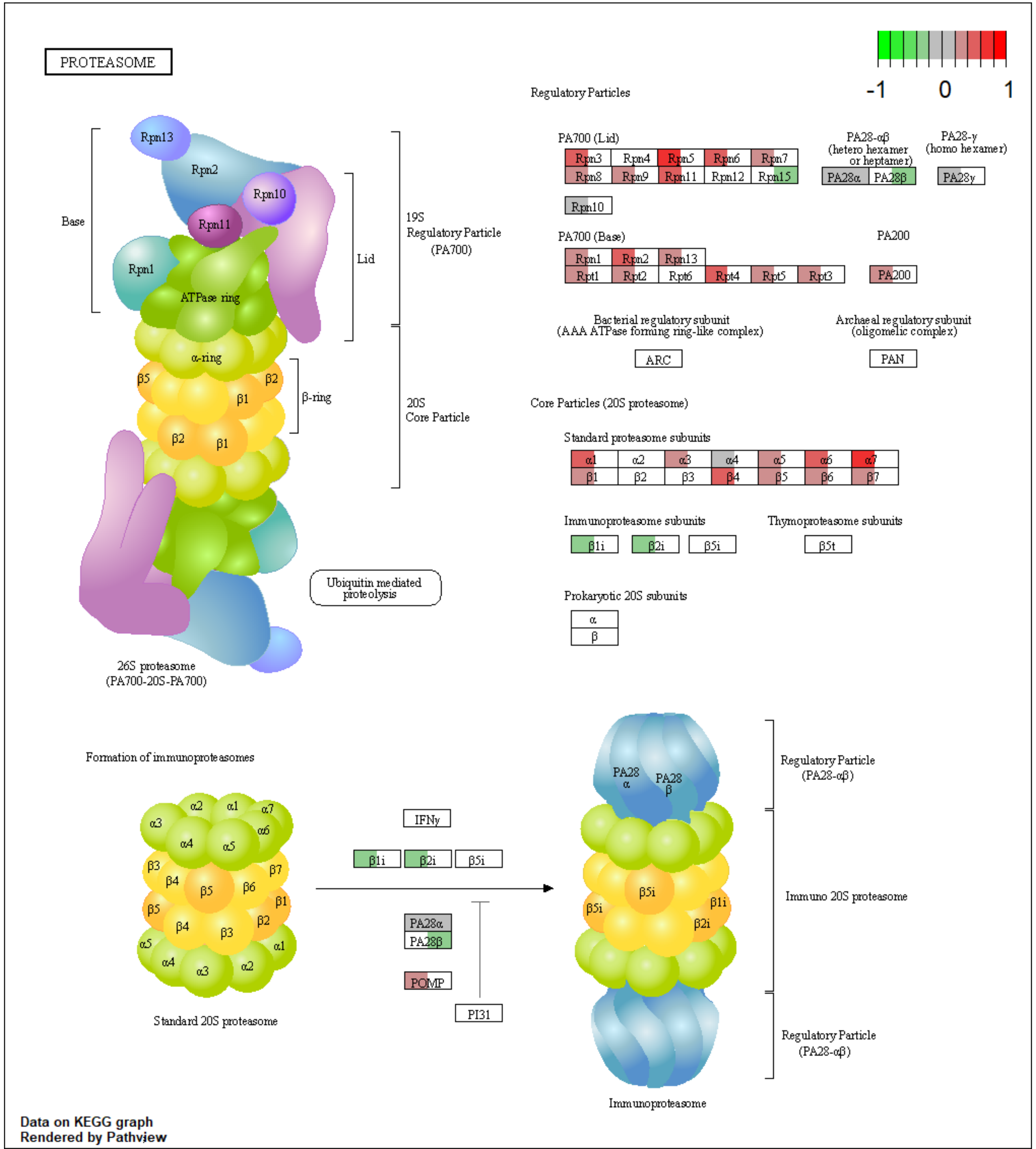

hsa04060

CL | BS

C.Longa 2 µg/mL

B.serrata 50 µg/mL

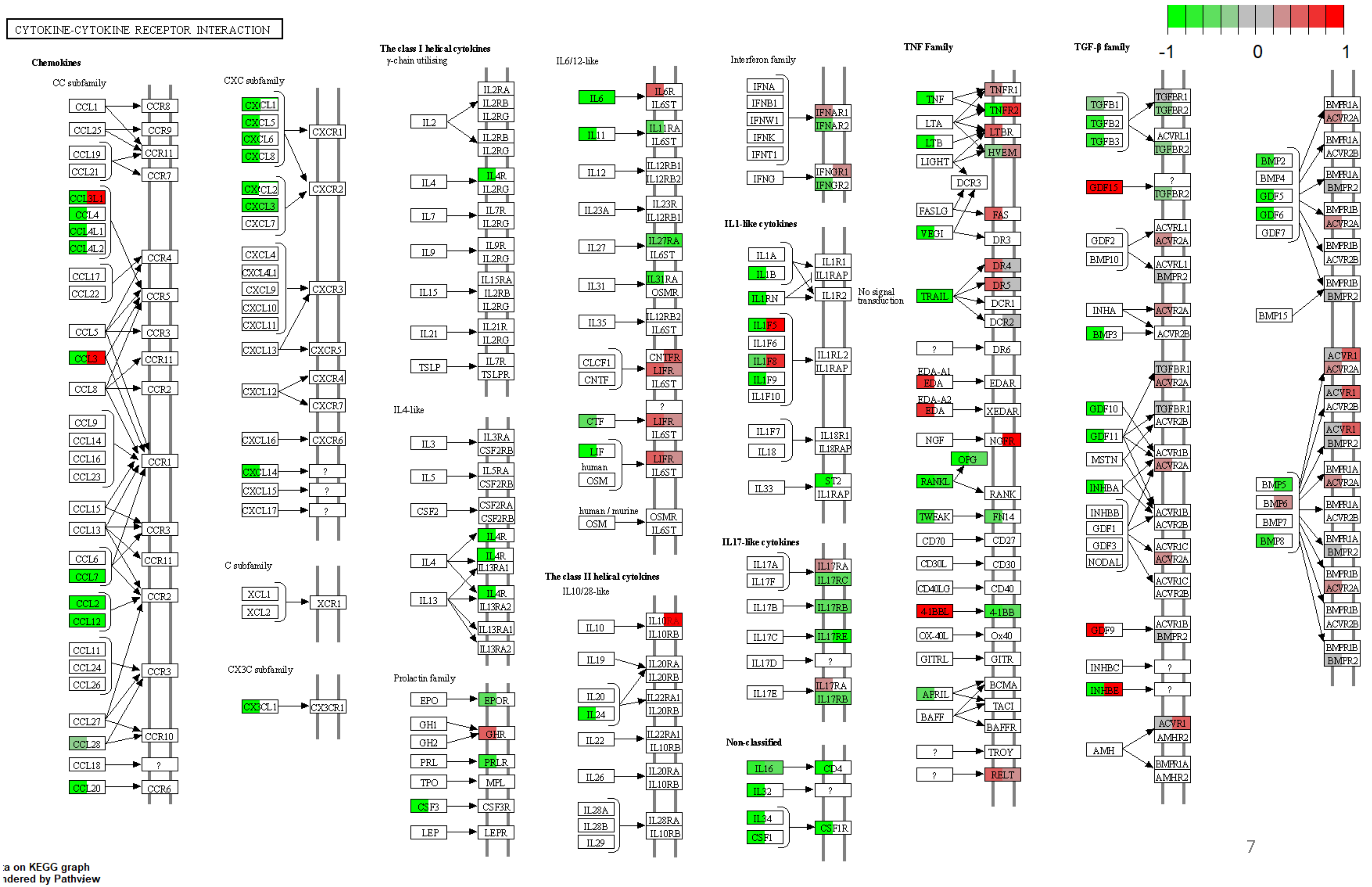

hsa04064

CL | BS

C.Longa 2 µg/mL

B.serrata 50 µg/mL

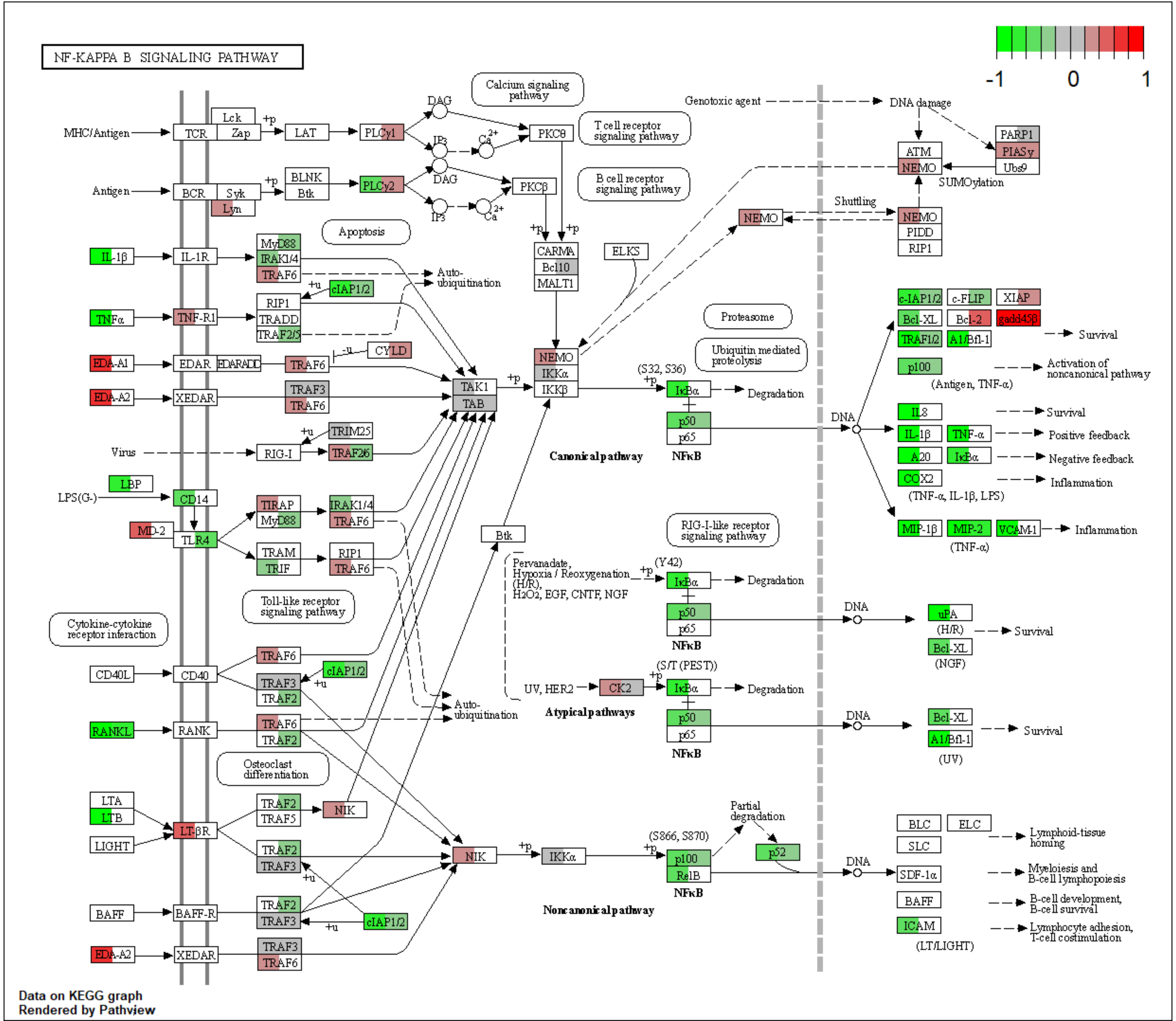

hsa04120

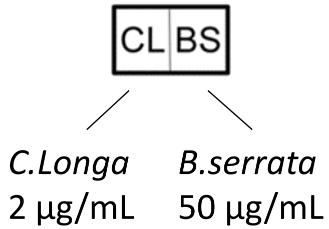

CL
BS
C.Longa
2 µg/mL
B.serrata
50 µg/mL

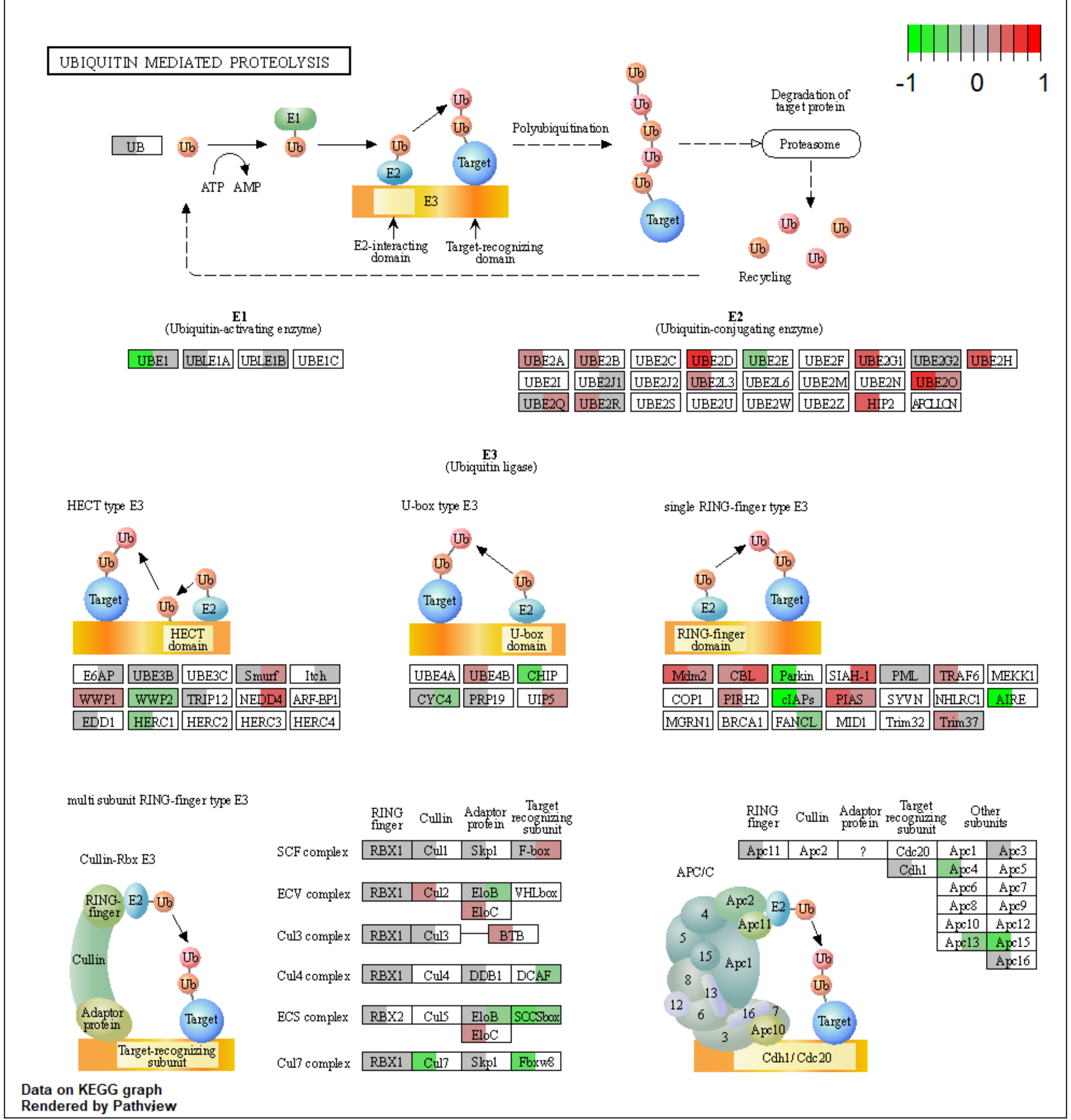

UBIQUITIN MEDIATED PROTEOLYSIS
-1
0
1
UB
Ub
E1
ATP
AMP
E2
E3
Target
E2-interacting domain
Target-recognizing domain
Polyubiquitination
Degradation of target protein
Proteasome
Recycling
E1
(Ubiquitin-activating enzyme)
UBE1
UBLE1A
UBLE1B
UBE1C
E2
(Ubiquitin-conjugating enzyme)
UBE2A
UBE2B
UBE2C
UBE2D
UBE2E
UBE2F
UBE2G1
UBE2G2
UBE2H
UBE2I
UBE2J1
UBE2J2
UBE2L3
UBE2L6
UBE2M
UBE2N
UBE2O
UBE2Q
UBE2R
UBE2S
UBE2U
UBE2W
UBE2Z
HIP2
AFCLLCN
E3
(Ubiquitin ligase)
HECT type E3
HECT domain
E6AP
UBE3B
UBE3C
Smurf
Itch
WWP1
WWP2
TRIP12
NEDD4
ARF-BP1
EDD1
HERC1
HERC2
HERC3
HERC4
U-box type E3
U-box domain
UBE4A
UBE4B
CHIP
CYC4
PRP19
UIP5
single RING-finger type E3
RING-finger domain
Mdm2
CBL
Parkin
SIAH-1
PML
TRAF6
MEKK1
COP1
PIRH2
cIAPs
PIAS
SYVN
NHLRC1
AIRE
MGRN1
BRCA1
FANCL
MID1
Trim32
Trim37
multi subunit RING-finger type E3
Cullin-Rbx E3
RING-finger
Cullin
Adaptor protein
Target-recognizing subunit
RING finger
Cullin
Adaptor protein
Target recognizing subunit
SCF complex
RBX1
Cul1
Skp1
F-box
ECV complex
RBX1
Cul2
EloB
VHLbox
EloC
Cul3 complex
RBX1
Cul3
BTB
Cul4 complex
RBX1
Cul4
DDB1
DCAF
ECS complex
RBX2
Cul5
EloB
SOCSbox
EloC
Cul7 complex
RBX1
Cul7
Skp1
Fbxw8
RING finger
Cullin
Adaptor protein
Target recognizing subunit
Other subunits
Apc11
Apc2
?
Cdc20
Cdh1
Apc1
Apc3
Apc4
Apc5
Apc6
Apc7
Apc8
Apc9
Apc10
Apc12
Apc13
Apc15
Apc16
APC/C
Cdh1 / Cdc20
Data on KEGG graph
Rendered by Pathview

hsa04141

CL | BS

C.Longa 2 µg/mL

B.serrata 50 µg/mL

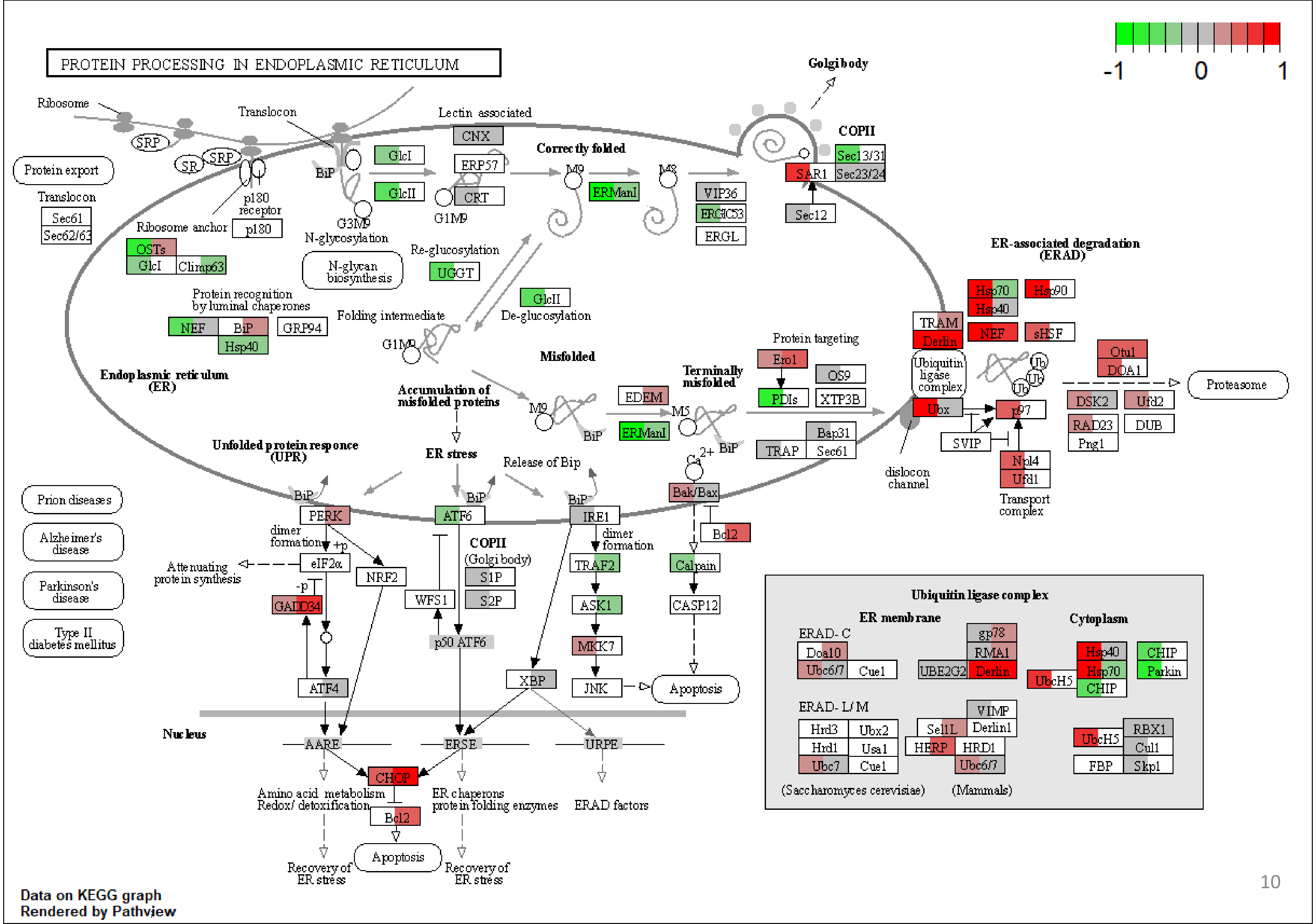

hsa04144

CL | BS

C.Longa 2 μg/mL

B.serrata 50 μg/mL

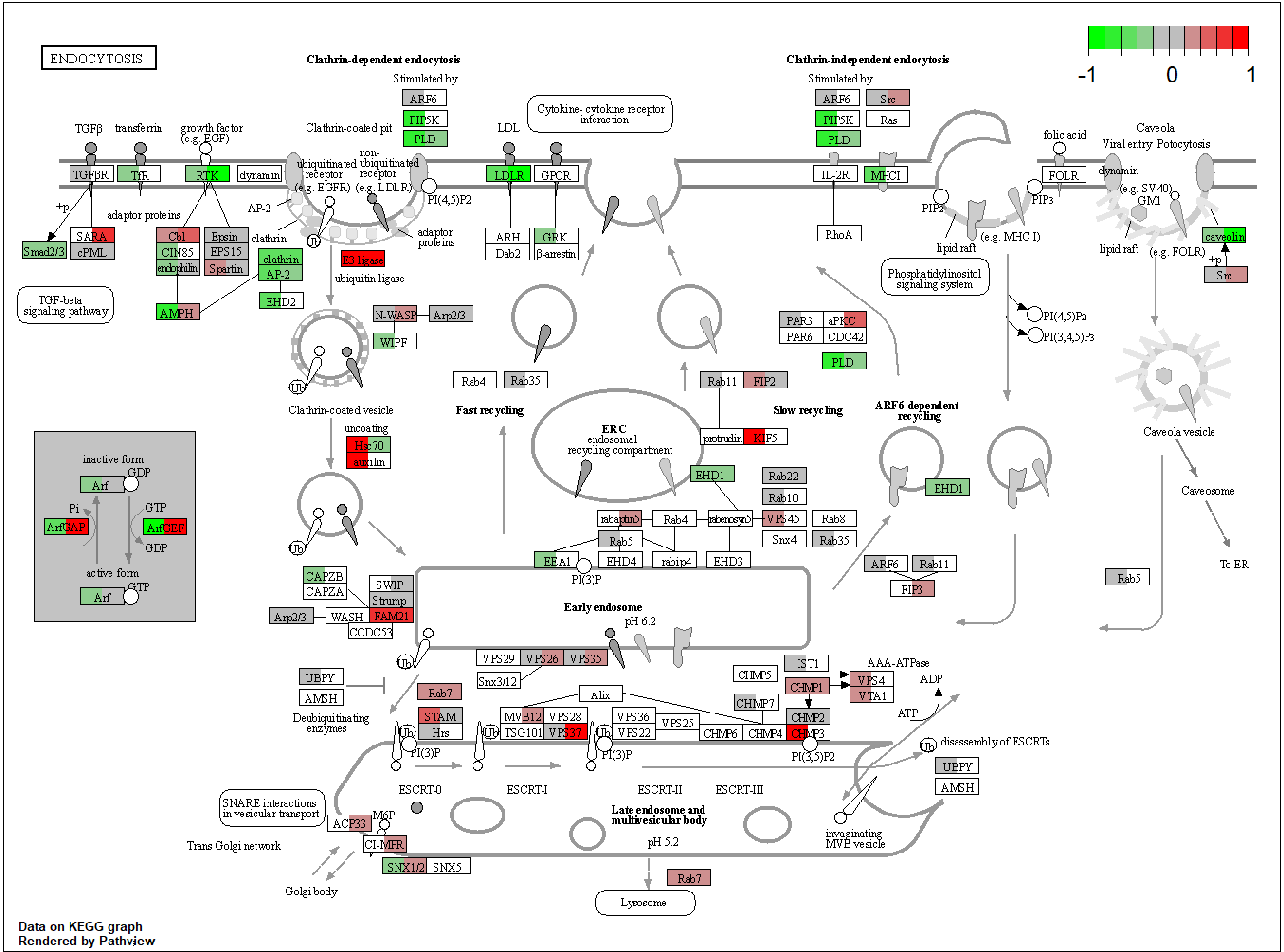

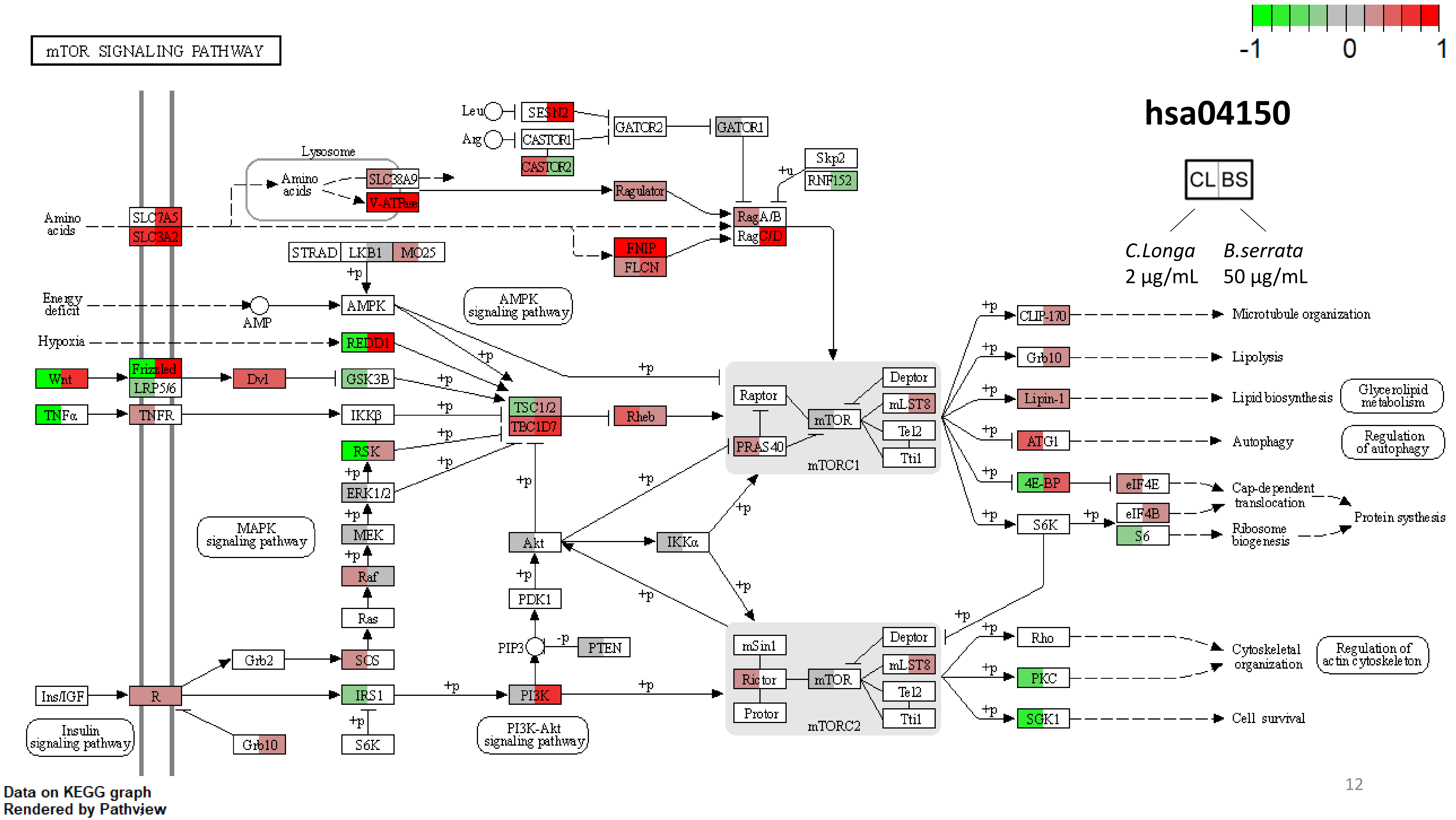
mTOR SIGNALING PATHWAY
-1
0
1
hsa04150
CL
BS
C.Longa
2 µg/mL
B.serrata
50 µg/mL
Leu
Arg
SESN2
CASTOR1
CASTOR2
GATOR2
GATOR1
Skp2
RNF152
+u
Lysosome
Amino acids
SLC38A9
V-ATPase
Ragulator
RagA/B
RagC/D
Amino acids
SLC7A5
SLC3A2
FNIP
FLCN
STRAD
LKB1
MO25
+p
Energy deficit
AMP
AMPK
AMPK signaling pathway
Hypoxia
REDD1
Wnt
Frizzled
LRP5/6
Dvl
GSK3B
TNFα
TNFR
IKKβ
TSC1/2
TBC1D7
Rheb
RSK
ERK1/2
MEK
Raf
Ras
SOS
Grb2
MAPK signaling pathway
Raptor
mTOR
Deptor
mLST8
Tel2
Tti1
PRAS40
mTORC1
Akt
IKKα
PDK1
PIP3
-p
PTEN
PI3K
IRS1
S6K
Ins/IGF
R
Grb10
Insulin signaling pathway
PI3K-Akt signaling pathway
mSin1
Rictor
Protor
mTORC2
CLIP-170
Microtubule organization
Grb10
Lipolysis
Lipin-1
Lipid biosynthesis
Glycerolipid metabolism
ATG1
Autophagy
Regulation of autophagy
4E-BP
eIF4E
Cap-dependent translation
eIF4B
S6K
S6
Ribosome biogenesis
Protein synthesis
Rho
PKC
SGK1
Cytoskeletal organization
Regulation of actin cytoskeleton
Cell survival
Data on KEGG graph
Rendered by Pathview

hsa04216

CL | BS

C.Longa 2 µg/mL

B.serrata 50 µg/mL

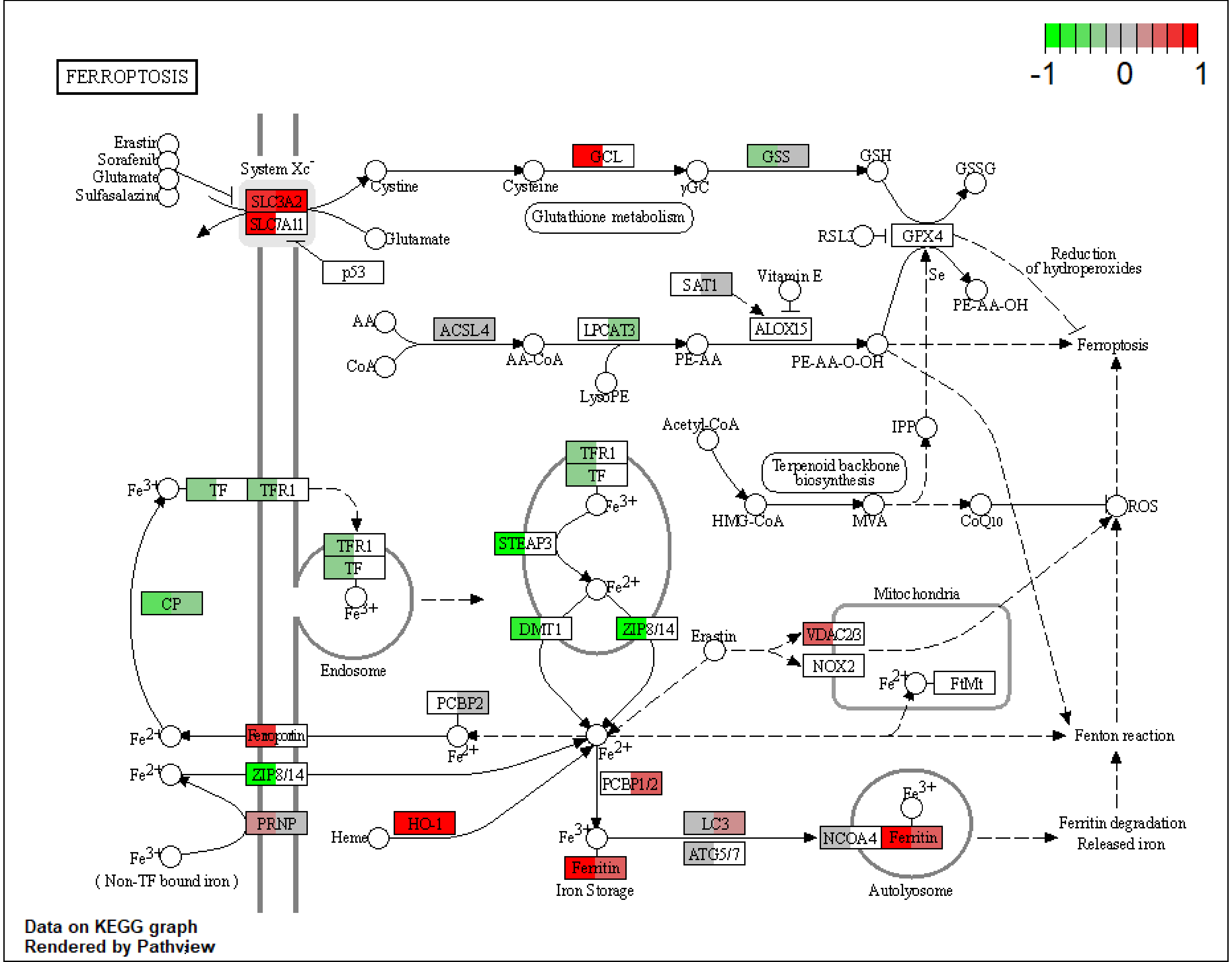

hsa04310

CL | BS

C.Longa 2 μg/mL

B.serrata 50 μg/mL

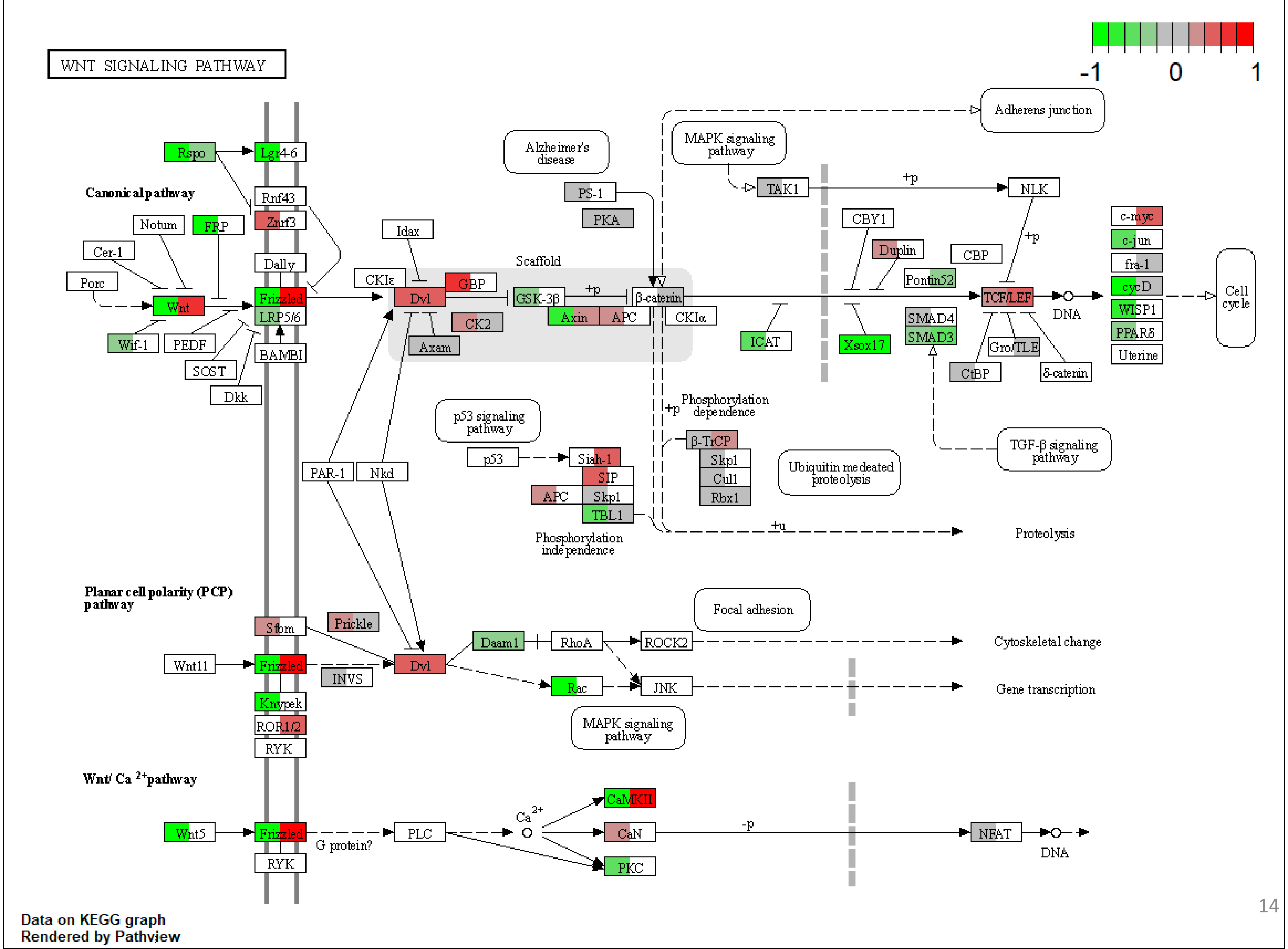

hsa04657

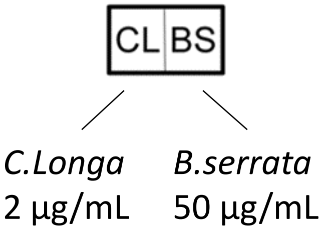
CL
BS
C.Longa
2 µg/mL
B.serrata
50 µg/mL

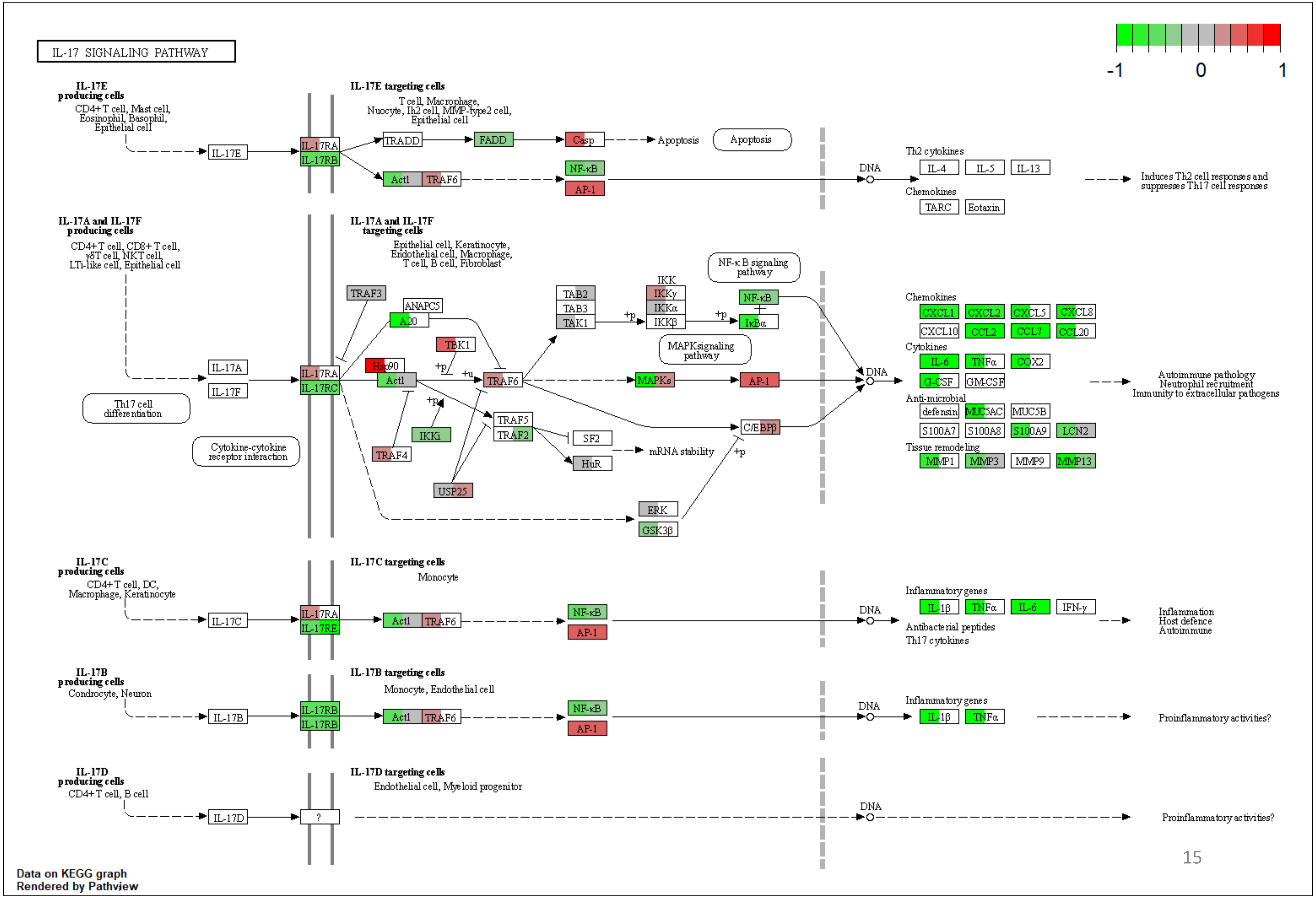
IL-17 SIGNALING PATHWAY
-1
0
1
IL-17E producing cells
CD4+ T cell, Mast cell, Eosinophil, Basophil, Epithelial cell
IL-17E targeting cells
T cell, Macrophage, Nuocyte, Ih2 cell, MPP-type2 cell, Epithelial cell
IL-17E
IL-17RA
IL-17RB
TRADD
FADD
Casp
Apoptosis
Apoptosis
Act1
TRAF6
NF-κB
AP-1
DNA
Th2 cytokines
IL-4
IL-5
IL-13
Chemokines
TARC
Eotaxin
Induces Th2 cell responses and suppresses Th17 cell responses
IL-17A and IL-17F producing cells
CD4+ T cell, CD8+ T cell, γδT cell, NKT cell, LTi-like cell, Epithelial cell
IL-17A and IL-17F targeting cells
Epithelial cell, Keratinocyte, Endothelial cell, Macrophage, T cell, B cell, Fibroblast
IL-17A
IL-17F
IL-17RA
IL-17RC
Th17 cell differentiation
Cytokine-cytokine receptor interaction
TRAF3
ANAPC5
A20
TBK1
Hsp90
Act1
TRAF6
TRAF4
IKKi
USP25
TRAF5
TRAF2
SF2
HuR
mRNA stability
TAB2
TAB3
TAK1
IKK
IKKγ
IKKα
IKKβ
NF-κB signaling pathway
NF-κB
IκBα
MAPK signaling pathway
MAPKs
AP-1
C/EBPβ
ERK
GSK3β
DNA
Chemokines
CXCL1
CXCL2
CXCL5
CXCL8
CXCL10
CCL2
CCL7
CCL20
Cytokines
IL-6
TNFα
COX2
G-CSF
GM-CSF
Anti-microbial
defensin
MUC5AC
MUC5B
S100A7
S100A8
S100A9
LCN2
Tissue remodeling
MMP1
MMP3
MMP9
MMP13
Autoimmune pathology
Neutrophil recruitment
Immunity to extracellular pathogens
IL-17C producing cells
CD4+ T cell, DC, Macrophage, Keratinocyte
IL-17C targeting cells
Monocyte
IL-17C
IL-17RA
IL-17RE
Act1
TRAF6
NF-κB
AP-1
DNA
Inflammatory genes
IL-1β
TNFα
IL-6
IFN-γ
Antibacterial peptides
Th17 cytokines
Inflammation
Host defence
Autoimmune
IL-17B producing cells
Condrocyte, Neuron
IL-17B targeting cells
Monocyte, Endothelial cell
IL-17B
IL-17RB
IL-17RB
Act1
TRAF6
NF-κB
AP-1
DNA
Inflammatory genes
IL-1β
TNFα
Proinflammatory activities?
IL-17D producing cells
CD4+ T cell, B cell
IL-17D targeting cells
Endothelial cell, Myeloid progenitor
IL-17D
?
DNA
Proinflammatory activities?
Data on KEGG graph
Rendered by Pathview
