# Supplementary material for: Curcuma longa and Boswellia serrata Extracts Modulate Different and Complementary Pathways on Human Chondrocytes In Vitro: Deciphering of a Transcriptomic Study
Source: Front Pharmacol. 2022 Aug 11;13:931914. doi: 10.3389/fphar.2022.931914 (PMC9403192; doi:10.3389/fphar.2022.931914)
Supplement: Supplementary file 2 [file DataSheet2.PDF]

Design - Transcriptomic study + proteomic confirmation

Curcuma longa extract (CL), *Boswellia serrata* extract (BS) and CL+BS

Concentration
CL1 : 0.4 µg/mL (1 µM curcumin)
CL2 : 2 µg/mL (5 µM curcumin)
BS1 : 10µg/mL
BS2: 50 µg/mL

Control
CL1
CL2
BS1
BS2
CL1+BS1
CL2+BS2

1 OA patients /culture, alginate beads

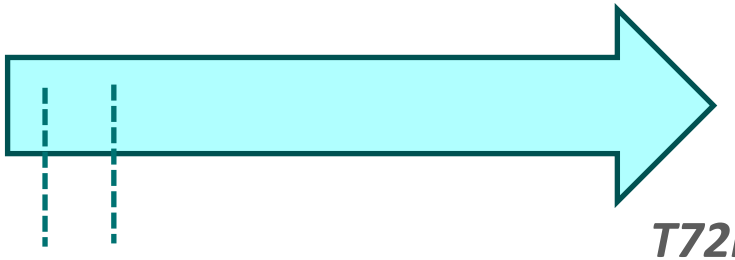

T6h T24h
TRANSCRIPTOMIC
N=10

T72h PROTEOMIC

Supernatant (n=12)
IL6, CCL2, GDF15

Cell
DNA (n=12)
TLR1-2-4-6 surface (flow cytometry) n=5
