# Supplementary material for: Curcuma longa and Boswellia serrata Extracts Modulate Different and Complementary Pathways on Human Chondrocytes In Vitro: Deciphering of a Transcriptomic Study
Source: Front Pharmacol. 2022 Aug 11;13:931914. doi: 10.3389/fphar.2022.931914 (PMC9403192; doi:10.3389/fphar.2022.931914)
Supplement: Supplementary file 3 [file DataSheet6.PDF]

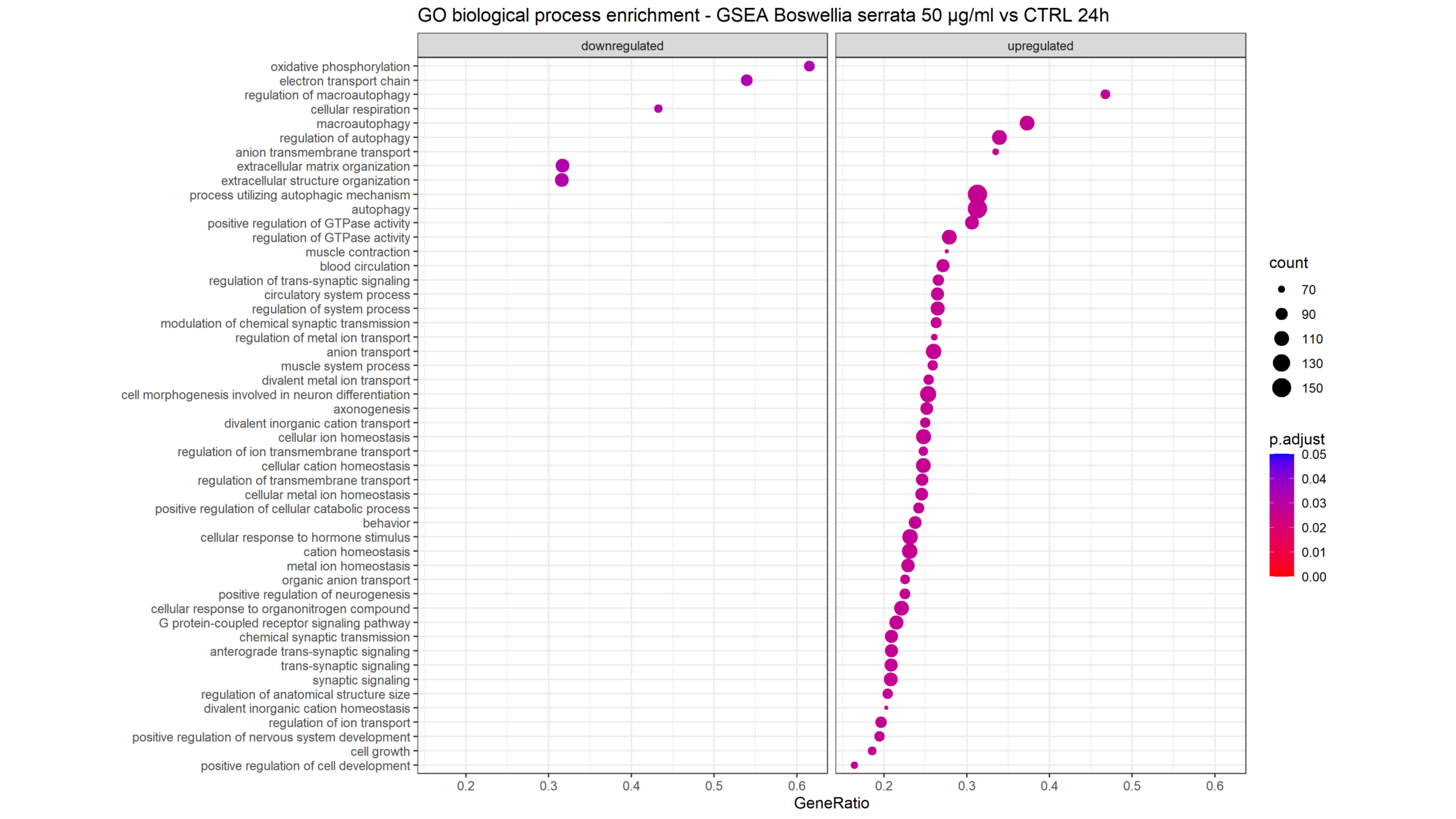
GO biological process enrichment - GSEA Boswellia serrata 50 μg/ml vs CTRL 24h
downregulated
upregulated
oxidative phosphorylation
electron transport chain
regulation of macroautophagy
cellular respiration
macroautophagy
regulation of autophagy
anion transmembrane transport
extracellular matrix organization
extracellular structure organization
process utilizing autophagic mechanism
autophagy
positive regulation of GTPase activity
regulation of GTPase activity
muscle contraction
blood circulation
regulation of trans-synaptic signaling
circulatory system process
regulation of system process
modulation of chemical synaptic transmission
regulation of metal ion transport
anion transport
muscle system process
divalent metal ion transport
cell morphogenesis involved in neuron differentiation
axonogenesis
divalent inorganic cation transport
cellular ion homeostasis
regulation of ion transmembrane transport
cellular cation homeostasis
regulation of transmembrane transport
cellular metal ion homeostasis
positive regulation of cellular catabolic process
behavior
cellular response to hormone stimulus
cation homeostasis
metal ion homeostasis
organic anion transport
positive regulation of neurogenesis
cellular response to organonitrogen compound
G protein-coupled receptor signaling pathway
chemical synaptic transmission
anterograde trans-synaptic signaling
trans-synaptic signaling
synaptic signaling
regulation of anatomical structure size
divalent inorganic cation homeostasis
regulation of ion transport
positive regulation of nervous system development
cell growth
positive regulation of cell development
0.2
0.3
0.4
0.5
0.6
GeneRatio
count
70
90
110
130
150
p.adjust
0.05
0.04
0.03
0.02
0.01
0.00

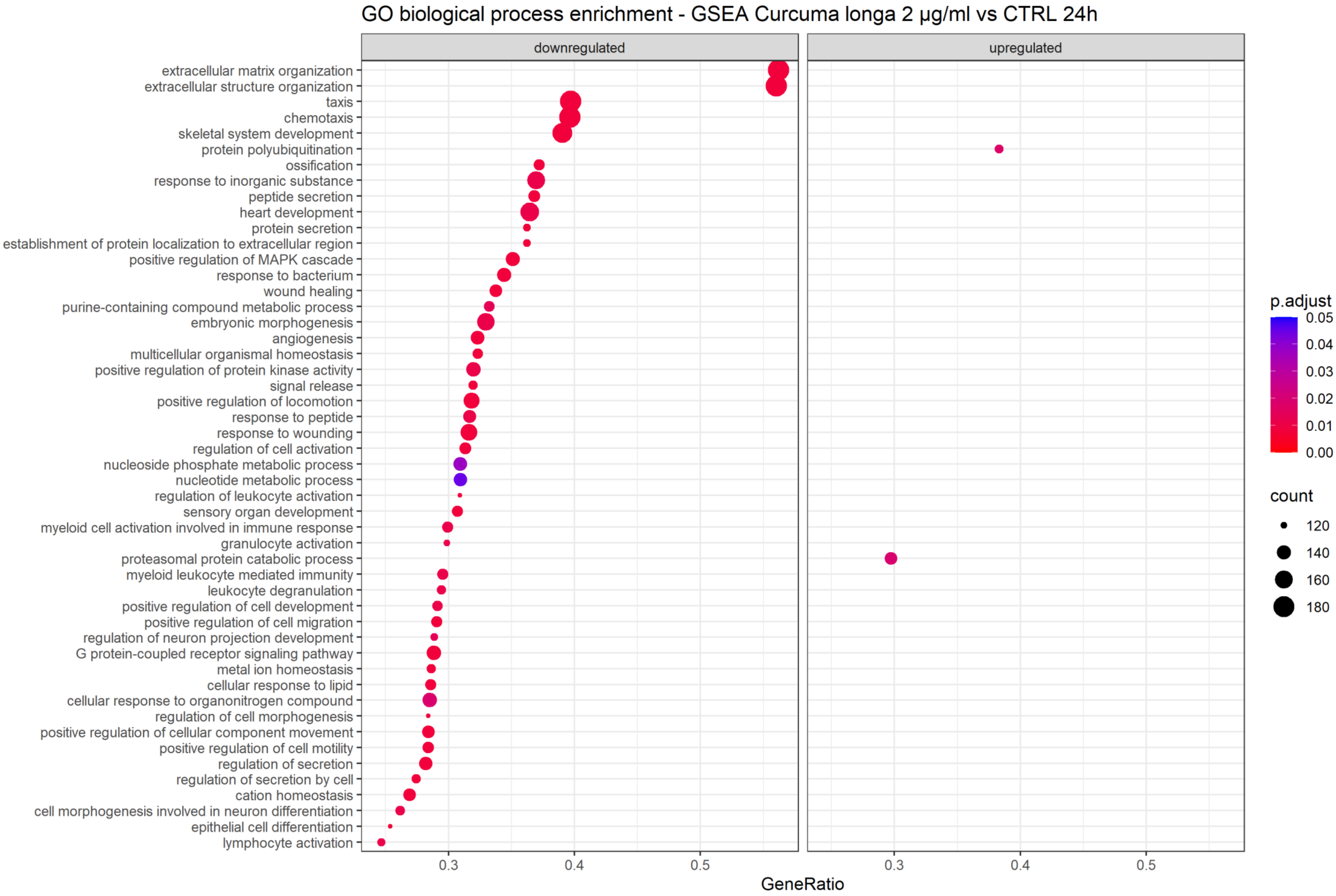
GO biological process enrichment - GSEA Curcuma longa 2 μg/ml vs CTRL 24h
downregulated
upregulated
extracellular matrix organization
extracellular structure organization
taxis
chemotaxis
skeletal system development
protein polyubiquitination
ossification
response to inorganic substance
peptide secretion
heart development
protein secretion
establishment of protein localization to extracellular region
positive regulation of MAPK cascade
response to bacterium
wound healing
purine-containing compound metabolic process
embryonic morphogenesis
angiogenesis
multicellular organismal homeostasis
positive regulation of protein kinase activity
signal release
positive regulation of locomotion
response to peptide
response to wounding
regulation of cell activation
nucleoside phosphate metabolic process
nucleotide metabolic process
regulation of leukocyte activation
sensory organ development
myeloid cell activation involved in immune response
granulocyte activation
proteasomal protein catabolic process
myeloid leukocyte mediated immunity
leukocyte degranulation
positive regulation of cell development
positive regulation of cell migration
regulation of neuron projection development
G protein-coupled receptor signaling pathway
metal ion homeostasis
cellular response to lipid
cellular response to organonitrogen compound
regulation of cell morphogenesis
positive regulation of cellular component movement
positive regulation of cell motility
regulation of secretion
regulation of secretion by cell
cation homeostasis
cell morphogenesis involved in neuron differentiation
epithelial cell differentiation
lymphocyte activation
0.3
0.4
0.5
0.3
0.4
0.5
GeneRatio
p.adjust
0.05
0.04
0.03
0.02
0.01
0.00
count
120
140
160
180

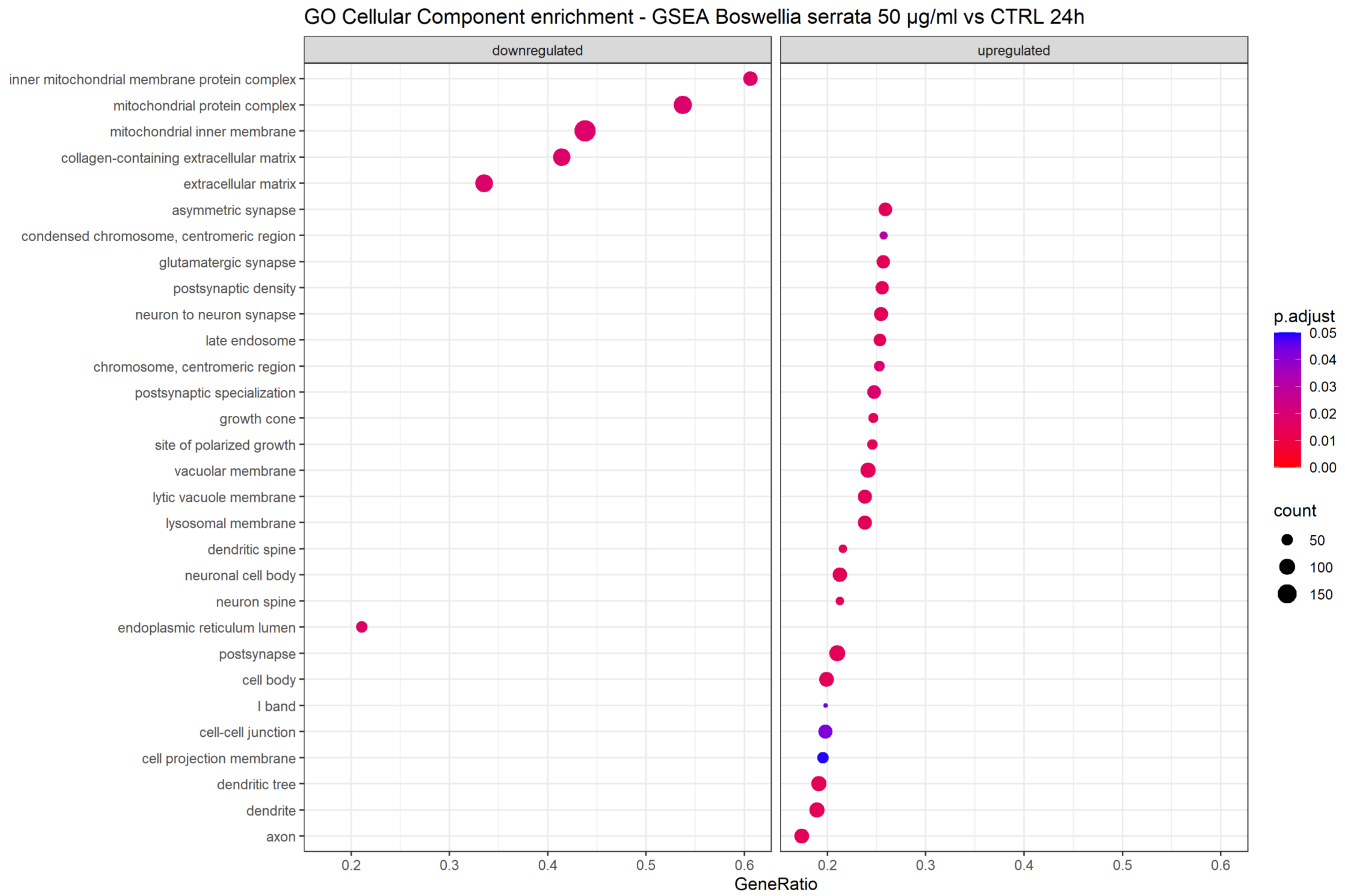
GO Cellular Component enrichment - GSEA Boswellia serrata 50 µg/ml vs CTRL 24h
downregulated
upregulated
inner mitochondrial membrane protein complex
mitochondrial protein complex
mitochondrial inner membrane
collagen-containing extracellular matrix
extracellular matrix
asymmetric synapse
condensed chromosome, centromeric region
glutamatergic synapse
postsynaptic density
neuron to neuron synapse
late endosome
chromosome, centromeric region
postsynaptic specialization
growth cone
site of polarized growth
vacuolar membrane
lytic vacuole membrane
lysosomal membrane
dendritic spine
neuronal cell body
neuron spine
endoplasmic reticulum lumen
postsynapse
cell body
I band
cell-cell junction
cell projection membrane
dendritic tree
dendrite
axon
0.2
0.3
0.4
0.5
0.6
GeneRatio
p.adjust
0.05
0.04
0.03
0.02
0.01
0.00
count
50
100
150

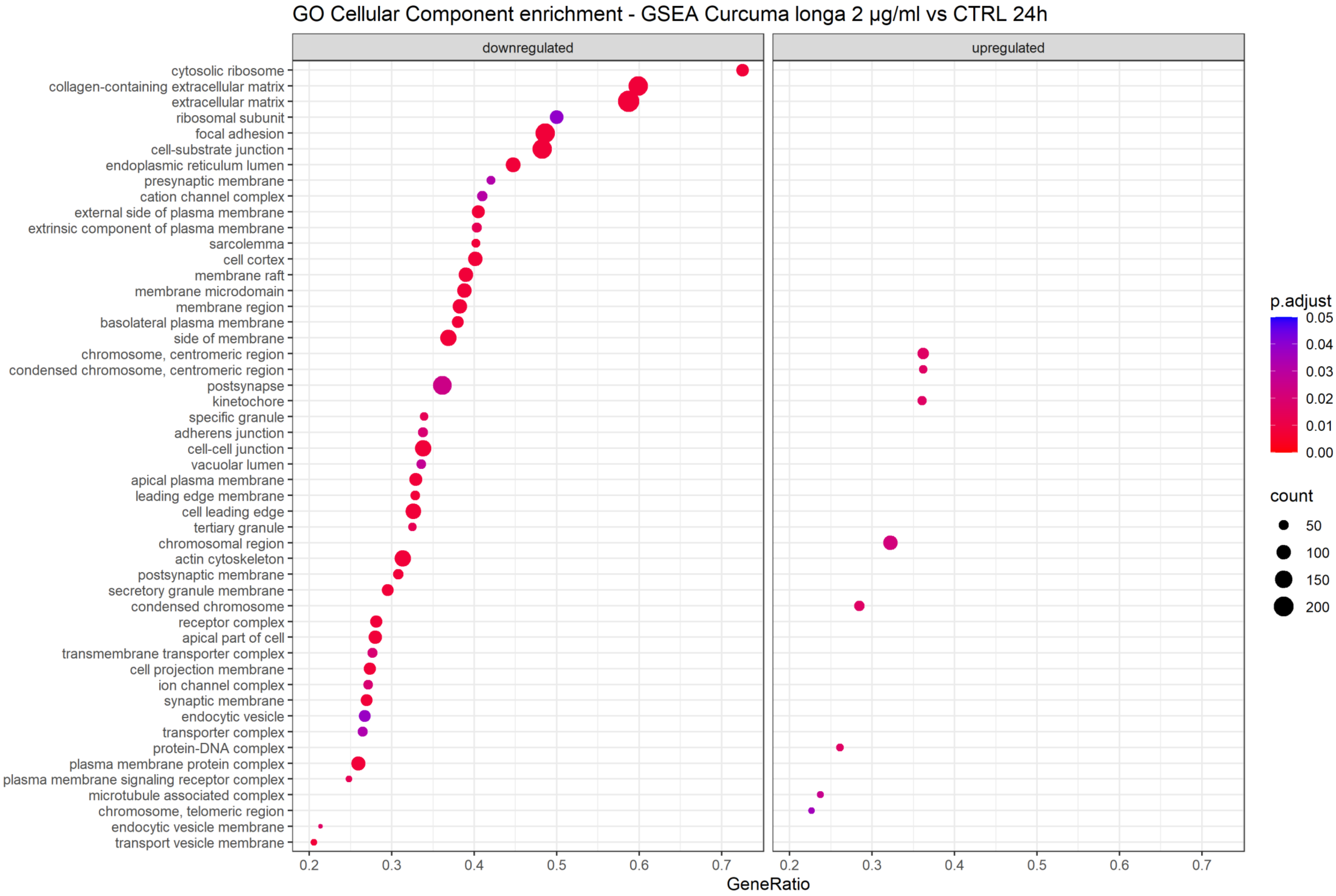
GO Cellular Component enrichment - GSEA Curcuma longa 2 µg/ml vs CTRL 24h
downregulated
upregulated
cytosolic ribosome
collagen-containing extracellular matrix
extracellular matrix
ribosomal subunit
focal adhesion
cell-substrate junction
endoplasmic reticulum lumen
presynaptic membrane
cation channel complex
external side of plasma membrane
extrinsic component of plasma membrane
sarcolemma
cell cortex
membrane raft
membrane microdomain
membrane region
basolateral plasma membrane
side of membrane
chromosome, centromeric region
condensed chromosome, centromeric region
postsynapse
kinetochore
specific granule
adherens junction
cell-cell junction
vacuolar lumen
apical plasma membrane
leading edge membrane
cell leading edge
tertiary granule
chromosomal region
actin cytoskeleton
postsynaptic membrane
secretory granule membrane
condensed chromosome
receptor complex
apical part of cell
transmembrane transporter complex
cell projection membrane
ion channel complex
synaptic membrane
endocytic vesicle
transporter complex
protein-DNA complex
plasma membrane protein complex
plasma membrane signaling receptor complex
microtubule associated complex
chromosome, telomeric region
endocytic vesicle membrane
transport vesicle membrane
0.2 0.3 0.4 0.5 0.6 0.7
0.2 0.3 0.4 0.5 0.6 0.7
GeneRatio
p.adjust
0.05
0.04
0.03
0.02
0.01
0.00
count
50
100
150
200

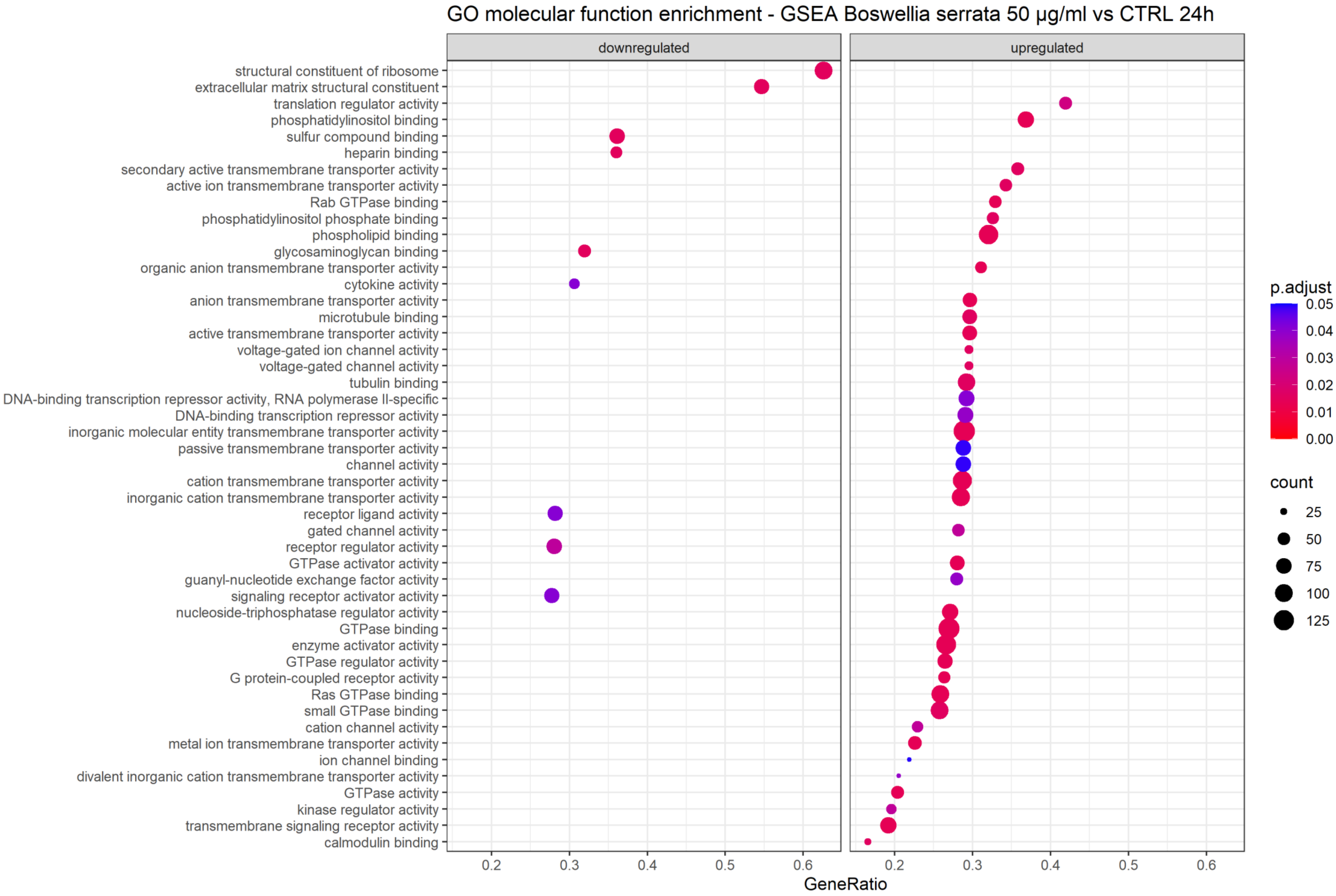
GO molecular function enrichment - GSEA Boswellia serrata 50 µg/ml vs CTRL 24h
downregulated
upregulated
structural constituent of ribosome
extracellular matrix structural constituent
translation regulator activity
phosphatidylinositol binding
sulfur compound binding
heparin binding
secondary active transmembrane transporter activity
active ion transmembrane transporter activity
Rab GTPase binding
phosphatidylinositol phosphate binding
phospholipid binding
glycosaminoglycan binding
organic anion transmembrane transporter activity
cytokine activity
anion transmembrane transporter activity
microtubule binding
active transmembrane transporter activity
voltage-gated ion channel activity
voltage-gated channel activity
tubulin binding
DNA-binding transcription repressor activity, RNA polymerase II-specific
DNA-binding transcription repressor activity
inorganic molecular entity transmembrane transporter activity
passive transmembrane transporter activity
channel activity
cation transmembrane transporter activity
inorganic cation transmembrane transporter activity
receptor ligand activity
gated channel activity
receptor regulator activity
GTPase activator activity
guanyl-nucleotide exchange factor activity
signaling receptor activator activity
nucleoside-triphosphatase regulator activity
GTPase binding
enzyme activator activity
GTPase regulator activity
G protein-coupled receptor activity
Ras GTPase binding
small GTPase binding
cation channel activity
metal ion transmembrane transporter activity
ion channel binding
divalent inorganic cation transmembrane transporter activity
GTPase activity
kinase regulator activity
transmembrane signaling receptor activity
calmodulin binding
0.2
0.3
0.4
0.5
0.6
GeneRatio
p.adjust
0.05
0.04
0.03
0.02
0.01
0.00
count
25
50
75
100
125

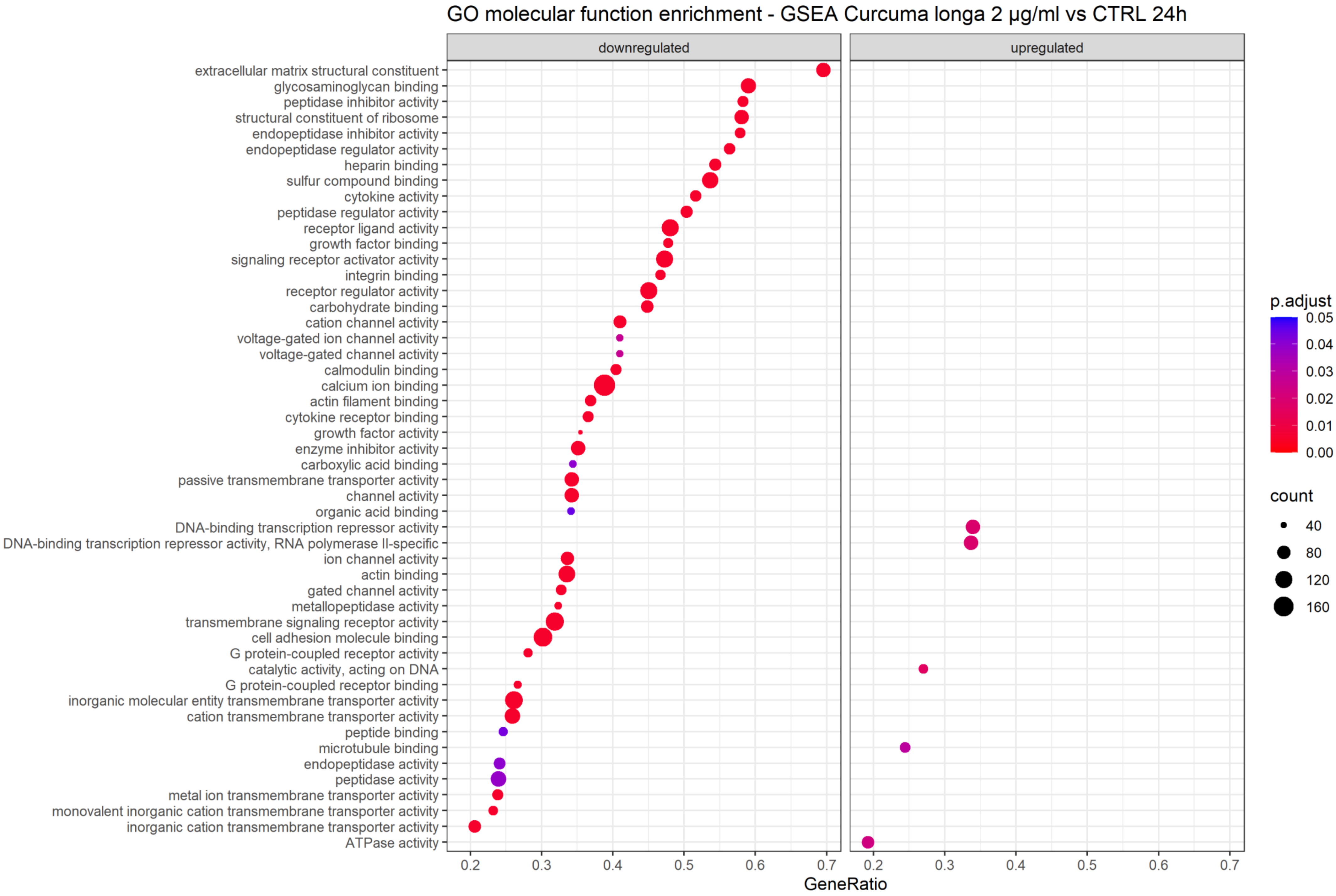
GO molecular function enrichment - GSEA Curcuma longa 2 µg/ml vs CTRL 24h
downregulated
upregulated
extracellular matrix structural constituent
glycosaminoglycan binding
peptidase inhibitor activity
structural constituent of ribosome
endopeptidase inhibitor activity
endopeptidase regulator activity
heparin binding
sulfur compound binding
cytokine activity
peptidase regulator activity
receptor ligand activity
growth factor binding
signaling receptor activator activity
integrin binding
receptor regulator activity
carbohydrate binding
cation channel activity
voltage-gated ion channel activity
voltage-gated channel activity
calmodulin binding
calcium ion binding
actin filament binding
cytokine receptor binding
growth factor activity
enzyme inhibitor activity
carboxylic acid binding
passive transmembrane transporter activity
channel activity
organic acid binding
DNA-binding transcription repressor activity
DNA-binding transcription repressor activity, RNA polymerase II-specific
ion channel activity
actin binding
gated channel activity
metallopeptidase activity
transmembrane signaling receptor activity
cell adhesion molecule binding
G protein-coupled receptor activity
catalytic activity, acting on DNA
G protein-coupled receptor binding
inorganic molecular entity transmembrane transporter activity
cation transmembrane transporter activity
peptide binding
microtubule binding
endopeptidase activity
peptidase activity
metal ion transmembrane transporter activity
monovalent inorganic cation transmembrane transporter activity
inorganic cation transmembrane transporter activity
ATPase activity
0.2
0.3
0.4
0.5
0.6
0.7
GeneRatio
p.adjust
0.05
0.04
0.03
0.02
0.01
0.00
count
40
80
120
160

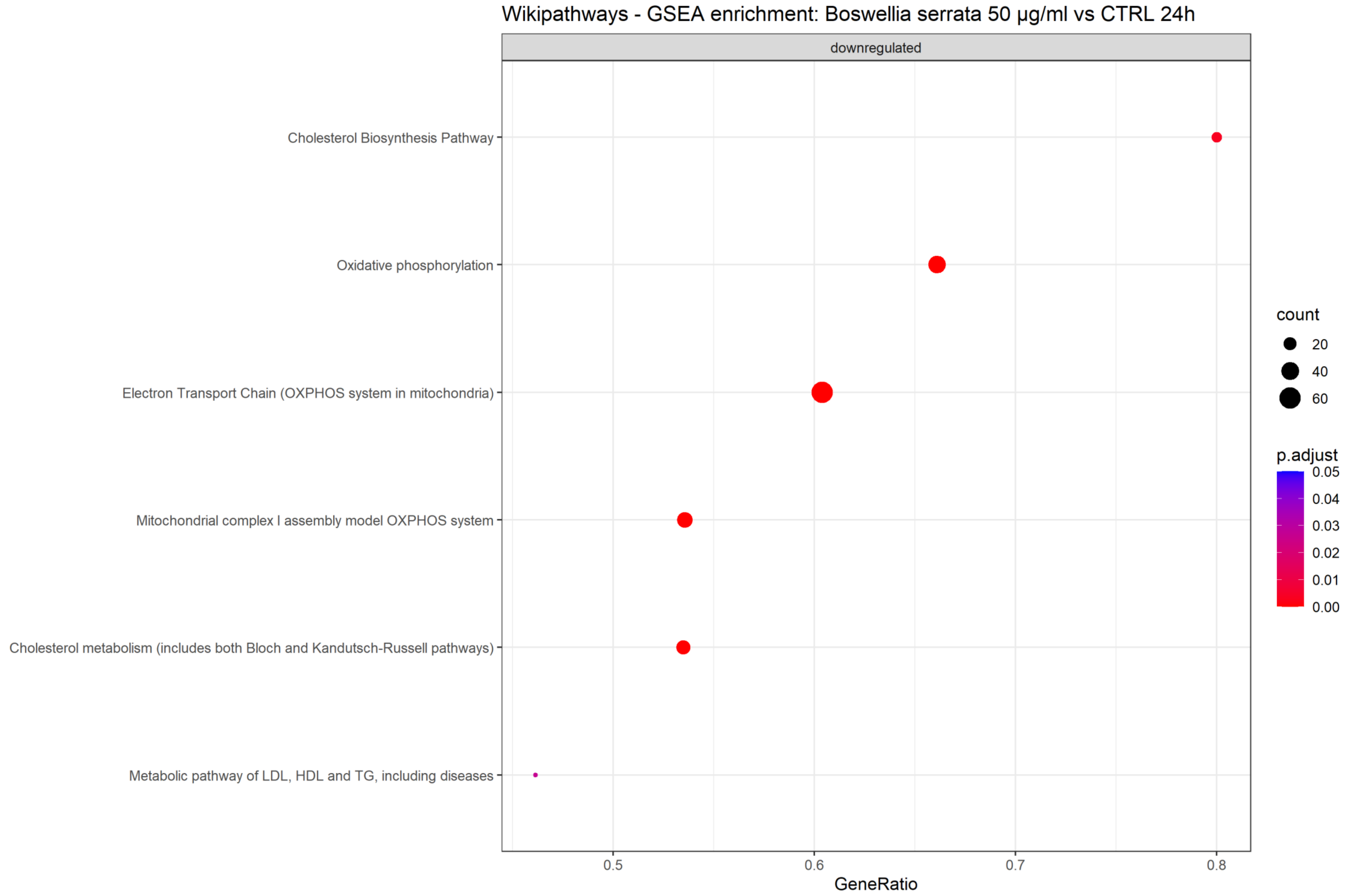

Wikipathways - GSEA enrichment: Boswellia serrata 50 µg/ml vs CTRL 24h
downregulated
Cholesterol Biosynthesis Pathway
Oxidative phosphorylation
Electron Transport Chain (OXPHOS system in mitochondria)
Mitochondrial complex I assembly model OXPHOS system
Cholesterol metabolism (includes both Bloch and Kandutsch-Russell pathways)
Metabolic pathway of LDL, HDL and TG, including diseases
0.5
0.6
0.7
0.8
GeneRatio
count
20
40
60
p.adjust
0.05
0.04
0.03
0.02
0.01
0.00

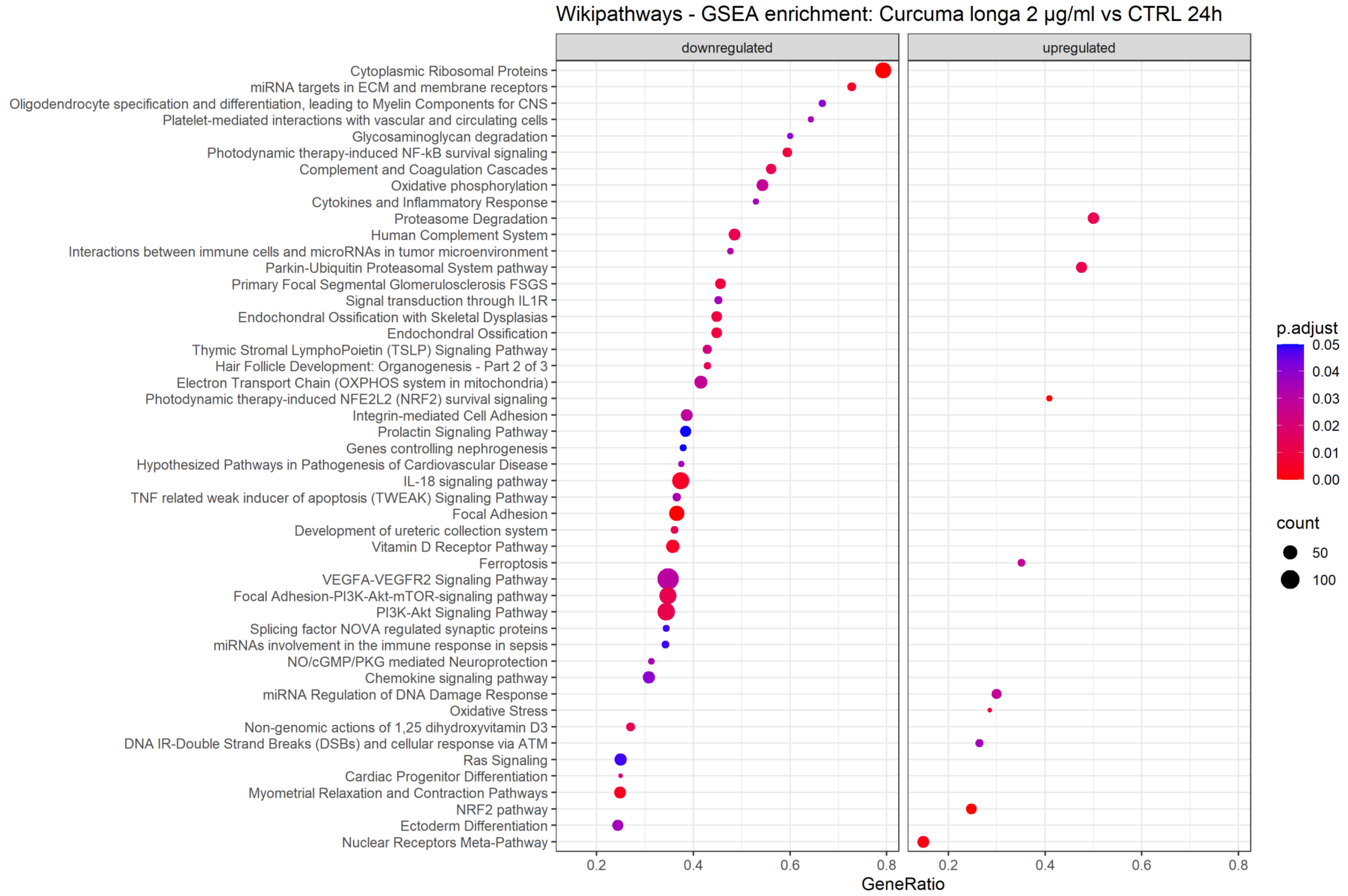
Wikipathways - GSEA enrichment: Curcuma longa 2 µg/ml vs CTRL 24h
downregulated
upregulated
Cytoplasmic Ribosomal Proteins
miRNA targets in ECM and membrane receptors
Oligodendrocyte specification and differentiation, leading to Myelin Components for CNS
Platelet-mediated interactions with vascular and circulating cells
Glycosaminoglycan degradation
Photodynamic therapy-induced NF-kB survival signaling
Complement and Coagulation Cascades
Oxidative phosphorylation
Cytokines and Inflammatory Response
Proteasome Degradation
Human Complement System
Interactions between immune cells and microRNAs in tumor microenvironment
Parkin-Ubiquitin Proteasomal System pathway
Primary Focal Segmental Glomerulosclerosis FSGS
Signal transduction through IL1R
Endochondral Ossification with Skeletal Dysplasias
Endochondral Ossification
Thymic Stromal LymphoPoietin (TSLP) Signaling Pathway
Hair Follicle Development: Organogenesis - Part 2 of 3
Electron Transport Chain (OXPHOS system in mitochondria)
Photodynamic therapy-induced NFE2L2 (NRF2) survival signaling
Integrin-mediated Cell Adhesion
Prolactin Signaling Pathway
Genes controlling nephrogenesis
Hypothesized Pathways in Pathogenesis of Cardiovascular Disease
IL-18 signaling pathway
TNF related weak inducer of apoptosis (TWEAK) Signaling Pathway
Focal Adhesion
Development of ureteric collection system
Vitamin D Receptor Pathway
Ferroptosis
VEGFA-VEGFR2 Signaling Pathway
Focal Adhesion-PI3K-Akt-mTOR-signaling pathway
PI3K-Akt Signaling Pathway
Splicing factor NOVA regulated synaptic proteins
miRNAs involvement in the immune response in sepsis
NO/cGMP/PKG mediated Neuroprotection
Chemokine signaling pathway
miRNA Regulation of DNA Damage Response
Oxidative Stress
Non-genomic actions of 1,25 dihydroxyvitamin D3
DNA IR-Double Strand Breaks (DSBs) and cellular response via ATM
Ras Signaling
Cardiac Progenitor Differentiation
Myometrial Relaxation and Contraction Pathways
NRF2 pathway
Ectoderm Differentiation
Nuclear Receptors Meta-Pathway
0.2
0.4
0.6
0.8
0.2
0.4
0.6
0.8
GeneRatio
p.adjust
0.05
0.04
0.03
0.02
0.01
0.00
count
50
100
