# Supplementary material for: Curcuma longa and Boswellia serrata Extracts Modulate Different and Complementary Pathways on Human Chondrocytes In Vitro: Deciphering of a Transcriptomic Study
Source: Front Pharmacol. 2022 Aug 11;13:931914. doi: 10.3389/fphar.2022.931914 (PMC9403192; doi:10.3389/fphar.2022.931914)
Supplement: Supplementary file 5 [file DataSheet9.DOCX]

***Table 1****:* *DEGs from Nrf1 pathways significantly modified after 24h treatment with 50µg/mL of B. serrata extract. Red color according to log2FoldChange. baseMean : mean count, padj :adjusted p-value.*

| **BaseMean** | **log2FoldChange** | **padj** | **FC** | **Symbol** | **Gene name** |
| --- | --- | --- | --- | --- | --- |
| 348 | 0.21 | 4.0E-02 | +15% | BLVRB | biliverdin reductase B |
| 56001 | 0.54 | 5.8E-05 | +45% | FTL | ferritin light chain |
| 49379 | 1.50 | 1.3E-08 | x2.8 | HMOX1 | heme oxygenase 1 |
|  |  |  |  |  |  |
| 4769 | 0.64 | 1.1E-06 | +56% | MT1E | metallothionein 1E |
| 1009 | 0.93 | 1.7E-04 | +91% | MT1F | metallothionein 1F |
| 6270 | 1.12 | 4.8E-10 | x2.2 | MT1G | metallothionein 1G |
| 1537 | 1.09 | 1.8E-06 | x2.1 | MT1H | metallothionein 1H |
| 24 | 0.94 | 1.5E-03 | +91% | MT1HL1 | metallothionein 1H like 1 |
| 402 | 0.66 | 8.8E-04 | +57% | MT1L | metallothionein 1L, pseudogene |
| 690 | 0.89 | 6.3E-05 | +86% | MT1M | metallothionein 1M |
| 1130 | 1.21 | 7.1E-21 | x2.3 | MT1X | metallothionein 1X |
| 47969 | 0.50 | 2.2E-05 | +41% | MT2A | metallothionein 2A |
|  |  |  |  |  |  |
| 2893 | 0.44 | 3.6E-16 | +36% | BAG3 | BAG cochaperone 3 |
| 1190 | 0.58 | 2.4E-10 | +49% | DNAJB9 | DnaJ heat shock protein family (Hsp40) member B9 |
| 142 | 0.47 | 7.7E-05 | +39% | HSPA4L | heat shock protein family A (Hsp70) member 4 like |
| 18625 | 0.32 | 2.6E-07 | +24% | HSPA5 | heat shock protein family A (Hsp70) member 5 |
| 5928 | 0.38 | 2.1E-09 | +30% | HSPA9 | heat shock protein family A (Hsp70) member 9 |
| 1361 | 0.34 | 1.1E-08 | +26% | HSPA13 | heat shock protein family A (Hsp70) member 13 |
|  |  |  |  |  |  |
| 1653 | 0.67 | 2.7E-11 | +60% | MAFG | MAF bZIP transcription factor G |
| 44323 | 0.62 | 2.1E-08 | +54% | SQSTM1 | sequestosome 1 |

***Table 2****:* *DEGs from « Inflammatory response GO :0006954 » pathways significantly modified after 24h treatment with 50µg/mL of B. serrata extract. Red color according to log2FoldChange. baseMean : mean count, padj :adjusted p-value.*

| **baseMean** | **log2FoldChange** | **changement (%)** | **padj** | **Symbol** | **GeneName** |
| --- | --- | --- | --- | --- | --- |
| 3084 | -1.11 | -54 | 9.1E-06 | IL6 | interleukin 6 |
| 54 | -1.13 | -54 | 1.0E-09 | TNFSF10 | TNF superfamily member 10 |
| 10 | -1.10 | -53 | 2.3E-03 | CCL7 | C-C motif chemokine ligand 7 |
| 328 | -0.88 | -45 | 2.7E-03 | CCL2 | C-C motif chemokine ligand 2 |
| 35 | -0.86 | -45 | 7.3E-06 | IL17RE | interleukin 17 receptor E |
| 311 | -0.77 | -42 | 1.2E-03 | CXCL3 | C-X-C motif chemokine ligand 3 |
| 30 | -0.76 | -41 | 6.5E-03 | SELP | selectin P |
| 42 | -0.65 | -36 | 2.4E-04 | C3AR1 | complement C3a receptor 1 |
| 6489 | -0.63 | -35 | 2.6E-08 | FSTL1 | follistatin like 1 |
| 953 | -0.54 | -31 | 1.4E-09 | TLR4 | toll like receptor 4 |
| 154 | -0.54 | -31 | 5.1E-09 | TLR1 | toll like receptor 1 |
| 41 | -0.54 | -31 | 8.2E-04 | AGER | advanced glycosylation end-product specific receptor |
| 1064 | -0.50 | -29 | 4.2E-04 | LOXL3 | lysyl oxidase like 3 |
| 772 | -0.49 | -29 | 5.9E-04 | IGFBP4 | insulin like growth factor binding protein 4 |
| 483 | -0.49 | -29 | 9.2E-06 | IL17RB | interleukin 17 receptor B |
| 392 | -0.46 | -27 | 1.7E-07 | IL16 | interleukin 16 |
| 132 | -0.45 | -27 | 1.6E-02 | CX3CL1 | C-X3-C motif chemokine ligand 1 |
| 301 | -0.44 | -27 | 6.2E-05 | ZNF580 | zinc finger protein 580 |
| 666 | -0.41 | -25 | 2.1E-08 | IL17RC | interleukin 17 receptor C |
| 181 | -0.41 | -25 | 2.7E-02 | PTGER4 | prostaglandin E receptor 4 |
| 598 | -0.40 | -24 | 1.5E-14 | CDO1 | cysteine dioxygenase type 1 |
| 712 | -0.37 | -23 | 1.8E-06 | APOL3 | apolipoprotein L3 |
| 210 | -0.35 | -22 | 2.6E-05 | HYAL1 | hyaluronidase 1 |
| 1905 | -0.32 | -20 | 6.0E-07 | PLA2G2A | phospholipase A2 group IIA |
| 237 | -0.31 | -20 | 1.8E-05 | TLR6 | toll like receptor 6 |
| 500 | -0.31 | -20 | 4.2E-11 | MYD88 | MYD88 innate immune signal transduction adaptor |
| 141 | -0.30 | -19 | 5.6E-03 | ITGB2 | integrin subunit beta 2 |
| 4354 | -0.29 | -18 | 3.6E-04 | TNIP1 | TNFAIP3 interacting protein 1 |
| 123 | -0.28 | -18 | 1.8E-02 | PTGFR | prostaglandin F receptor |
| 163 | -0.27 | -17 | 3.6E-03 | PLGRKT | plasminogen receptor with a C-terminal lysine |
| 122613 | -0.25 | -16 | 6.3E-03 | CHI3L1 | chitinase 3 like 1 |
| 1790 | -0.24 | -15 | 1.3E-02 | AXL | AXL receptor tyrosine kinase |
| 735 | 0.55 | 46 | 7.5E-16 | HMGB2 | high mobility group box 2 |
| 74 | 0.62 | 53 | 1.3E-05 | PTGER3 | prostaglandin E receptor 3 |
| 123789 | 0.68 | 60 | 5.5E-10 | SPP1 | secreted phosphoprotein 1 |
| 404 | 0.73 | 66 | 2.9E-05 | TNFRSF1B | TNF receptor superfamily member 1B |
| 110 | 0.75 | 68 | 2.2E-09 | IL36B | interleukin 36 beta |
| 97 | 1.02 | 102 | 3.6E-11 | IL36RN | interleukin 36 receptor antagonist |
| 30 | 1.06 | 109 | 7.8E-07 | NRROS | negative regulator of reactive oxygen species |
| 1763 | 1.07 | 110 | 8.4E-12 | CYP26B1 | cytochrome P450 family 26 subfamily B member 1 |
| 210 | 1.40 | 165 | 1.4E-58 | CCRL2 | C-C motif chemokine receptor like 2 |

***Table 3****: DEGs involved in extracellular matrix degradation significantly modified after 24h treatment with 50 µg/mL of B. serrata extract. Blue/red color according to log2FoldChange. baseMean : mean count, padj :adjusted p-value.*

| **baseMean** | **log2FoldChange** | **padj** | **Changement %** | **Symbol** | **GeneName** |
| --- | --- | --- | --- | --- | --- |
| 1413 | -0.76 | 1.7E-15 | -41 | ADAMTS1 | ADAM metallopeptidase with thrombospondin motif 1 |
| 649 | -0.73 | 7.0E-12 | -40 | ADAMTS5 | ADAM metallopeptidase with thrombospondin motif 5 |
| 795 | -0.45 | 4.5E-11 | -27 | CTSC | cathepsin C |
| 20155 | -0.37 | 1.5E-03 | -23 | MMP13 | matrix metallopeptidase 13 |
| 646 | -0.27 | 5.7E-05 | -17 | SERPINB1 | serpin family B member 1 |
| 154 | -0.26 | 6.4E-03 | -16 | SERPINB9 | serpin family B member 9 |
| 1353040 | -0.18 | 1.1E-03 | -11 | MMP3 | matrix metallopeptidase 3 |
| 20159 | 0.30 | 4.6E-04 | 23 | MMP14 | matrix metallopeptidase 14 |
| 84 | 0.34 | 1.0E-02 | 27 | ADAM19 | ADAM metallopeptidase domain 19 |
| 7663 | 0.41 | 2.2E-21 | 32 | CTSD | cathepsin D |
| 36 | 0.49 | 3.5E-02 | 41 | MMP15 | matrix metallopeptidase 15 |
| 15842 | 0.68 | 9.9E-22 | 60 | CTSL | cathepsin L |
| 49 | 0.96 | 1.2E-04 | 95 | SERPINA3 | serpin family A member 3 |

***Table 4****: DEGs from cartilage development and endochondral ossification pathways significantly modified after 24h treatment with 50 µg/mL of B. serrata extract. Blue/red color according to log2FoldChange. baseMean : mean count, padj :adjusted p-value.*

| **baseMean** | **log2FoldChange** | **padj** | **FC (%)** | **Symbol** | **GeneName** |
| --- | --- | --- | --- | --- | --- |
| 9 | -1.09 | 3.2E-03 | -53 | COL21A1 | collagen type XXI alpha 1 chain |
| 4554 | -1.07 | 1.7E-10 | -52 | CCN1 | cellular communication network factor 1 |
| 10734 | -1.00 | 2.2E-21 | -50 | COL3A1 | collagen type III alpha 1 chain |
| 10 | -0.82 | 2.6E-02 | -44 | ASPN | asporin |
| 52 | -0.84 | 2.8E-07 | -44 | IBSP | Bone sialoprotein |
| 102 | -0.82 | 1.6E-07 | -43 | POSTN | periostin |
| 35 | -0.81 | 1.9E-02 | -43 | COL5A3 | collagen type V alpha 3 chain |
| 62 | -0.77 | 4.7^E^07 | -41 | BMP5 | bone morphogenetic protein 5 |
| 515 | -0.71 | 3.3E-04 | -39 | NDP | norrin cystine knot growth factor NDP |
| 240 | -0.68 | 1.9E-03 | -38 | COL1A1 | collagen type I alpha 1 chain |
| 35 | -0.68 | 2.7E-04 | -38 | OMD | osteomodulin |
| 12065 | -0.68 | 3.7E-06 | -38 | COL2A1 | collagen type II alpha 1 chain |
| 33 | -0.63 | 8.9E-03 | -35 | COL8A1 | collagen type VIII alpha 1 chain |
| 94 | -0.53 | 4.3E-04 | -31 | FBN2 | fibrillin 2 |
| 4463 | -0.53 | 1.7E-11 | -31 | COL5A2 | collagen type V alpha 2 chain |
| 1662 | -0.52 | 1.0E-04 | -30 | COL7A1 | collagen type VII alpha 1 chain |
| 865 | -0.48 | 4.2E-02 | -29 | FGF1 | fibroblast growth factor 1 |
| 4659 | -0.48 | 1.7E-04 | -28 | COL11A1 | collagen type XI alpha 1 chain |
| 33 | -0.46 | 8.1E-03 | -27 | FBLN5 | fibulin 5 |
| 77 | -0.45 | 1.9E-02 | -27 | MXRA5 | matrix remodeling associated 5 |
| 230 | -0.44 | 2.6E-02 | -27 | WNT5A | Wnt family member 5A |
| 286 | -0.44 | 1.3E-03 | -26 | LAMB1 | laminin subunit beta 1 |
| 11471 | -0.42 | 5.5E-05 | -25 | TNC | tenascin C |
| 3564 | -0.39 | 3.8E-04 | -24 | FBN1 | fibrillin 1 |
| 120 | -0.39 | 3.7E-03 | -23 | VCAN | versican |
| 1125 | -0.38 | 1.3E-03 | -23 | COL10A1 | collagen type X alpha 1 chain |
| 1226 | -0.38 | 5.3E-07 | -23 | SDC3 | syndecan 3 |
| 1060 | -0.37 | 7.9E-08 | -23 | LGALS1 | galectin 1 |
| 1793 | -0.35 | 1.1E-04 | -21 | COL1A2 | collagen type I alpha 2 chain |
| 1511 | -0.33 | 3.2E-05 | -21 | SRPX2 | sushi repeat containing protein X-linked 2 |
| 1994 | -0.33 | 3.2E-03 | -20 | COL12A1 | collagen type XII alpha 1 chain |
| 1512 | -0.32 | 1.5E-02 | -20 | COL5A1 | collagen type V alpha 1 chain |
| 641 | -0.32 | 3.5E-07 | -20 | EFEMP1 | EGF containing fibulin extracellular matrix protein 1 |
| 416 | -0.32 | 4.7E-03 | -20 | PODNL1 | podocan like 1 |
| 9202 | -0.31 | 2.5E-02 | -20 | LTBP2 | latent transforming growth factor beta binding protein 2 |
| 642 | -0.31 | 2.2E-06 | -19 | SLPI | secretory leukocyte peptidase inhibitor |
| 2580 | -0.30 | 3.6E-05 | -19 | COL27A1 | collagen type XXVII alpha 1 chain |
| 136 | -0.29 | 8.2E-03 | -18 | OGN | osteoglycin |
| 609 | -0.29 | 7.3E-03 | -18 | MATN3 | matrilin 3 |
| 601 | -0.29 | 9.7E-05 | -18 | IGFBP7 | insulin like growth factor binding protein 7 |
| 103122 | -0.28 | 1.1E-08 | -18 | DCN | decorin |
| 5358 | -0.24 | 4.1E-02 | -15 | SCARA3 | scavenger receptor class A member 3 |
| 689 | -0.24 | 3.9E-02 | -15 | AGRN | agrin |
| 69196 | -0.23 | 6.9E-03 | -15 | LUM | lumican |
| 742 | 0.23 | 8.6E-07 | 17 | ANXA4 | annexin A4 |
| 1919 | 0.24 | 3.8E-04 | 18 | SDC2 | syndecan 2 |
| 4480 | 0.26 | 2.2E-03 | 20 | EMILIN1 | elastin microfibril interfacer 1 |
| 80 | 0.36 | 2.6E-02 | 28 | COL4A1 | collagen type IV alpha 1 chain |
| 309 | 0.39 | 4.1E-06 | 31 | LAMA1 | laminin subunit alpha 1 |
| 192 | 0.40 | 5.1E-04 | 32 | THBS2 | thrombospondin 2 |
| 344 | 0.46 | 1.9E-03 | 38 | THSD4 | thrombospondin type 1 domain containing 4 |
| 30367 | 0.49 | 2.9E-02 | 41 | THBS1 | thrombospondin 1 |
| 120 | 0.49 | 4.9E-05 | 41 | CCN3 | cellular communication network factor 3 |
| 302 | 0.50 | 1.3E-07 | 41 | AGT | angiotensinogen |
| 2416 | 0.57 | 2.6E-05 | 48 | ANGPTL4 | angiopoietin like 4 |
| 192 | 1.16 | 4.5E-11 | 123 | GREM1 | gremlin 1, DAN family BMP antagonist |
| 15 | 1.16 | 7.0E-04 | 123 | LAMA3 | laminin subunit alpha 3 |
| 71 | 1.22 | 2.4E-05 | 133 | ANGPT2 | angiopoietin 2 |

***Table 5****: DEGs from autophagy (GO:0006914) pathway significantly modified after 24h treatment with 50 µg/mL of B. serrata extract. Blue/red color according to log2FoldChange. baseMean : mean count, padj :adjusted p-value, FC fold change in % compared to the control.*

| **baseMean** | **log2FoldChange** | | | **padj** | | **FC (%)** | | **Symbol** | **GeneName** | | |
| --- | --- | --- | --- | --- | --- | --- | --- | --- | --- | --- | --- |
| 24.5 | | 1.27 | 1.71E-05 | | +140 | | TRIM17 | | | tripartite motif containing 17 |  |
| 1700.4 | | 0.69 | 1.76E-35 | | +61 | | PLEKHM1 | | | pleckstrin homology and RUN domain containing M1 |  |
| 44323.5 | | 0.62 | 2.15E-08 | | +54 | | SQSTM1 | | | sequestosome 1 |  |
| 3820.6 | | 0.60 | 5.45E-38 | | +52 | | NPC1 | | | NPC intracellular cholesterol transporter 1 |  |
| 515.4 | | 0.55 | 5.22E-06 | | +46 | | RUBCNL | | | rubicon like autophagy enhancer |  |
| 1069.6 | | 0.48 | 3.31E-10 | | +39 | | RETREG1 | | | reticulophagy regulator 1 |  |
| 556.9 | | 0.39 | 1.00E-08 | | +31 | | GRAMD1A | | | GRAM domain containing 1A |  |
| 4537.3 | | 0.35 | 4.72E-20 | | +28 | | RAB7A | | | RAB7A, member RAS oncogene family |  |
| 447.5 | | 0.35 | 5.13E-07 | | +27 | | FOXO1 | | | forkhead box O1 |  |
| 177.2 | | 0.35 | 1.98E-03 | | +27 | | C9orf72 | | | C9orf72-SMCR8 complex subunit |  |
| 714.2 | | 0.33 | 3.07E-09 | | +25 | | WIPI1 | | | WD repeat domain, phosphoinositide interacting 1 |  |
| 924.0 | | 0.32 | 7.07E-08 | | +25 | | CHMP1B | | | charged multivesicular body protein 1B |  |
| 1389.3 | | 0.32 | 1.76E-17 | | +24 | | TOLLIP | | | toll interacting protein |  |
| 484.7 | | 0.30 | 8.66E-07 | | +23 | | SIRT2 | | | sirtuin 2 |  |
| 606.5 | | 0.30 | 8.25E-07 | | +23 | | CHMP2B | | | charged multivesicular body protein 2B |  |
| 500.9 | | 0.30 | 6.82E-10 | | +23 | | RMC1 | | | regulator of MON1-CCZ1 |  |
| 2079.4 | | 0.30 | 1.42E-04 | | +22 | | MAP1LC3B | | | microtubule associated protein 1 light chain 3 beta |  |
| 356.1 | | 0.29 | 1.77E-07 | | +22 | | UVRAG | | | UV radiation resistance associated |  |
| 1079.8 | | 0.28 | 1.90E-04 | | +21 | | ATG2A | | | autophagy related 2A |  |
| 272.8 | | 0.27 | 2.27E-04 | | +20 | | ATG14 | | | autophagy related 14 |  |
| 1941.2 | | 0.26 | 1.00E-13 | | +20 | | LAMP2 | | | lysosomal associated membrane protein 2 |  |
| 588.0 | | -0.34 | 5.32E-08 | | -20 | | TP53INP2 | | | tumor protein p53 inducible nuclear protein 2 |  |
| 45.8 | | -0.83 | 1.62E-07 | | -44 | | EVA1A | | | eva-1 homolog A, regulator of programmed cell death |  |
| 2744.3 | | -0.93 | 3.83E-28 | | -48 | | DEPP1 | | | DEPP1 autophagy regulator |  |
