# Supplementary material for: Curcuma longa and Boswellia serrata Extracts Modulate Different and Complementary Pathways on Human Chondrocytes In Vitro: Deciphering of a Transcriptomic Study
Source: Front Pharmacol. 2022 Aug 11;13:931914. doi: 10.3389/fphar.2022.931914 (PMC9403192; doi:10.3389/fphar.2022.931914)
Supplement: Supplementary file 9 [file DataSheet8.DOCX]

***Table 1****:* *DEGs from Nrf2 pathways significantly modified after 24h treatment with 2µg/mL of C. longa extract. Red color according to log2FoldChange. baseMean : mean count, padj :adjusted p-value.*

| **Role** | **baseMean** | **log2**  **FoldChange** | **Variation** | **padj** | **Symbol** | **GeneName** |  |  |  |  |
| --- | --- | --- | --- | --- | --- | --- | --- | --- | --- | --- |
| Detoxification | 16 | 3.38 | x 10 | 3.6E-15 | AKR1B10 | aldo-keto reductase family 1 member B10 |  |  |  |  |
| Phase I | 34 | 0.84 | +79% | 7.9E-05 | AKR1B15 | aldo-keto reductase family 1 member B15 |  |  |  |  |
|  | 11336 | 0.54 | +46% | 1.3E-06 | AKR1C1 | aldo-keto reductase family 1 member C1 |  |  |  |  |
|  | 2338 | 0.75 | +68% | 4.1E-69 | ALDH3A2 | aldehyde dehydrogenase 3 family member A2 |  |  |  |  |
|  | 664 | 0.63 | +55% | 1.0E-13 | CBR1 | carbonyl reductase 1 | | |  |  |
|  | 483 | 1.42 | x 2.7 | 8.5E-50 | EPHX1 | epoxide hydrolase 1 | | |  |  |
|  | 3346 | 1.84 | x 3.6 | 3.6E-144 | NQO1 | NAD(P)H quinone dehydrogenase 1 | | | |  |
|  | 896 | 0.52 | +44% | 1.1E-15 | NQO2 | N-ribosyldihydronicotinamide:quinone reductase 2 |  |  |  |  |
|  |  |  |  |  |  |  | |  |  |  |
| Detoxification | 23 | 1.89 | x 3.7 | 6.4E-11 | ABCB6 | ATP binding cassette subfamily B member 6 |  |  |  |  |
| Phase III | 6 | 2.06 | x 4.2 | 6.1E-06 | SLCO2B1 | solute carrier organic anion transporter 2B1 |  |  |  |  |
|  |  |  |  |  |  |  | |  |  |  |
| Anti-oxidant | 459 | 1.06 | x 2.1 | 3.6E-21 | GCLC | glutamate-cysteine ligase catalytic subunit |  |  |  |  |
| GSH-based | 1436 | 1.58 | x 3 | 3.5E-43 | GCLM | glutamate-cysteine ligase modifier subunit |  |  |  |  |
|  | 8 | 1.03 | x 2 | 2.8E-03 | GGT1 | gamma-glutamyltransferase 1 | | | |  |
|  | 2128 | 0.29 | +22% | 1.3E-05 | GLRX | glutaredoxin | | |  |  |
|  | 238 | 0.26 | +20% | 2.2E-04 | GLRX2 | glutaredoxin 2 | | |  |  |
|  | 855 | 0.49 | +40% | 2.0E-18 | GLRX3 | glutaredoxin 3 | | |  |  |
|  | 1955 | 0.27 | +20% | 2.6E-08 | GLS | glutaminase | | |  |  |
|  | 783 | 1.23 | x 2.3 | 1.9E-23 | GSR | glutathione-disulfide reductase | | | |  |
|  | 342 | 0.36 | +28% | 1.1E-08 | GSTM3 | glutathione S-transferase mu 3 | | | |  |
|  | 73 | 0.34 | +27% | 1.6E-02 | GSTM5 | glutathione S-transferase mu 5 | | | |  |
|  | 640 | 0.41 | +33% | 4.0E-12 | MGST1 | microsomal glutathione S-transferase 1 |  |  |  |  |
|  | 1851 | 1.22 | x 2.3 | 4.8E-18 | SLC7A11 | cysteine/glutamate transporter | | | |  |
|  |  |  |  |  |  |  | |  |  |  |
| Anti-oxidant | 5363 | 0.39 | +31% | 3.5E-21 | PRDX1 | peroxiredoxin 1 | | |  |  |
| TXN-based | 16 | 2.25 | x 4.8 | 4.3E-12 | SRXN1 | sulfiredoxin 1 | | |  |  |
|  | 2763 | 0.70 | +62% | 9.9E-47 | TXN | thioredoxin | |  |  |  |
|  | 5086 | 1.42 | x2.7 | 1.4E-36 | TXNRD1 | thioredoxin reductase 1 | | |  |  |
|  |  |  |  |  |  |  | |  |  |  |
| NADPH | 892 | 0.43 | +34% | 7.3E-20 | G6PD | glucose-6-phosphate dehydrogenase | | | |  |
| regeneration | 614 | 0.31 | +24% | 1.3E-04 | IDH1 | isocitrate dehydrogenase (NADP(+)) 1 | | | |  |
|  | 478 | 1.24 | x 2.4 | 1.5E-37 | ME1 | malic enzyme 1 | | |  |  |
|  | 2134 | 0.99 | x 2 | 1.4E-45 | PGD | phosphogluconate dehydrogenase | | | |  |
|  | 2918 | 0.51 | +42% | 8.4E-21 | TALDO1 | transaldolase 1 | | |  |  |
|  | 3273 | 0.97 | +95% | 6.0E-71 | TKT | transketolase | | |  |  |
|  | 6151 | 0.90 | +87% | 4.4E-41 | UGDH | UDP-glucose 6-dehydrogenase | | | |  |
|  |  |  |  |  |  |  | |  |  |  |
| Heme and iron | 348 | 0.48 | +40% | 5.4E-09 | BLVRB | biliverdin reductase B | | |  |  |
| metabolism | 222 | 0.49 | +40% | 1.4E-12 | FECH | ferrochelatase | | |  |  |
|  | 138641 | 0.94 | +92% | 3.1E-40 | FTH1 | ferritin heavy chain 1 | | |  |  |
|  | 56001 | 1.43 | x 2.7 | 1.2E-32 | FTL | ferritin light chain | | |  |  |
|  | 49379 | 3.82 | x 14.1 | 4.0E-54 | HMOX1 | heme oxygenase 1 | | |  |  |
|  | 318 | 0.44 | +36% | 9.5E-09 | SLC48A1 | heme transporter | | |  |  |
|  |  |  |  |  |  |  | |  |  |  |
| metabolism | 132 | 0.37 | +29% | 1.3E-04 | PPAT | phosphoribosyl pyrophosphate amidotransferase |  |  |  |  |
| other | 1079 | 0.86 | +81% | 3.1E-40 | SLC6A6 | taurine transporter, anti-oxidant | | | |  |
|  |  |  |  |  |  |  | |  |  |  |
| Chaperone / | 2893 | 0.66 | +58% | 4.6E-36 | BAG3 | BAG cochaperone 3 | | |  |  |
| stress | 325 | 0.50 | +41% | 2.8E-06 | DNAJA4 | DnaJ heat shock protein family (Hsp40) member A4 |  |  |  |  |
| proteins | 1741 | 0.84 | +79% | 1.1E-07 | DNAJB1 | DnaJ heat shock protein family (Hsp40) member B1 |  |  |  |  |
|  | 362 | 1.14 | x 2.2 | 3.1E-13 | DNAJB4 | DnaJ heat shock protein family (Hsp40) member B4 |  |  |  |  |
|  | 1402 | 0.24 | +18% | 5.2E-05 | DNAJB6 | DnaJ heat shock protein family (Hsp40) member B6 |  |  |  |  |
|  | 366 | 0.54 | +46% | 4.4E-10 | DNAJC2 | DnaJ heat shock protein family (Hsp40) member C2 |  |  |  |  |
|  | 271 | 1.44 | x 2.7 | 6.5E-19 | DNAJC6 | DnaJ heat shock protein family (Hsp40) member C6 |  |  |  |  |
|  | 16698 | 0.78 | +72% | 1.0E-07 | HSP90AA1 | heat shock protein 90 alpha family class A member 1 |  |  |  |  |
|  | 11793 | 0.25 | +19% | 2.5E-03 | HSP90AB1 | heat shock protein 90 alpha family class B member 1 |  |  |  |  |
|  | 150 | 0.49 | +40% | 1.9E-06 | HSPA12A | heat shock protein family A (Hsp70) member 12A |  |  |  |  |
|  | 1298 | 1.73 | x 3.3 | 1.3E-11 | HSPA1A | heat shock protein family A (Hsp70) member 1A |  |  |  |  |
|  | 784 | 1.56 | x 3 | 1.3E-10 | HSPA1B | heat shock protein family A (Hsp70) member 1B |  |  |  |  |
|  | 601 | 0.69 | +61% | 1.7E-20 | HSPA2 | heat shock protein family A (Hsp70) member 2 |  |  |  |  |
|  | 2001 | 0.19 | +14% | 4.3E-06 | HSPA4 | heat shock protein family A (Hsp70) member 4 |  |  |  |  |
|  | 142 | 0.45 | +37% | 5.5E-05 | HSPA4L | heat shock protein family A (Hsp70) member 4 like |  |  |  |  |
|  | 195 | 3.11 | x 8.6 | 3.6E-15 | HSPA6 | heat shock protein family A (Hsp70) member 6 |  |  |  |  |
|  | 14294 | 0.58 | +50% | 1.1E-05 | HSPA8 | heat shock protein family A (Hsp70) member 8 |  |  |  |  |
|  | 5928 | 0.27 | +20% | 1.5E-05 | HSPA9 | heat shock protein family A (Hsp70) member 9 |  |  |  |  |
|  | 928 | 0.44 | +35% | 1.3E-05 | HSPB1 | heat shock protein family B (small) member 1 |  |  |  |  |
|  | 423 | 1.61 | x 3 | 9.0E-22 | HSPB8 | heat shock protein family B (small) member 8 |  |  |  |  |
|  | 1653 | 0.84 | +79% | 6.4E-18 | MAFG | MAF bZIP transcription factor G | | | |  |
|  | 2178 | 0.35 | +28% | 2.4E-06 | STIP1 | stress induced phosphoprotein 1 |  |  |  |  |
|  |  |  |  |  |  |  | |  |  |  |
| Autophagy | 516 | 0.26 | +20% | 2.0E-08 | AMBRA1 | autophagy and beclin 1 regulator 1 | | | |  |
| proteostasis | 129 | 0.18 | +13% | 2.8E-02 | ATG4C | autophagy related 4C cysteine peptidase |  |  |  |  |
|  | 341 | 0.23 | +18% | 5.7E-05 | ATG5 | autophagy related 5 | | |  |  |
|  | 1358 | 1.03 | x 2 | 2.1E-26 | FBXO30 | F-box protein 30 | | | |  |
|  | 1077 | 0.81 | +74% | 2.6E-21 | GABARAPL1 | GABA type A receptor associated protein like 1 |  |  |  |  |
|  | 1450 | 0.45 | +36% | 9.4E-12 | KEAP1 | kelch like ECH associated protein 1 | | | |  |
|  | 1225 | 0.53 | +44% | 6.5E-10 | PSMB2 | proteasome 20S subunit beta 2 | | | |  |
|  | 1054 | 0.33 | +26% | 1.3E-05 | PSMB5 | proteasome 20S subunit beta 5 | | | |  |
|  | 1744 | 0.21 | +16% | 1.2E-04 | PSMB1 | proteasome 20S subunit beta 1 | | | |  |
|  | 1372 | 0.26 | +20% | 1.9E-04 | PSMB6 | proteasome 20S subunit beta 6 | | | |  |
|  | 2516 | 0.24 | +18% | 4.1E-04 | PSMB4 | proteasome 20S subunit beta 4 | | | |  |
|  | 814 | 0.21 | +16% | 1.1E-02 | PSMB3 | proteasome 20S subunit beta 3 | | | |  |
|  | 44323 | 1.39 | x 2.6 | 8.3E-41 | SQSTM1 | sequestosome 1 | | |  |  |
|  | 5257 | 0.21 | +16% | 1.6E-02 | UBB | ubiquitin B | |  |  |  |
|  | 819 | 0.54 | +45% | 4.0E-08 | ULK1 | unc-51 like autophagy activating kinase 1 |  |  |  |  |
|  | 1182 | 0.52 | +44% | 1.5E-10 | USP14 | ubiquitin specific peptidase 14 | | | |  |
|  | 913 | 0.38 | +30% | 1.6E-13 | WIPI2 | WD repeat domain, phosphoinositide interacting 2 |  |  |  |  |

***Table 2****:* *DEGs from « Inflammatory response GO :0006954 » pathways significantly modified after 24h treatment with 2µg/mL of C. longa extract. Red color according to log2FoldChange. baseMean : mean count, padj :adjusted p-value.*

| **baseMean** | **log2FoldChange** | **inhibition** | **padj** | **Symbol** | **GeneName** |
| --- | --- | --- | --- | --- | --- |
| 984 | -2.76 | 85.2% | 3.0E-16 | CXCL6 | C-X-C motif chemokine ligand 6 |
| 3195 | -2.49 | 82.3% | 2.7E-14 | CXCL1 | C-X-C motif chemokine ligand 1 |
| 3084 | -2.43 | 81.5% | 3.4E-26 | IL6 | interleukin 6 |
| 28 | -2.37 | 80.6% | 1.9E-25 | GGT5 | gamma-glutamyltransferase 5 |
| 194 | -2.21 | 78.3% | 4.3E-15 | IL1B | interleukin 1 beta |
| 10 | -2.16 | 77.7% | 2.1E-09 | CCL7 | C-C motif chemokine ligand 7 |
| 349 | -2.11 | 76.8% | 2.3E-22 | CXCL5 | C-X-C motif chemokine ligand 5 |
| 772 | -2.06 | 76.1% | 6.7E-60 | IGFBP4 | insulin like growth factor binding protein 4 |
| 311 | -1.97 | 74.5% | 3.6E-20 | CXCL3 | C-X-C motif chemokine ligand 3 |
| 328 | -1.95 | 74.1% | 4.0E-14 | CCL2 | C-C motif chemokine ligand 2 |
| 102 | 1.93 | 73.8% | 1.1E-35 | POSTN | periostin |
| 112 | -1.90 | 73.2% | 6.6E-12 | SELE | selectin E |
| 505 | -1.87 | 72.6% | 5.4E-14 | CXCL2 | C-X-C motif chemokine ligand 2 |
| 132 | -1.75 | 70.4% | 5.8E-27 | CX3CL1 | C-X3-C motif chemokine ligand 1 |
| 19 | -1.70 | 69.3% | 7.6E-10 | SLC11A1 | solute carrier family 11 member 1 |
| 6280 | -1.65 | 68.0% | 3.6E-09 | NOS2 | nitric oxide synthase 2 |
| 20 | -1.53 | 65.3% | 1.0E-06 | TNF | tumor necrosis factor |
| 4666 | -1.45 | 63.5% | 7.5E-25 | MSMP | microseminoprotein, prostate associated |
| 171 | -1.45 | 63.5% | 1.6E-12 | IL1RN | interleukin 1 receptor antagonist |
| 10 | -1.44 | 63.1% | 2.7E-02 | IL1A | interleukin 1 alpha |
| 404 | -1.44 | 63.1% | 3.0E-18 | TNFRSF1B | TNF receptor superfamily member 1B |
| 88 | -1.38 | 61.6% | 2.1E-05 | CCL3 | C-C motif chemokine ligand 3 |
| 48788 | -1.35 | 60.8% | 1.2E-13 | CXCL8 | C-X-C motif chemokine ligand 8 |
| 2054 | -1.32 | 60.1% | 2.9E-09 | TLR2 | toll like receptor 2 |
| 1064 | -1.29 | 59.2% | 1.7E-24 | LOXL3 | lysyl oxidase like 3 |
| 18672 | -1.27 | 58.5% | 1.2E-09 | CCL20 | C-C motif chemokine ligand 20 |
| 42 | -1.26 | 58.3% | 5.9E-14 | C3AR1 | complement C3a receptor 1 |
| 41 | -1.22 | 57.1% | 2.7E-04 | CCL4 | C-C motif chemokine ligand 4 |
| 11 | -1.20 | 56.5% | 2.9E-05 | ADORA2A | adenosine A2a receptor |
| 5 | -1.18 | 55.9% | 1.8E-02 | TRIL | TLR4 interactor with leucine rich repeats |
| 122613 | -1.17 | 55.6% | 2.2E-51 | CHI3L1 | chitinase 3 like 1 |
| 302 | -1.17 | 55.6% | 4.6E-09 | GPR68 | G protein-coupled receptor 68 |
| 6 | -1.16 | 55.2% | 9.7E-03 | IL34 | interleukin 34 |
| 224 | -1.13 | 54.2% | 2.0E-22 | STAB1 | stabilin 1 |
| 141 | -1.12 | 54.1% | 2.9E-30 | ITGB2 | integrin subunit beta 2 |
| 1666 | -1.12 | 54.1% | 1.5E-18 | MGLL | monoglyceride lipase |
| 33 | -1.10 | 53.2% | 3.8E-10 | IL32 | interleukin 32 |
| 6711 | -1.05 | 51.7% | 3.6E-21 | HSPG2 | heparan sulfate proteoglycan 2 |
| 54 | -1.04 | 51.2% | 5.0E-09 | TNFSF10 | TNF superfamily member 10 |
| 1176 | -1.03 | 50.9% | 5.7E-19 | TNFAIP3 | TNF alpha induced protein 3 |
| 13 | -1.01 | 50.2% | 3.2E-03 | IL36G | interleukin 36 gamma |
| 10466 | -1.00 | 50.0% | 1.2E-05 | PTGS2 | prostaglandin-endoperoxide synthase 2 |
| 509 | -0.97 | 49.0% | 1.8E-12 | BDKRB2 | bradykinin receptor B2 |
| 123 | -0.94 | 47.9% | 2.4E-19 | PTGFR | prostaglandin F receptor |
| 4803 | -0.93 | 47.4% | 6.2E-08 | TNFAIP6 | TNF alpha induced protein 6 |
| 6489 | -0.93 | 47.4% | 5.3E-18 | FSTL1 | Follistatin like 1 |
| 6 | -0.90 | 46.6% | 1.7E-02 | CXCL10 | C-X-C motif chemokine ligand 10 |
| 181 | -0.90 | 46.6% | 1.1E-08 | PTGER4 | prostaglandin E receptor 4 |
| 97 | -0.89 | 46.2% | 7.2E-08 | IL36RN | interleukin 36 receptor antagonist |
| 36 | -0.89 | 46.0% | 1.6E-04 | C5AR1 | complement C5a receptor 1 |
| 1606 | -0.82 | 43.5% | 7.8E-11 | PTX3 | pentraxin 3 |
| 28101 | -0.82 | 43.3% | 4.5E-06 | CSF1 | colony stimulating factor 1 |
| 59 | -0.80 | 42.6% | 2.3E-10 | PTGIR | prostaglandin I2 receptor |
| 1015 | -0.77 | 41.5% | 2.0E-08 | SCUBE1 | signal peptide, CUB domain and EGF like domain containing 1 |
| 233 | -0.76 | 41.0% | 3.3E-09 | PSTPIP1 | proline-serine-threonine phosphatase interacting protein 1 |
| 1583 | -0.76 | 40.9% | 5.2E-11 | CMKLR1 | chemerin chemokine-like receptor 1 |
| 95 | -0.74 | 40.3% | 3.6E-12 | LYZ | lysozyme |
| 535 | -0.73 | 39.6% | 3.5E-09 | VNN1 | vanin 1 |
| 1648 | -0.72 | 39.4% | 7.9E-35 | C3 | complement C3 |
| 1790 | -0.71 | 38.8% | 1.7E-18 | AXL | AXL receptor tyrosine kinase |
| 3249 | -0.69 | 37.9% | 2.7E-17 | ADM | adrenomedullin |
| 42937 | -0.65 | 36.4% | 3.8E-08 | BMP2 | bone morphogenetic protein 2 |
| 70 | -0.65 | 36.3% | 4.5E-07 | C4A | complement C4A (Rodgers blood group) |
| 4354 | -0.64 | 36.0% | 6.6E-19 | TNIP1 | TNFAIP3 interacting protein 1 |
| 46 | -0.63 | 35.6% | 3.4E-03 | AFAP1L2 | actin filament associated protein 1 like 2 |
| 49 | -0.63 | 35.2% | 1.6E-02 | SERPINA3 | serpin family A member 3 |
| 396 | -0.62 | 35.0% | 3.5E-04 | ADORA1 | adenosine A1 receptor |
| 115 | -0.62 | 34.9% | 5.3E-06 | BDKRB1 | bradykinin receptor B1 |
| 806 | -0.61 | 34.7% | 4.7E-09 | CHST2 | carbohydrate sulfotransferase 2 |
| 50 | -0.61 | 34.3% | 2.3E-04 | S100A9 | S100 calcium binding protein A9 |
| 392 | -0.61 | 34.7% | 1.3E-13 | IL16 | Interleukin 16 |
| 67 | -0.60 | 33.8% | 3.8E-07 | TRPV1 | transient receptor potential cation channel subfamily V member 1 |
| 767 | -0.59 | 33.5% | 1.5E-17 | CYBA | cytochrome b-245 alpha chain |
| 35 | -0.58 | 33.3% | 1.3E-03 | IL17RE | interleukin 17 receptor E |
| 1606 | -0.58 | 32.9% | 3.4E-14 | ZC3H12A | zinc finger CCCH-type containing 12A |
| 13 | -0.58 | 32.9% | 3.5E-02 | TNFSF4 | TNF superfamily member 4 |
| 110 | -0.57 | 32.5% | 2.0E-05 | IL36B | interleukin 36 beta |
| 41 | -0.56 | 32.4% | 1.4E-04 | AGER | advanced glycosylation end-product specific receptor |
| 666 | -0.56 | 32.2% | 1.3E-15 | IL17RC | interleukin 17 receptor C |
| 117 | -0.54 | 31.3% | 1.7E-07 | SEMA7A | semaphorin 7A (John Milton Hagen blood group) |
| 210 | -0.53 | 30.8% | 8.9E-12 | HYAL1 | hyaluronidase 1 |
| 163 | -0.51 | 29.6% | 1.4E-09 | PLGRKT | plasminogen receptor with a C-terminal lysine |
| 1375 | -0.50 | 29.5% | 1.7E-13 | TCIRG1 | T cell immune regulator 1, ATPase H+ transporting V0 subunit a3 |
| 1145 | -0.50 | 29.4% | 1.1E-16 | NFKB1 | nuclear factor kappa B subunit 1 |
| 75 | -0.50 | 29.4% | 1.4E-03 | PYCARD | PYD and CARD domain containing |
| 109 | -0.50 | 29.3% | 1.5E-03 | PTGS1 | prostaglandin-endoperoxide synthase 1 |
| 879 | -0.49 | 28.7% | 3.7E-16 | RELB | RELB proto-oncogene, NF-kB subunit |
| 123789 | -0.47 | 27.6% | 1.9E-05 | SPP1 | secreted phosphoprotein 1 |
| 1238 | -0.46 | 27.5% | 1.9E-20 | MAP2K3 | mitogen-activated protein kinase kinase 3 |
| 11182 | -0.44 | 26.5% | 4.3E-14 | CD44 | CD44 molecule (Indian blood group) |
| 3077 | -0.43 | 25.8% | 4.1E-09 | NFKBIZ | NFKB inhibitor zeta |
| 56 | -0.43 | 25.7% | 3.7E-03 | NFATC4 | nuclear factor of activated T cells 4 |
| 2111 | -0.42 | 25.2% | 6.2E-14 | PRDX5 | peroxiredoxin 5 |
| 758 | -0.41 | 24.6% | 3.3E-05 | CD14 | CD14 molecule |
| 1821 | -0.41 | 24.6% | 2.0E-14 | TRAF3IP2 | TRAF3 interacting protein 2 |
| 483 | -0.40 | 24.3% | 1.4E-03 | IL17RB | interleukin 17 receptor B |
| 1378 | -0.37 | 22.5% | 2.6E-07 | TGFB1 | transforming growth factor beta 1 |
| 367 | -0.37 | 22.4% | 1.5E-04 | ADGRE5 | adhesion G protein-coupled receptor E5 |
| 1861 | -0.36 | 22.3% | 3.1E-08 | IRAK2 | interleukin 1 receptor associated kinase 2 |
| 615 | -0.36 | 22.0% | 2.9E-02 | AOC3 | amine oxidase copper containing 3 |
| 438 | -0.35 | 21.4% | 1.1E-07 | HDAC4 | histone deacetylase 4 |
| 46 | -0.33 | 20.7% | 2.2E-02 | C4B | complement C4B (Chido blood group) |
| 556 | -0.33 | 20.3% | 1.2E-05 | GSDMD | gasdermin D |
| 744 | -0.33 | 20.2% | 1.9E-04 | MIF | macrophage migration inhibitory factor |
| 54 | -0.32 | 20.0% | 1.7E-02 | LTB4R | leukotriene B4 receptor |
| 465 | -0.32 | 19.8% | 4.8E-03 | LACC1 | laccase domain containing 1 |
| 1438 | -0.31 | 19.2% | 1.2E-07 | EPHA2 | EPH receptor A2 |
| 529 | -0.30 | 18.7% | 1.3E-06 | TICAM1 | toll like receptor adaptor molecule 1 |
| 1905 | -0.29 | 17.9% | 5.3E-06 | PLA2G2A | phospholipase A2 group IIA |
| 210 | -0.27 | 17.1% | 4.4E-05 | PIK3CD | phosphatidylinositol-4,5-bisphosphate 3-kinase catalytic subunit delta |
| 1133 | -0.25 | 16.1% | 1.5E-03 | PTGER2 | prostaglandin E receptor 2 |

***Table 3****: DEGs involved in extracellular matrix degradation significantly modified after 24h treatment with 2µg/mL of C. longa extract. Blue/red color according to log2FoldChange. baseMean : mean count, padj :adjusted p-value.*

| **baseMean** | **log2FoldChange %** | | **padj** | **Symbol** | **GeneName** |  |
| --- | --- | --- | --- | --- | --- | --- |
| 8 | -1.27 | - 58.4% | 8.2E-04 | MMP12 | matrix metallopeptidase 12 | |
| 160 | -1.19 | - 56.1% | 4.1E-30 | ADAMTSL4 | ADAMTS like 4 | |
| 20155 | -1.16 | - 55.1% | 3.5E-29 | MMP13 | matrix metallopeptidase 13 | |
| 40 | -0.99 | - 49.5% | 6.6E-06 | ADAMTS4 | ADAM metallopeptidase with thrombospondin motif 4 | |
| 1711 | -0.98 | - 49.3% | 7.8E-07 | ADAMTS9 | ADAM metallopeptidase with thrombospondin motif 9 | |
| 693 | -0.81 | - 43.1% | 7.9E-24 | CTSS | cathepsin S |  |
| 649 | -0.79 | - 42.3% | 1.3E-14 | ADAMTS5 | ADAM metallopeptidase with thrombospondin motif 5 | |
| 100521 | -0.77 | - 41.4% | 7.2E-11 | MMP1 | matrix metallopeptidase 1 | |
| 197054 | -0.76 | - 41.1% | 3.1E-18 | SERPINE2 | serpin family E member 2 | |
| 20159 | -0.68 | - 37.6% | 8.7E-19 | MMP14 | matrix metallopeptidase 14 | |
| 795 | -0.64 | - 35.9% | 1.7E-22 | CTSC | cathepsin C |  |
| 49 | -0.63 | - 35.2% | 1.6E-02 | SERPINA3 | serpin family A member 3 | |
| 176 | -0.60 | - 34.0% | 5.0E-07 | ADAMTS10 | ADAM metallopeptidase with thrombospondin motif 10 | |
| 87 | -0.59 | - 33.5% | 2.9E-06 | ADAMTS2 | ADAM metallopeptidase with thrombospondin motif 2 | |
| 1353040 | -0.57 | - 32.6% | 1.9E-33 | MMP3 | matrix metallopeptidase 3 | |
| 615 | -0.56 | - 32.3% | 1.2E-05 | MMP10 | matrix metallopeptidase 10 | |
| 20 | -0.54 | - 31.0% | 2.0E-02 | ADAMTS3 | ADAM metallopeptidase with thrombospondin motif 3 | |
| 14508 | -0.49 | - 28.8% | 6.9E-10 | TIMP2 | TIMP metallopeptidase inhibitor 2 | |
| 2606 | -0.46 | - 27.2% | 9.8E-13 | HTRA1 | HtrA serine peptidase 1 | |
| 59254 | -0.35 | - 21.8% | 2.5E-16 | SERPINA1 | serpin family A member 1 | |
| 677 | -0.34 | - 20.9% | 7.3E-06 | SERPINA5 | serpin family A member 5 | |
| 1965 | -0.34 | - 20.8% | 1.4E-15 | SERPING1 | serpin family G member 1 | |
| 1586 | -0.32 | - 20.0% | 2.1E-13 | MMP2 | matrix metallopeptidase 2 | |
| 6800 | -0.32 | - 19.8% | 2.5E-02 | TIMP3 | TIMP metallopeptidase inhibitor 3 | |
| 646 | -0.28 | - 17.9% | 9.5E-06 | SERPINB1 | serpin family B member 1 | |
| 1413 | -0.28 | - 17.6% | 5.3E-03 | ADAMTS1 | ADAM metallopeptidase with thrombospondin motif 1 | |
| 253 | 2.32 | X 5 | 2.8E-78 | HTRA3 | HtrA serine peptidase 3 | |

***Table 4****: DEGs from cartilage development and endochondral ossification pathways significantly modified after 24h treatment with 2µg/mL of C. longa extract. Blue/red color according to log2FoldChange. baseMean : mean count, padj :adjusted p-value.*

| **baseMean** | **log2FoldChange** | **Change** | **padj** | **Symbol** | **GeneName** |  |
| --- | --- | --- | --- | --- | --- | --- |
| 88 | -1.97 | -74.6% | 3.2E-14 | LRRC15 | leucine rich repeat containing 15 | |
| 1727 | -1.54 | -65.5% | 5.8E-24 | TNXB | tenascin XB |  |
| 120 | -1.52 | -65.2% | 3.7E-36 | VCAN | versican |  |
| 865 | -1.46 | -64.6% | 8.0E-14 | **FGF1** | fibroblast growth factor 1 |  |
| 515 | -1.45 | -63.4% | 4.4E-16 | NDP | norrin cystine knot growth factor NDP | |
| 11471 | -1.45 | -63.4% | 3.5E-54 | TNC | tenascin C |  |
| 18 | -1.42 | -62.6% | 9.3E-07 | FGF10 | fibroblast growth factor 10 | |
| 52 | -1.39 | -61.9% | 2.8E-15 | SFRP1 | secreted frizzled related protein 1 | |
| 286 | -1.38 | -61.7% | 4.4E-30 | LAMB1 | laminin subunit beta 1 | |
| 35 | -1.37 | -61.4% | 5.9E-06 | COL5A3 | collagen type V alpha 3 chain | |
| 230 | -1.30 | - 59.4% | 1.2E-14 | WNT5A | Wnt family member 5A | |
| 28411 | -1.28 | - 58.9% | 3.8E-17 | TGM2 | transglutaminase 2 | |
| 94 | -1.25 | - 58.0% | 1.3E-18 | FBN2 | fibrillin 2 |  |
| 689 | -1.16 | - 55.4% | 1.4E-34 | AGRN | agrin |  |
| 4641 | -1.16 | - 55.2% | 3.1E-15 | COL15A1 | collagen type XV alpha 1 chain | |
| 6711 | -1.05 | - 51.7% | 3.6E-21 | HSPG2 | heparan sulfate proteoglycan 2 | |
| 411 | -1.05 | - 51.7% | 5.0E-36 | COL16A1 | collagen type XVI alpha 1 chain | |
| 1512 | -1.03 | - 50.9% | 5.2E-20 | COL5A1 | collagen type V alpha 1 chain | |
| 9202 | -1.00 | - 50.1% | 5.9E-18 | LTBP2 | latent TGF beta binding protein 2 | |
| 609 | -0.98 | - 49.2% | 1.8E-25 | MATN3 | matrilin 3 |  |
| 3884 | -0.98 | - 49.1% | 8.1E-43 | AEBP1 | AE binding protein 1 | |
| 1994 | -0.90 | - 46.3% | 2.7E-20 | COL12A1 | collagen type XII alpha 1 chain | |
| 10734 | -0.89 | - 46.2% | 7.0E-18 | COL3A1 | collagen type III alpha 1 chain | |
| 1511 | -0.87 | - 45.2% | 5.4E-33 | SRPX2 | sushi repeat containing protein X-linked 2 | |
| 416 | -0.86 | - 45.0% | 3.0E-18 | PODNL1 | podocan like 1 | |
| 74 | -0.86 | - 44.9% | 1.8E-06 | GPC4 | glypican 4 |  |
| 130 | -0.83 | - 43.9% | 4.0E-19 | NID2 | nidogen 2 |  |
| 3285 | -0.82 | - 43.5% | 2.0E-08 | F13A1 | coagulation factor XIII A chain | |
| 358 | -0.82 | - 43.3% | 4.2E-13 | COL14A1 | collagen type XIV alpha 1 chain | |
| 160917 | -0.82 | - 43.3% | 6.9E-10 | ACAN | aggrecan |  |
| 1524 | -0.81 | - 43.1% | 4.7E-20 | SMOC2 | SPARC related modular calcium binding 2 | |
| 4659 | -0.81 | - 43.0% | 2.7E-12 | COL11A1 | collagen type XI alpha 1 chain | |
| 42 | -0.81 | - 43.0% | 1.6E-04 | GDF10 | growth differentiation factor 10 | |
| 2564 | -0.80 | - 42.8% | 1.5E-18 | GPC6 | glypican 6 |  |
| 127 | -0.79 | - 42.2% | 4.0E-13 | SPON1 | spondin 1 |  |
| 115 | -0.78 | - 41.8% | 8.0E-14 | TGFB3 | transforming growth factor beta 3 | |
| 344 | -0.78 | - 41.8% | 1.2E-08 | THSD4 | thrombospondin type 1 domain containing 4 | |
| 10 | -0.76 | - 40.8% | 2.1E-02 | ASPN | asporin |  |
| 66 | -0.75 | - 40.5% | 2.5E-07 | CDH13 | cadherin 13 |  |
| 641 | -0.74 | - 40.3% | 2.4E-36 | EFEMP1 | EGF containing fibulin extracellular matrix protein 1 | |
| 28021 | -0.74 | - 40.1% | 1.2E-08 | COL6A3 | collagen type VI alpha 3 chain | |
| 192 | -0.70 | - 38.4% | 2.5E-10 | THBS2 | thrombospondin 2 | |
| 1047 | -0.70 | - 38.3% | 3.6E-43 | EFEMP2 | EGF containing fibulin extracellular matrix protein 2 | |
| 5358 | -0.70 | - 38.2% | 9.5E-13 | SCARA3 | scavenger receptor class A member 3 | |
| 9693 | -0.67 | - 37.2% | 6.8E-32 | LAMB3 | laminin subunit beta 3 | |
| 4554 | -0.66 | - 36.9% | 7.0E-05 | CCN1 | cellular communication network factor 1 | |
| 1598 | -0.66 | - 36.8% | 7.8E-35 | TGFBI | transforming growth factor beta induced | |
| 1059 | -0.66 | - 36.6% | 6.7E-06 | TGFB2 | transforming growth factor beta 2 | |
| 2434231 | -0.65 | - 36.3% | 2.0E-09 | FN1 | fibronectin 1 |  |
| 33 | -0.65 | - 36.3% | 3.2E-05 | FBLN5 | fibulin 5 |  |
| 2362 | -0.65 | - 36.1% | 9.3E-15 | PRG4 | proteoglycan 4 (lubricin) | |
| 5736 | -0.64 | - 35.7% | 1.5E-14 | LTBP1 | latent TGF beta binding protein 1 | |
| 1060 | -0.64 | - 35.6% | 6.1E-22 | LGALS1 | galectin 1 |  |
| 654 | -0.62 | - 34.9% | 5.1E-23 | ABI3BP | ABI family member 3 binding protein | |
| 69196 | -0.59 | - 33.8% | 1.9E-15 | LUM | lumican |  |
| 52 | -0.59 | - 33.6% | 1.8E-04 | IBSP | integrin binding sialoprotein | |
| 240 | -0.57 | - 32.7% | 5.8E-03 | COL1A1 | collagen type I alpha 1 chain | |
| 166 | -0.57 | - 32.5% | 2.0E-08 | THBS3 | thrombospondin 3 | |
| 541 | -0.55 | - 31.6% | 5.5E-10 | TGFB1I1 | transforming growth factor beta 1 induced transcript 1 | |
| 117 | -0.54 | - 31.3% | 1.7E-07 | SEMA7A | semaphorin 7A (John Milton Hagen blood group) | |
| 4822 | -0.54 | - 31.0% | 5.5E-16 | PRELP | proline and arginine rich end leucine rich repeat protein | |
| 11475 | -0.54 | - 31.0% | 2.6E-11 | EDIL3 | EGF like repeats and discoidin domains 3 | |
| 2580 | -0.53 | - 30.9% | 1.6E-15 | COL27A1 | collagen type XXVII alpha 1 chain | |
| 69 | -0.53 | - 30.6% | 2.7E-03 | COL9A1 | collagen type IX alpha 1 chain | |
| 16885 | -0.53 | - 30.5% | 2.7E-05 | CSPG4 | chondroitin sulfate proteoglycan 4 | |
| 3564 | -0.53 | - 30.5% | 1.5E-07 | FBN1 | fibrillin 1 |  |
| 4242 | -0.51 | - 29.9% | 6.8E-08 | ANGPTL2 | angiopoietin like 2 | |
| 3685 | -0.50 | - 29.4% | 2.5E-09 | ANXA11 | annexin A11 |  |
| 120 | -0.49 | - 28.9% | 5.2E-05 | CCN3 | cellular communication network factor 3 | |
| 601 | -0.49 | - 28.8% | 3.9E-13 | IGFBP7 | insulin like growth factor binding protein 7 | |
| 13367 | -0.48 | - 28.5% | 1.5E-14 | COL6A1 | collagen type VI alpha 1 chain | |
| 313 | -0.48 | - 28.4% | 1.1E-10 | PCOLCE | procollagen C-endopeptidase enhancer | |
| 164 | -0.48 | - 28.4% | 8.0E-07 | SEMA3B | semaphorin 3B | |
| 14698 | -0.48 | - 28.2% | 5.8E-14 | COL11A2 | collagen type XI alpha 2 chain | |
| 43356 | -0.48 | - 28.1% | 1.6E-05 | ICAM1 | intercellular adhesion molecule 1 | |
| 642 | -0.47 | - 27.8% | 1.0E-14 | SLPI | secretory leukocyte peptidase inhibitor | |
| 13426 | -0.47 | - 27.8% | 1.7E-07 | HAPLN1 | hyaluronan and proteoglycan link protein 1 | |
| 103122 | -0.47 | - 27.7% | 1.9E-23 | DCN | decorin |  |
| 818 | -0.46 | - 27.4% | 5.2E-08 | PXDN | peroxidasin |  |
| 1226 | -0.44 | - 26.3% | 8.4E-10 | SDC3 | syndecan 3 |  |
| 4088 | -0.44 | - 26.2% | 2.8E-13 | LAMB2 | laminin subunit beta 2 | |
| 12065 | -0.44 | - 26.1% | 2.6E-03 | COL2A1 | collagen type II alpha 1 chain | |
| 1324 | -0.43 | - 25.8% | 9.8E-07 | LAMA4 | laminin subunit alpha 4 | |
| 1793 | -0.41 | - 24.6% | 1.3E-06 | COL1A2 | collagen type I alpha 2 chain | |
| 4463 | -0.40 | - 24.3% | 2.3E-07 | COL5A2 | collagen type V alpha 2 chain | |
| 412 | -0.39 | - 23.6% | 4.8E-10 | COL9A2 | collagen type IX alpha 2 chain | |
| 3862 | -0.39 | - 23.6% | 1.4E-19 | BGN | biglycan |  |
| 528 | -0.38 | - 23.0% | 9.8E-06 | LOXL2 | lysyl oxidase like 2 | |
| 309 | -0.38 | - 23.0% | 5.4E-06 | LAMA1 | laminin subunit alpha 1 | |
| 155 | -0.37 | - 22.6% | 1.3E-03 | CTHRC1 | collagen triple helix repeat containing 1 | |
| 35 | -0.37 | - 22.5% | 3.8E-02 | OMD | osteomodulin | |
| 1378 | -0.37 | - 22.5% | 2.6E-07 | TGFB1 | transforming growth factor beta 1 | |
| 1590 | -0.37 | - 22.4% | 2.7E-13 | MXRA7 | matrix remodeling associated 7 | |
| 872 | -0.36 | - 22.1% | 1.3E-04 | SNORC | secondary ossification center associated regulator of chondrocyte maturation | |
| 5021 | -0.36 | - 22.1% | 6.2E-09 | TGFBR2 | transforming growth factor beta receptor 2 | |
| 15010 | -0.35 | - 21.6% | 1.8E-12 | COL6A2 | collagen type VI alpha 2 chain | |
| 136 | -0.35 | - 21.6% | 4.1E-04 | OGN | osteoglycin |  |
| 223 | -0.35 | - 21.4% | 6.8E-04 | COL18A1 | collagen type XVIII alpha 1 chain | |
| 12415 | -0.34 | - 20.8% | 5.7E-08 | LTBP3 | latent TGF beta binding protein 3 | |
| 61 | -0.33 | - 20.7% | 1.3E-02 | LAMC2 | laminin subunit gamma 2 | |
| 138 | -0.33 | - 20.7% | 1.3E-04 | SSC5D | scavenger receptor cysteine rich family member with 5 domains | |
| 123 | -0.33 | - 20.6% | 1.2E-02 | WNT5B | Wnt family member 5B | |
| 476 | -0.33 | - 20.4% | 2.2E-07 | PLSCR1 | phospholipid scramblase 1 | |
| 805 | -0.33 | - 20.3% | 5.9E-08 | CILP | cartilage intermediate layer protein | |
| 186 | -0.32 | - 19.9% | 3.6E-05 | THBS4 | thrombospondin 4 | |
| 2416 | 0.43 | + 35% | 9.1E-04 | ANGPTL4 | angiopoietin like 4 | |
| 192 | 0.57 | + 49% | 1.5E-03 | GREM1 | gremlin 1, DAN family BMP antagonist | |
| 1850 | 0.71 | + 63% | 9.4E-34 | GPC1 | glypican 1 |  |
| 71 | 1.46 | x 2.8 | 4.9E-08 | ANGPT2 | angiopoietin 2 | |
|  |  |  |  |  |  |  |
