# Supplementary material for: Curcuma longa and Boswellia serrata Extracts Modulate Different and Complementary Pathways on Human Chondrocytes In Vitro: Deciphering of a Transcriptomic Study
Source: Front Pharmacol. 2022 Aug 11;13:931914. doi: 10.3389/fphar.2022.931914 (PMC9403192; doi:10.3389/fphar.2022.931914)
Supplement: Supplementary file 10 [file DataSheet1.DOCX]

***Supplementary file 1****: Patient’s characteristics*

| Sample | Sex | Age | RNA-seq | Supernatants | Flow cytometry |
| --- | --- | --- | --- | --- | --- |
| CR16 | M | 59 | X | X |  |
| CR17 | W | 65 |  | X |  |
| CR18 | W | 74 | X | X |  |
| CR19 | M | 59 | X | X |  |
| CR20 | M | 65 | X | X |  |
| CR21 | W | 54 |  | X |  |
| CR22 | W | 60 |  | X |  |
| CR23 | W | 67 | X | X |  |
| CR24 | W | 85 | X |  |  |
| CR25 | M | 72 | X | X |  |
| CR26 | M | 65 | X | X |  |
| CR27 | M | 56 | X | X |  |
| CR28 | M | 54 | X | X |  |
| 279 | W | 63 |  |  | X |
| 280 | W | 48 |  |  | X |
| 281 | W | 71 |  |  | X |
| 282 | W | 80 |  |  | X |
| 283 | W | 74 |  |  | X |
